# Lower *versus* higher oxygenation targets in critically ill patients with severe hypoxaemia: secondary Bayesian analysis to explore heterogeneous treatment effects in the Handling Oxygenation Targets in the Intensive Care Unit (HOT-ICU) trial

**DOI:** 10.1016/j.bja.2021.09.010

**Published:** 2021-10-19

**Authors:** Thomas L. Klitgaard, Olav L. Schjørring, Theis Lange, Morten H. Møller, Anders Perner, Bodil S. Rasmussen, Anders Granholm

**Affiliations:** 1 Department of Anaesthesia and Intensive Care, Aalborg University Hospital, Aalborg, Denmark; 2 Department of Clinical Medicine, Aalborg University, Aalborg, Denmark; 3 Collaboration for Research in Intensive Care, Copenhagen, Denmark; 4 Department of Public Health, Section of Biostatistics, University of Copenhagen, Copenhagen, Denmark; 5 Department of Intensive Care 4131, Rigshospitalet, University of Copenhagen, Copenhagen, Denmark

**Keywords:** Bayesian analysis, heterogeneity of treatment effects, intensive care unit, oxygen therapy, respiratory insufficiency

## Abstract

**Background:**

In the Handling Oxygenation Targets in the Intensive Care Unit (HOT-ICU) trial, a lower (8 kPa) *vs* a higher (12 kPa) PaO_2_ target did not affect mortality amongst critically ill adult patients. We used Bayesian statistics to evaluate any heterogeneity in the effect of oxygenation targets on mortality between different patient groups within the HOT-ICU trial.

**Methods:**

We analysed 90-day all-cause mortality using adjusted Bayesian logistic regression models, and assessed heterogeneous treatment effects according to four selected baseline variables using both hierarchical models of subgroups and models with interactions on the continuous scales. Results are presented as mortality probability (%) and relative risk (RR) with 95% credibility intervals (CrI).

**Results:**

All 2888 patients in the intention-to-treat cohort of the HOT-ICU trial were included. The adjusted 90-day mortality rates were 43.0% (CrI: 38.3–47.8%) and 42.3% (CrI: 37.7–47.1%) in the lower and higher oxygenation groups, respectively (RR 1.02 [CrI: 0.93–1.11]), with 36.5% probability of an RR <1.00. Analyses of heterogeneous treatment effects suggested a dose–response relationship between baseline norepinephrine dose and increased mortality with the lower oxygenation target, with 95% probability of increased mortality associated with the lower oxygenation target as norepinephrine doses increased.

**Conclusions:**

A lower oxygenation target was unlikely to affect overall mortality amongst critically ill adult patients with acute hypoxaemic respiratory failure. However, our results suggest an increasing mortality risk for patients with a lower oxygen target as the baseline norepinephrine dose increases. These findings warrant additional investigation.

**Clinical trial registration:**

NCT03174002.

**Editor's key points**

• Bayesian statistics can provide a valuable alternative perspective on clinical trial findings, particularly where knowing the most likely treatment effect can alter clinical practice even if this finding is not certain.
• The authors identified important differences in the effect of lower oxygenation targets between patient subgroups, which could be important in the care of critically ill adults.
• The possibility that critically ill patients in haemodynamic shock are more exposed to harm with lower oxygenation targets is important and should be investigated further in ongoing randomised trials.

Patients acutely admitted to the ICU with hypoxaemic respiratory failure are treated with supplemental oxygen. This treatment is believed to be life-saving, but the optimal target for oxygen therapy is not fully established. No firm conclusion on the benefits and harms of a lower *vs* a higher oxygenation target has been drawn for patients admitted to the ICU, as shown in a recently published systematic review.^1^ This may be because of limited data, or to a large degree of heterogeneity in published trials.

In the Normal Oxygenation Versus Hyperoxia in the Intensive Care Unit (OXYGEN-ICU) trial, a lower oxygenation strategy resulted in noticeably reduced ICU mortality compared with a higher oxygenation strategy in a mixed cohort of ICU patients (8.6 percentage points difference; 95% confidence interval [CI]: 1.7–15.0%), but the trial was stopped at an unplanned interim analysis after an earthquake.^2^ The Liberal Oxygenation Versus Conservative Oxygenation in ARDS (LOCO_2_) trial suggested benefit from a higher oxygenation strategy compared with a lower oxygenation strategy because of a reduced mortality at both 28 days (7.8 percentage points difference; 95% CI: –4.8 to 20.6) and 90 days post-randomisation (14.0 percentage points difference; 95% CI: 0.7–27.2%).^3^ However, this trial was also stopped early, as an unplanned interim analysis found observations of intestinal ischaemia, an unplanned secondary outcome, in the lower oxygenation group, but not in the higher oxygenation group. The Intensive Care Unit Randomized Trial Comparing Two Approaches to Oxygen Therapy (ICU-ROX) trial found no differences in 28-day ventilator-free days (–0.3 days absolute difference; 95% CI: –2.1 to 1.6 days) or in 90-day mortality (odds ratio [OR] 1.10; 95% CI: 0.84–1.44) between a lower and a higher oxygenation strategy.^4^ In the Handling Oxygenation Targets in the Intensive Care Unit (HOT-ICU) trial, adult patients with acute hypoxaemic respiratory failure in the ICU were randomised to an arterial partial pressure of oxygen (*P*aO_2_) of 8 kPa (lower target) or 12 kPa (higher target) during ICU admission.^5^ At 90 days, 42.9% of patients in the lower oxygenation group had died and 42.4% in the higher oxygenation group, resulting in an adjusted relative risk (RR) of 1.02 (95% CI: 0.94–1.11) in the primary frequentist analysis. Comparable results were found in the conventional subgroup analyses.^5^ However, heterogeneous treatment effects may still be present.^6^, ^7^, ^8^

Bayesian statistical methods allow for detailed probabilistic quantifications of effect sizes, and integration of prior knowledge allows for nuanced sensitivity analyses of the intervention effects. Such methods have previously been used in several large-scale trials to complement the conventional frequentist analysis^9^, ^10^, ^11^, ^12^ or as the primary statistical framework.^13^, ^14^, ^15^ In this prospective Bayesian analysis of the HOT-ICU trial,^16^ our aim was to provide a probabilistic evaluation of the effects of a lower oxygenation target *vs* a higher oxygenation target on 90-day all-cause mortality, to assess the probabilities of a number of pre-specified effect sizes, including effects larger than the *a priori* hypothesised 20% relative reduction in mortality,^17^^,^^18^ and to explore the presence of heterogeneous treatment effects on mortality based on pre-specified baseline variables.

## Methods

This secondary Bayesian analysis of the HOT-ICU trial was conducted in accordance with a protocol and statistical analysis plan published before randomisation of the last patient,^16^ and prepared according to recent recommendations.^6^^,^^8^^,^^19^^,^^20^ It was guided by the same principles as the Bayesian analysis of heterogeneous treatment effects in the Stress Ulcer Prophylaxis in the Intensive Care Unit (SUP-ICU) trial.^12^^,^^21^ The results are reported according to the Reporting of Bayes Used in clinical STudies (ROBUST) guideline,^22^ and this paper has been prepared in agreement with the Strengthening the Reporting of Observational Studies in Epidemiology statement.^23^

### HOT-ICU trial

The HOT-ICU trial was an investigator-initiated international, pragmatic, parallel-group, stratified, randomised trial (RCT), which enrolled patients from June 20, 2017 to August 3, 2020. Adult patients (≥18 yr), acutely admitted to the ICU with hypoxaemic respiratory failure, receiving a fraction of inspired oxygen (FiO_2_) of at least 0.50 in a closed system (invasive or noninvasive mechanical ventilation or mask/helmet CPAP) or at least oxygen 10 L min^−1^ in an open system, had an arterial line, and were expected to receive supplemental oxygen for at least 24 h in the ICU were included. Patients were randomised 1:1 to the lower oxygenation target or the higher oxygenation target, which was applied during the entire ICU stay, including readmissions, for up to 90 days. Additional details on the HOT-ICU trial, including exclusion criteria, approvals, and variable definitions, are available in the Supplementary Appendix and elsewhere.^5^^,^^17^^,^^18^

### Outcome measure

The primary outcome measure was 90-day all-cause mortality.

### Statistical analysis

All statistical analyses were performed using R version 4.0.4 (R Core Team, R Foundation for Statistical Computing, Vienna, Austria) and Stan^24^ through the *brms* R package,^25^^,^^26^ with additional details available in the Supplementary Appendix. We used Bayesian logistic regression models that incorporated prior distributions expressing pre-existing beliefs of effect sizes and their uncertainties in combination with data from the trial at hand. The models combined this to inform posterior distributions of the variables of interest.^27^ Posterior distributions were summarised using median values and percentile-based 95% credibility intervals (CrI) that may be interpreted as the 95% most probable values, conditional on the priors, models and data.^28^ The full posterior distributions were presented graphically, supplemented with probabilities of pre-specified and additional effect sizes.^16^ Results were presented as posterior adjusted risk ratios (RRs) and risk differences (RDs), and adjusted event probabilities in each group (used to calculate RRs and RDs), calculated by setting adjustment variables to their most common value, as specified in the protocol.^16^ We also present the results on the underlying odds ratio (OR) scale to facilitate comparison with other studies that may have reported on this scale. Relative risk and OR <1, and RD <0 favoured the lower oxygenation target; RR and OR >1, and RD >0 favoured the higher oxygenation target.

### Priors

For the primary analysis of the intervention effect, we used weakly informative priors centred on no difference (OR of 1=RR of 1) and including a large range containing all plausible effect sizes (ORs with 95% probability between 0.14 and 7.10). We thus expected the trial data to dominate the posterior probability distributions because of the large sample size of the HOT-ICU trial. Two pre-specified sensitivity analyses were conducted: (i) using evidence-based priors informed by an updated random-effects meta-analysis of previous RCTs, and (ii) using sceptic priors centred on no difference and sceptical of larger effect sizes, as described in the protocol.^16^ Full details on priors are presented in the Supplementary Appendix and in the protocol.^16^

### Subgroup-based heterogeneity of treatment effect analyses

We assessed the presence of heterogeneous treatment effects using four different subgrouping schemes based on selected baseline variables:

(i) Sequential Organ Failure Assessment (SOFA) score as a marker of organ dysfunction^29^
(ii) *P*aO_2_:FiO_2_ ratio as a marker of severity of hypoxaemic respiratory failure with additional adjustment for the type of oxygen supplementation system at baseline (closed or open), with closed system being the reference
(iii) Highest continuously infused dose of norepinephrine during the 24 h before randomisation
(iv) Latest plasma lactate concentration before randomisation

Five quintile-based subgroups were created of each variable ensuring that all patients with identical values were in the same groups. We used hierarchical Bayesian logistic regression models with partial pooling adjusted for the stratification variables (chronic obstructive pulmonary disease, haematological malignancy, and site) to calculate subgroup results.^26^^,^^30^ Results were presented using the effect measures outlined previously. Additional information on parameter definitions is available in the Supplementary Appendix and elsewhere.^5^

### Continuous heterogeneity of treatment effect analyses

We assessed the potential interactions of the allocation to the lower oxygenation target with the four baseline characteristics of interest for 90-day all-cause mortality on the continuous scale using Bayesian logistic regression models. All models were adjusted for the stratification variables mentioned previously. Additional adjustment for type of oxygen supplementation system (open or closed) at baseline was performed when assessing *P*aO_2_:FiO_2_ ratio. Results are presented using conditional effects plots with ORs and 95% CrI for interactions, and probabilities for interaction ORs <1 (negative interaction) and >1 (positive interaction). The conditional effects plots illustrate the predicted probabilities of an outcome dependent on the variables of interest (treatment, the baseline variable, and their interaction), with all other variables kept constant at their reference values (adjustment variables set to their most common values).

### Missing data and technical model details

We planned *a priori* to use complete case analysis if missingness for all variables in an analysis was less than 5% and multiple imputation otherwise.^16^ For all Bayesian models, we used four chains with 5000 warm-up and 5000 post-warm-up draws per chain, yielding 20 000 post-warm-up draws in all. For additional details on handling of missing data and model diagnostics, see the Supplementary Appendix and the protocol.^16^

## Results

We included 2888 of the 2928 patients (98.6%) randomised in the HOT-ICU trial, equivalent to the full intention-to-treat cohort.^5^ Baseline characteristics of the trial cohort are presented in Table 1. Additional characteristics of all subgroups according to quintiles and stratified according to treatment allocation are presented in Supplementary Tables 1a–4b. Diagnostics for all statistical models were acceptable.

**Table 1:** Baseline characteristics for all patients. Baseline characteristics for the trial cohort stratified by oxygenation target allocation. Numerical values are presented as medians with inter-quartile ranges (IQRs) and categorical variables as numbers (*n*) and percentages (%). FiO_2_, fraction of inspired oxygen; *P*aO_2_, partial pressure of arterial oxygen; SaO_2_, saturation of arterial oxygen; SOFA, Sequential Organ Failure Assessment. Additional baseline characteristics are available in the primary trial publication.^5^^∗^The *P*aO_2_:FiO_2_ ratio was missing in five patients in the lower oxygenation group and in seven patients in the higher oxygenation group. ^†^Plasma lactate concentration was missing in eight patients in the lower oxygenation group and in 11 patients in the higher oxygenation group. ^‡^The aggregated SOFA score ranges from 0 to 24, with sub-score from 0 to 4 for six organ systems (respiration, coagulation, liver, cardiovascular, CNS, and renal), with higher scores indicating higher degrees of organ failure. The SOFA score was missing in 44 patients in the lower oxygenation group and in 45 patients in the higher oxygenation group because of one or more missing sub-scores of the SOFA score.

Variable	Lower target, *n*=1441	Higher target, *n*=1447
Median age (IQR, yr)	70 (61–77)	70 (60–77)
Male sex, *n* (%)	916 (63.6)	939 (64.9)
Type of admission, *n* (%)		
Medical	1238 (85.9)	1233 (85.2)
Elective surgical	18 (1.3)	21 (1.5)
Emergency surgical	185 (12.8)	193 (13.3)
Chronic obstructive pulmonary disease	277 (19.2)	285 (19.7)
Active haematological cancer	81 (5.6)	86 (5.9)
Oxygen supplementation in a closed system, *n* (%)	1024 (71.1)	1038 (71.7)
Invasive mechanical ventilation, *n* (%)	826 (57.3)	863 (59.6)
Noninvasive ventilation or CPAP, *n* (%)	198 (13.7)	175 (12.1)
Oxygen supplementation in an open system, *n* (%)	417 (28.9)	409 (28.3)
Median *P*aO_2_ (IQR, kPa)	10.3 (8.7–12.6)	10.3 (8.7–12.3)
Median FiO_2_ (IQR)	0.70 (0.55–0.90)	0.70 (0.58–0.85)
Median *P*aO_2_:FiO_2_ ratio (IQR)^∗^		
In all systems	15.8 (11.8–21.0)	15.7 (12.0–20.5)
In closed systems	16.5 (12.2–21.7)	16.5 (12.6–21.4)
In open systems	14.1 (10.9–18.4)	13.9 (10.7–18.0)
Median lactate concentration (IQR, mM)^†^	1.8 (1.1–3.2)	1.7 (1.1–3.1)
Any use of vasopressors, *n* (%)	793 (55.0)	785 (54.3)
Median highest dose of norepinephrine (IQR, μg kg^−1^ min^−1^)	0.20 (0.10–0.40)	0.21 (0.10–0.40)
Median SOFA score (IQR)^‡^	8 (5–10)	8 (5–10)

### Bayesian analysis of 90-day all-cause mortality

**Fig 1:**
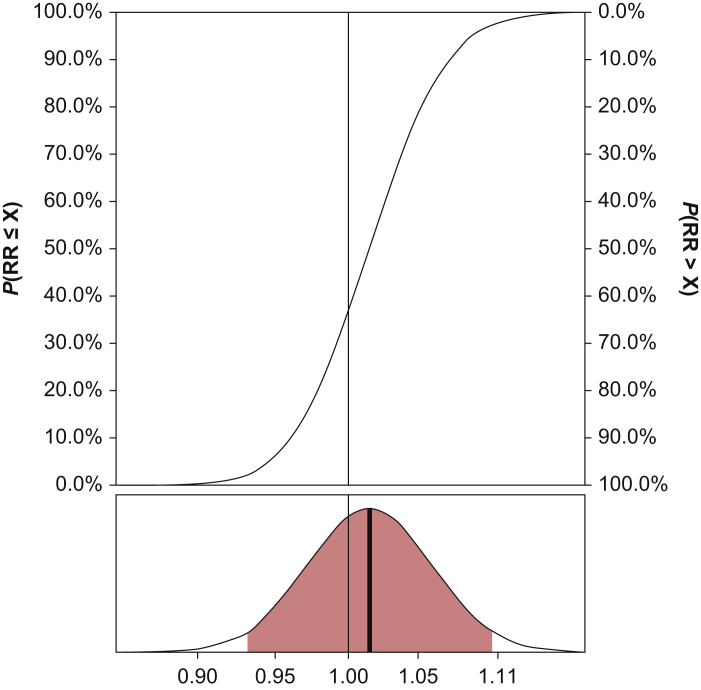
Posterior probability distribution for the adjusted relative risk (RR) for 90-day all-cause mortality in the primary analysis using *weakly informative* priors. Upper part: cumulative posterior probability distribution for the adjusted RR. *P*(RR ≤ X) is the probability that the RR is smaller or equal to any given value specified on the X-axis, being ‘X’; *P*(RR > X) is the probability that the RR is larger than any given value specified on the X-axis, being ‘X’. An RR <1 indicates benefit from the lower oxygenation target; an RR >1 indicates benefit of the higher oxygenation target. Lower part: full posterior probability distribution; full vertical line=median value; coloured area=95% credibility interval.

The adjusted RR for mortality was 1.02 (95% CrI: 0.93–1.11), with 63.5% probability of an RR >1.00. The probability of an RR <0.80, equivalent to the 20% *a priori* hypothesised relative mortality reduction,^17^ or more was <0.01%. We observed similar low probabilities (<2%) of such effect sizes across all subgroups, except for low plasma lactate concentrations (Supplementary Table 6). The full posterior probability distribution for 90-day all-cause mortality is presented in Fig. 1 (RD and OR distributions are presented in Supplementary Fig. 1a and b). Probabilities for mortality along with RRs and RDs for the trial cohort are presented in Table 2 (ORs are available in Supplementary Table 5).

**Fig 2:**
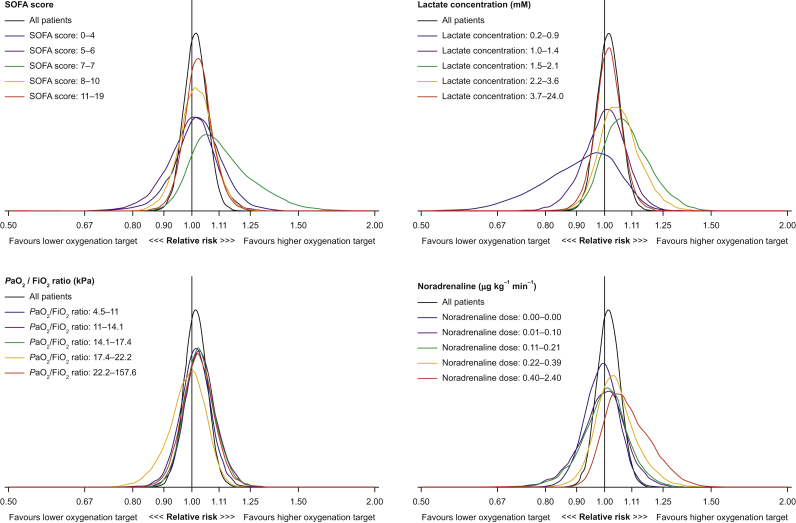
Posterior probability distributions of the adjusted relative risks (RRs) of the treatment effect on 90-day all-cause mortality according to the four pre-specified baseline variables in the primary analysis using weakly informative priors. The posterior probability distributions of RRs in each subgroup from the subgroup-based models are displayed together with the posterior distribution from the corresponding analysis of all patients not considering subgroups. An RR <1 indicates benefit from the lower oxygenation target; an RR >1 indicates benefit of the higher oxygenation target. *P*aO_2_:FiO_2_*F*iO_2_, ratio of partial pressure of arterial oxygen to fraction of inspired oxygen; SOFA, Sequential Organ Failure Assessment.

**Table 2:** Summarised effect measures for 90-day all-cause mortality. Adjusted posterior event probabilities, relative risks (RRs), and risk differences (RDs) for 90-day all-cause mortality in the primary analysis using weakly informative priors. CrI, credibility interval; SOFA, Sequential Organ Failure Assessment; *P*aO_2_:FiO_2_, ratio of partial pressure of arterial oxygen to fraction of inspired oxygen ratio; *n*, number of patients in each group (after excluding patients with missing data for one or more variables included in the analyses). RR <1 and RD <0 favour the lower target; RR >1 and RD >0 favour the higher target. ^∗^The SOFA score ranges from 0 to 24, with sub-score from 0 to 4 for six organ systems (respiration, coagulation, liver, cardiovascular, CNS, and renal), with higher aggregated scores indicating higher degrees of organ failure. ^†^*P*aO_2_:FiO_2_ ratio: lower scores indicate more severe pulmonary dysfunction.

Group	*n*	Event probability, lower target (%)	Event probability, higher target (%)	RR	RD (%)
All patients	2888	43.0 (95% CrI: 38.3–47.8)	42.3 (95% CrI: 37.7–47.1)	1.02 (95% CrI: 0.93–1.11)	0.6 (95% CrI: –3.0 to 4.3)
SOFA score (baseline)^∗^	2799				
0–4	486	32.5 (95% CrI: 26.5–39.1)	31.7 (95% CrI: 25.8–38.3)	1.03 (95% CrI: 0.85–1.23)	0.8 (95% CrI: –5.3 to 6.5)
5–6	501	35.5 (95% CrI: 29.3–42.1)	35.7 (95% CrI: 29.5–42.6)	1.00 (95% CrI: 0.81–1.16)	0.0 (95% CrI: –7.2 to 5.3)
7–7	352	37.6 (95% CrI: 30.5–45.7)	33.6 (95% CrI: 26.3–41.0)	1.10 (95% CrI: 0.94–1.48)	3.4 (95% CrI: –2.3 to 13.6)
8–10	881	42.1 (95% CrI: 36.3–48.0)	41.4 (95% CrI: 35.8–47.3)	1.02 (95% CrI: 0.89–1.15)	0.7 (95% CrI: –4.7 to 5.9)
11–19	579	57.2 (95% CrI: 50.7–63.5)	55.8 (95% CrI: 49.4–62.1)	1.02 (95% CrI: 0.92–1.15)	1.4 (95% CrI: –4.6 to 7.7)
Lactate concentration (baseline, mM)	2869				
0.2–0.9	501	23.1 (95% CrI: 17.5–29.2)	25.4 (95% CrI: 19.9–32.0)	0.92 (95% CrI: 0.66–1.14)	–1.9 (95% CrI: –10.0 to 3.1)
1.0–1.4	631	38.1 (95% CrI: 32.1–44.6)	38.0 (95% CrI: 32.0–44.6)	1.00 (95% CrI: 0.85–1.16)	0.2 (95% CrI: –6.3 to 5.8)
1.5–2.1	577	42.0 (95% CrI: 35.5–49.2)	38.7 (95% CrI: 32.2–45.3)	1.08 (95% CrI: 0.93–1.32)	3.1 (95% CrI: –2.7 to 11.1)
2.2–3.6	576	45.0 (95% CrI: 38.8–51.7)	42.5 (95% CrI: 36.0–49.1)	1.06 (95% CrI: 0.92–1.25)	2.3 (95% CrI: –3.5 to 9.6)
3.7–24.0	584	61.7 (95% CrI: 55.0–67.9)	60.8 (95% CrI: 54.2–67.0)	1.01 (95% CrI: 0.91–1.13)	0.9 (95% CrI: –5.5 to 7.1)
Norepinephrine dose (baseline, μg kg^−1^ min^−1^)	2888				
0.00–0.00	1373	38.1 (95% CrI: 33.0–43.5)	38.6 (95% CrI: 33.4–44.0)	0.99 (95% CrI: 0.87–1.11)	–0.4 (95% CrI: –5.3 to 4.0)
0.01–0.10	366	39.8 (95% CrI: 32.5–47.3)	40.1 (95% CrI: 33.2–47.3)	1.00 (95% CrI: 0.82–1.17)	–0.1 (95% CrI: –7.8 to 6.3)
0.11–0.21	372	39.5 (95% CrI: 32.4–47.0)	39.5 (95% CrI: 32.6–46.4)	1.01 (95% CrI: 0.83–1.19)	0.2 (95% CrI: –7.3 to 6.9)
0.22–0.39	348	50.0 (95% CrI: 42.4–57.6)	47.8 (95% CrI: 40.4–55.5)	1.04 (95% CrI: 0.91–1.24)	1.8 (95% CrI: –4.9 to 10.4)
0.40–2.40	429	52.4 (95% CrI: 45.3–60.2)	48.0 (95% CrI: 40.9–55.2)	1.08 (95% CrI: 0.95–1.33)	3.9 (95% CrI: –2.5 to 14.0)
*P*aO_2_:FiO_2_ ratio (baseline, kPa)^†^	2876				
4.5–11.0	565	46.0 (95% CrI: 39.8–52.4)	45.3 (95% CrI: 39.6–51.5)	1.02 (95% CrI: 0.90–1.14)	0.7 (95% CrI: –4.8 to 5.8)
11.0–14.1	584	46.6 (95% CrI: 40.4–53.3)	45.1 (95% CrI: 39.5–51.1)	1.03 (95% CrI: 0.92–1.17)	1.4 (95% CrI: –3.6 to 7.4)
14.1–17.4	574	46.6 (95% CrI: 40.5–53.1)	45.2 (95% CrI: 39.5–51.3)	1.03 (95% CrI: 0.92–1.16)	1.3 (95% CrI: –3.7 to 7.0)
17.4–22.2	577	41.6 (95% CrI: 34.8–48.3)	42.4 (95% CrI: 36.1–48.4)	0.99 (95% CrI: 0.84–1.11)	–0.5 (95% CrI: –7.2 to 4.5)
22.2–157.6	576	44.0 (95% CrI: 37.7–50.4)	43.0 (95% CrI: 36.9–48.8)	1.02 (95% CrI: 0.91–1.16)	1.0 (95% CrI: –4.2 to 6.5)

### Subgroup-based heterogeneity of treatment effect analyses

A substantial number of patients did not receive norepinephrine at baseline; these patients were all included in the same subgroup, which is thus larger than the remaining four quartile-based subgroups. The apparent overlap amongst *P*aO_2_:FiO_2_ ratio-based subgroup limits is attributable to rounding (Table 2).

For increasing baseline doses of norepinephrine, we found increasing risk for 90-day all-cause mortality, indicating benefit of the higher oxygenation target: from RR 0.99 (95% CrI: 0.87–1.11) in the lowest dosage group (all 0.00 mM) to RR 1.08 (95% CrI: 0.95–1.33) in the highest dosage group (0.40–2.40 mM). This potential dose–response relationship was not found in any of the other baseline variable subgrouping schemes. Posterior probabilities for mortality and the estimates of RRs and RDs in the four sets of subgroups are presented in Table 2 (ORs are presented in Supplementary Table 5). The posterior probability distribution plots of the RRs for mortality in the subgroups are presented in Fig. 2 (RD and OR distributions are presented in Supplementary Fig. 4a and b). The posterior probabilities for different RRs for all four sets of subgroups are presented in Supplementary Table 6. Comparisons of treatment effects in the subgroups are presented in Supplementary Tables 11–14.

### Continuous heterogeneity of treatment effect analyses

**Fig 3:**
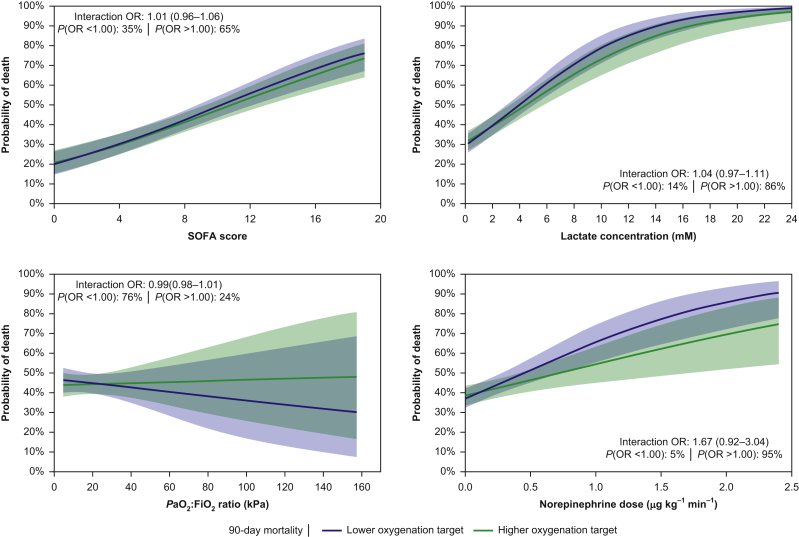
Conditional effects plots for 90-day all-cause mortality, using weakly informative priors. These plots illustrate the estimated interactions between treatment allocation and 90-day all-cause mortality on the continuous scale. The levels of the individual variables of interest are plotted on the X-axes; the probabilities of mortality are plotted on the Y-axes. Within each subplot, the odds ratio (OR) with 95% credibility interval for the interaction effect between the lower oxygenation target and the baseline variable assessed is presented. The posterior probabilities that the interaction OR is <1.00 (negative interaction) or >1.00 (positive interaction) are also presented. *P*aO_2_:*F*_io2_, ratio of partial pressure of arterial oxygen to fraction of inspired oxygen; SOFA, Sequential Organ Failure Assessment. In total, 95% of patients had a *P*aO_2_:*F*iO_2_ ratio <35.5 kPa.

We found a 95% probability of a positive interaction between increasing baseline norepinephrine dose and the lower oxygenation target on mortality (i.e. unfavourable effects of a lower oxygenation target with increasing dose of norepinephrine at baseline). For increasing baseline lactate concentrations, the probability of a positive interaction with the lower oxygenation target on mortality was 86% (i.e. potential increased mortality risk of the lower oxygenation target for patients with higher concentrations of lactate). The probabilities of positive interactions (i.e. potential increased mortality risks) between the lower oxygenation target and the remaining baseline variables were 65% for increasing baseline SOFA scores (i.e. higher degree of organ failure) and 76% for decreasing baseline *P*aO_2_:FiO_2_ ratios (i.e. greater severity of respiratory failure). Conditional effect plots showing the estimated interactions between treatment allocation and baseline variables on mortality on the continuous scale are presented in Fig. 3.

### Sensitivity analyses

The results of the sensitivity analyses using evidence-based and sceptic priors were largely consistent with the findings of the primary analysis (Supplementary Table 7; Supplementary Figs 2a–3c and 5a–7b).

### Missing data

No imputation of missing data was performed, as missingness was <5% for all variables of interest included in any analysis.^18^ For additional details on missing data, see the Supplementary Appendix and elsewhere.^5^

## Discussion

In this prospective, secondary analysis of treatment effects in the HOT-ICU trial, the risk of death within 90 days for patients treated with a lower oxygenation target was with 95% probability between RR 0.93 and 1.11. Given these data, larger effect sizes are improbable. Our analyses suggested heterogeneous treatment effects when considering the interaction between the lower oxygenation target and baseline norepinephrine dose, suggesting that in patients with higher degrees of shock (measured as higher administered doses of continuously infused norepinephrine), a lower oxygenation strategy may be harmful. This effect was consistent across a series of models. A similar trend was identified in the continuous model assessing plasma lactate concentrations at baseline, but without indications of the same relation in the subgroup-based heterogeneity analyses, and thus with no clear support for a dose–response relationship. Caution must be used when interpreting these findings, as the effect was only suggested in one of the two models. We found no strong suggestions of heterogeneous treatment effects according to SOFA scores or *P*aO_2_:FiO_2_ ratios at baseline.

The results of the Bayesian analysis of the 90-day all-cause mortality in this study are consistent with the primary frequentist analysis of the HOT-ICU trial,^5^ the ICU-ROX trial,^4^ and the latest meta-analysis conducted before the publication of the HOT-ICU trial.^1^ In contrast, the OXYGEN-ICU trial demonstrated benefit from a conservative oxygenation strategy,^2^ whilst the LOCO_2_ trial found potential benefit of a more liberal oxygenation strategy.^3^ However, given the substantially smaller sizes of the OXYGEN-ICU and LOCO_2_ trials (*n*=480 and 205, respectively) compared with the HOT-ICU (*n*=2928) and the ICU-ROX (*n*=1000) trials, and the fact that both were stopped after unplanned interim analyses, the findings of these trials may be attributable to chance. Also, the inclusion criteria of the trials differ substantially, as the ICU-ROX^4^ and LOCO_2_^3^ trials included only invasively mechanically ventilated patients, whereas the OXYGEN-ICU^2^ and HOT-ICU^5^ trials included patients on both open and closed oxygen supplementation systems. Additionally, when considering baseline *P*aO_2_:FiO_2_ ratios, patients presented with substantially more severe respiratory failure in the LOCO_2_^3^ and HOT-ICU^5^ trials compared with the ICU-ROX^4^ trial. These aspects may impede direct comparison of the results. Although larger effect sizes for mortality in the broad population of adult patients in the ICU with acute severe hypoxaemic respiratory failure seem improbable, smaller effects may also be of importance. Even a 2% absolute reduction in mortality would result in 2000 lives saved for every 100 000 patients treated with supplemental oxygen. The ongoing MEGA-ROX^31^ and UK-ROX^32^ trials are designed to assess absolute risk reductions for mortality of 1.5 and 2.5 percentage points, respectively, comparing a lower *vs* a higher oxygenation target. Effect sizes of such magnitudes cannot be excluded based on our results.

None of the aforementioned trials^2^, ^3^, ^4^ have considered the presence of heterogeneous treatment effects in a comparable manner to the one presented here. However, in a subgroup of patients with sepsis in the ICU-ROX trial, point estimates of treatment effects indicated harm of a lower oxygenation strategy, although this was not statistically significant.^33^ Similar was found in the subgroup of patients with shock at baseline in the HOT-ICU trial.^5^ On the contrary, the OXYGEN-ICU trial found reduced occurrence of shock when using a conservative oxygenation strategy compared with a more liberal oxygenation strategy.^2^

The strengths and limitations from the HOT-ICU trial are all carried over to this study.^5^ The most important strengths are the size of the trial, the pragmatic design, high external validity (35 ICUs in seven countries), and the clear separation in the oxygenation parameters between the intervention groups.^5^ Also, the protocol for this study was published before randomisation of the last patient in the HOT-ICU trial.^16^ Further, our results were consistent in the sensitivity analyses using different priors, and we evaluated the presence of heterogeneity of treatment effects both in subgroups and on the continuous scale, which may ease interpretation of our finding and serves as a consistency check. The limitations of this study are mainly related to the heterogeneity of treatment effect analyses. We chose the variables of interest based on availability and of the following reasons:^16^ the SOFA score is independently associated with mortality,^34^ and assessment of heterogeneity of treatment effects according to the risk of the outcome is recommended.^8^ Based on clinical rationale, different degrees of hypoxaemic respiratory failure may benefit from different levels of oxygenation; plasma lactate concentration and norepinephrine dose both serve as markers of shock, which, in turn, is associated with increased mortality.^35^ A dedicated prediction model for mortality would have been preferable, but this was not available. Also, other variables, or combinations of such, could have provided additional information on the potential heterogeneity with different oxygenation targets. As some subgroups may contain few events, this may lead to imprecision. Yet, this effect is to some extent mitigated by shrinkage and partial pooling in the hierarchical models.^26^^,^^30^ As the categorisation of the continuous baseline variables into quintile-based subgroups was data driven, cut-offs did not follow established conventions (e.g. in relation to the *P*aO_2_:FiO_2_ ratio), limiting the generalisability of the results. However, this was chosen to ensure that all subgroups were of adequate and similar sizes. In the analyses on the continuous scale, we assumed a linear relationship (on the log-OR scale) between the variables of interest and mortality, including the interaction term. For the sake of simplicity and to limit the risk of spurious findings and overfitting because of the use of multiple and increasingly flexible models, no other models to predict this relationship were applied. Lastly, secondary analyses and subgroup analyses should always be cautiously interpreted. Despite the analyses being pre-planned and the benefits of the Bayesian methods, the risks of spurious findings are not eliminated. All results from this study should consequently be regarded as hypothesis generating only.

In conclusion, the RR for 90-day all-cause mortality, when comparing a lower oxygenation target with a higher oxygenation target in adult patients in the ICU with acute hypoxaemic respiratory failure, was between 0.93 and 1.11 with 95% probability. Based on this, larger effect sizes are highly improbable. Our findings also suggest potentially important heterogeneity in treatment effects in terms of baseline norepinephrine dose as an index of haemodynamic shock. This increasing probability of death for patients treated with lower oxygenation targets as norepinephrine dose increases requires further investigation.

## Authors' contributions

Study conception: AG, TLK, OLS, MHM, AP, BSR

Statistical analysis plan and protocol: all authors

Involved in the Conducting of the Handling Oxygenation Targets in the Intensive Care Unit trial: all authors

Analyses: TLK, AG

Writing of first draft: TLK

Critical revision: all authors

Approval of paper: all authors
