# Supplementary material for: Lower versus higher oxygenation targets in critically ill patients with severe hypoxaemia: secondary Bayesian analysis to explore heterogeneous treatment effects in the Handling Oxygenation Targets in the Intensive Care Unit (HOT-ICU) trial
Source: Br J Anaesth. 2021 Oct 19;128(1):55–64. doi: 10.1016/j.bja.2021.09.010 (PMC8787771; doi:10.1016/j.bja.2021.09.010)
Supplement: Multimedia component 1 [file mmc1.docx]

**Lower vs higher oxygenation targets in ICU patients with severe hypoxaemia:** **secondary Bayesian analyses of mortality and heterogeneous treatment effects in the HOT-ICU trial**

Thomas L Klitgaard^1,2,5^, Olav L Schjørring^1,2,5^, Theis Lange^4,5^, Morten H Møller^3,5^, Anders Perner^3,5^,
Bodil S Rasmussen^1,2,5^, and Anders Granholm^3,5^

^1^ Department of Anaesthesia and Intensive Care, Aalborg University Hospital, Aalborg, Denmark

^2^ Department of Clinical Medicine, Aalborg University, Aalborg, Denmark

^3^ Department of Intensive Care 4131, Rigshospitalet, University of Copenhagen, Copenhagen

^4^ Department of Public Health, Section of Biostatistics, University of Copenhagen, Copenhagen, Denmark

^5^ Collaboration for Research in Intensive Care (CRIC), Copenhagen, Denmark

**Supplementary Appendix**

**Table of contents**

Approvals 4

Enrolment criteria 4

Outcome and baseline parameter definitions 5

Model definitions, priors, technical details and model diagnostics 7

Missing data 10

Table S1a Baseline characteristics in the SOFA score-based subgroups 11

Table S1b Baseline characteristics in the SOFA score-based subgroups stratified by treatment 13

Table S2a Baseline characteristics in the lactate concentration-based subgroups 15

Table S2b Baseline characteristics in the lactate concentration-based subgroups stratified by treatment 17

Table S3a Baseline characteristics in the norepinephrine dose-based subgroups 19

Table S3b Baseline characteristics in the norepinephrine dose-based subgroups stratified by treatment 21

Table S4a Baseline characteristics in the PaO_2_/FiO_2_ ratio-based subgroups 23

Table S4b Baseline characteristics in the PaO_2_/FiO_2_ ratio-based subgroups stratified by treatment 25

Table S5 Odds ratios for 90-day all-cause mortality in the primary analysis using weakly informative priors 27

Table S6 Posterior probabilities of different effect sizes for 90-day all-cause mortality 28

Table S7 Summarised effect measures for 90-day all-cause mortality in the sensitivity analysis using evidence-based priors 29

Table S8 Posterior probabilities of different effect sizes of 90-day all-cause mortality in the sensitivity analysis using evidence-based priors 30

Table S9 Summarised effect measures for 90-day all-cause mortality in the sensitivity analysis using sceptic priors 31

Table S10 Posterior probabilities of different effect sizes of 90-day all-cause mortality in the sensitivity analysis using sceptic priors 32

Table S11 Comparison of treatment effects in baseline SOFA score-based subgroups 33

Table S12 Comparison of treatment effects in baseline plasma lactate concentration-based subgroups 34

Table S13 Comparison of treatment effects in baseline norepinephrine dose-based subgroups 35

Table S14 Comparison of treatment effects in baseline PaO_2_/FiO_2_ ratio-based subgroups 36

Fig. S1a Posterior probability distribution of the adjusted risk difference for 90-day all-cause mortality in the primary analysis using weakly informative priors 37

Fig. S1b Posterior probability distribution of the adjusted odds ratio for 90-day all-cause mortality in the primary analysis using weakly informative priors 38

Fig. S2a Posterior probability distribution of the adjusted relative risk for 90-day all-cause mortality in the sensitivity analysis using evidence-based priors 39

Fig. S2b Posterior probability distribution of the adjusted risk difference for 90-day all-cause mortality in the sensitivity analysis using evidence-based priors 40

Fig. S2c Posterior probability distribution of the adjusted odds ratio for 90-day all-cause mortality in the sensitivity analysis using evidence-based priors 41

Fig. S3a Posterior probability distribution of the adjusted relative risk for 90-day all-cause mortality in the sensitivity analysis using sceptic priors 42

Fig. S3b Posterior probability distribution of the adjusted risk difference for 90-day all-cause mortality in the sensitivity analysis using sceptic priors 43

Fig. S3c Posterior probability distribution of the adjusted odds ratio for 90-day all-cause mortality in the sensitivity analysis using sceptic priors 44

Fig. S4a Posterior probability distributions of the adjusted risk differences of the treatment effect on 90-day all-cause mortality in the four sets of subgroups in the primary analysis using weakly informative priors 45

Fig. S4b Posterior probability distributions of the adjusted odds ratios of the treatment effect on 90-day all-cause mortality in the four sets of subgroups in the primary analysis using weakly informative priors 46

Fig. S5a Posterior probability distributions of the adjusted relative risks of the treatment effect on 90-day all-cause mortality in the four sets of subgroups in the sensitivity analysis using evidence-based priors 47

Fig. S5b Posterior probability distributions of the adjusted risk differences of the treatment effect on 90-day all-cause mortality in the four sets of subgroups in the sensitivity analysis using evidence-based priors 48

Fig. S5c Posterior probability distributions of the adjusted odds ratios of the treatment effect on 90-day all-cause mortality in the four sets of subgroup in the sensitivity analysis using evidence-based priors 49

Fig. S6a Posterior probability distributions of the adjusted relative risks of the treatment effect on 90-day all-cause mortality in the four sets of subgroups in the sensitivity analysis using sceptic priors 50

Fig. S6b Posterior probability distributions of the adjusted risk differences of the treatment effect on 90-day all-cause mortality in the four sets of subgroups in the sensitivity analysis using sceptic priors 51

Fig. S6c Posterior probability distributions of the adjusted odds ratios of the treatment effect on 90-day all-cause mortality in the four sets of subgroups in the sensitivity analysis using sceptic priors 52

Fig. S7a Conditional effects plot for 90-day all-cause mortality in the sensitivity analysis using evidence-based priors 53

Fig. S7b Conditional effects plot for 90-day all-cause mortality in the sensitivity analysis using sceptic priors 54

STROBE checklist 55

References 57

## Approvals

The Handling Oxygenation Targets in the Intensive Care Unit (HOT-ICU) trial was approved by the Committee on Health Research Ethics in the North Denmark Region (N-20170015) with additional national/local ethics approvals as required, the Danish Medicines Agency (2017021858), and the Danish Data Protection Agency (2008-58-01028). The trial was prospectively registered at ClinicalTrials.gov (NCT-03174002) and EudraCT (AAUH-ICU-01, 2017-000632-34). No additional approvals were required for this secondary analysis of the trial.

## Enrolment criteria

For additional details, please see the published protocol^1^ and primary trial publication.^2^

Trial inclusion criteria

- Acute admission to the intensive care unit (ICU)
- Age ≥ 18 years
- Supplemental oxygen therapy fulfilling one of the following criteria:
- Flow of oxygen ≥ 10 litres/minute in an open system irrespective of any flow of atmospheric air, including high-flow systems
- Fraction of inspired oxygen (FiO_2_) ≥ 0.50 in a closed system (invasive or non-invasive (mask or helmet) ventilation, or continuous positive airway pressure (CPAP) systems (mask or helmet))
- Expected duration of supplemental oxygen ≥ 24 hours in the ICU
- Arterial line in place for monitoring partial pressure of arterial oxygen (PaO_2_)

Trial exclusion criteria

- Cannot be randomised within 12 hours of ICU admission
- Chronic mechanical ventilation (invasive mechanical ventilation, continuous non-invasive ventilation, or continuous mask-CPAP) for any reason except nocturnally for sleep apnoea and/or obesity hypoventilation syndrome
- Use of home supplementary oxygen
- Previous bleomycin treatment
- Solid organ transplant planned or performed during current hospitalisation
- Withdrawal from active therapy or brain death deemed imminent
- Pregnancy (positive urine or plasma human chorionic gonadotropin)
- Carbon monoxide poisoning
- Cyanide poisoning
- Paraquat poisoning
- Methaemoglobinaemia
- Sickle cell disease
- Any condition expected to involve the use of hyperbaric oxygen treatment
- Consent not obtainable according to national regulations
- Previously randomised into the HOT-ICU trial

## Outcome and baseline parameter definitions

Primary outcome

Ninety-day all-cause mortality: death from any cause within 90 days after randomisation.

Baseline parameters

*Comorbidities:*

- Ischaemic heart disease: any history of either myocardial infarction, previous coronary intervention, or stable or unstable angina pectoris or use of nitrates indicating this condition
- Heart failure: left ventricular ejection fraction ≤ 40%, or diagnosed chronic heart failure with preserved left ventricular ejection fraction
- Metastatic cancer: any metastases from a malignant non-haematological neoplasm that is not considered eradicated
- Chronic dialysis: receiving any renal replacement therapy on a regular basis
- Chronic obstructive pulmonary disease (COPD): a spirometry in stable phase diagnostic of COPD (i.e. forced expiratory volume in one second/forced vital capacity (FEV_1_/FVC) < 0.7 and FEV_1_ < 80% of predicted level and flow limitations being incompletely reversible with inhaled bronchodilators), or anamnestic diagnosis of COPD with concomitant use of inhaled beta_2_-adrenergics and/or inhaled anticholinergics and/or inhaled glucocorticoids
- Active haematological malignancy: any intervention within the last six months against any of the following: leukaemia (acute lymphoblastic leukaemia, acute myelogenous leukaemia, chronic lymphocytic leukaemia, hairy cell leukaemia, T-cell prolymphocytic leukaemia, B-cell prolymphocytic, large granular lymphocytic leukaemia), lymphoma (Hodgkin’s lymphomas, Non-Hodgkin’s lymphoma (e.g. small lymphocytic lymphoma, lymphoblastic lymphoma, diffuse large B-cell lymphoma, follicular lymphoma, mantle cell lymphoma, marginal zone lymphoma, Burkitt’s lymphoma, post-transplant lymphoproliferative disorder, Waldenström’s macroglobulinemia, NK- and T-cell lymphomas), multiple myeloma/plasma cell myeloma, solitary plasmacytoma, myelodysplastic syndromes, myeloproliferative neoplasms (e.g. chronic myelogenous leukaemia, chronic neutrophilic leukaemia, primary myelofibrosis, myeloproliferative neoplasm, unclassifiable, mast cell diseases), other (rare) malignant lymphoid and myeloid diseases, or benign haematological diseases (aplastic anaemia, autoimmune haemolytic anaemia)

*Acute illnesses:*

- Pneumonia as defined by the clinician
- Multiple trauma: acute accident resulting in injuries to tissues at two or more anatomical sites
- Stroke: onset of symptoms prior to randomisation and verified cerebral bleeding, ischaemia, or embolism on computed tomography (CT) or magnetic resonance imaging (MRI), or diagnosed by a neurologist
- Traumatic brain injury: verified by fresh lesions on CT or MRI
- Myocardial infarction: diagnostic electrocardiographic changes, significant rise in coronary biomarkers, acute percutaneous coronary intervention, and/or acute coronary bypass grafting conducted during current hospitalisation
- Intestinal ischaemia: onset of gastric, mesenteric, or colonic ischaemia verified during current hospitalisation by exploratory or diagnostic abdominal surgery, gastroscopy or colonoscopy, or findings of intestinal ischaemia on CT or MRI angiography
- Cardiac arrest: clinically diagnosed with initiated cardio-pulmonary resuscitation leading to or occurred during current ICU admission
- Acute respiratory distress syndrome (ARDS): according to the Berlin criteria ^3^ as bilateral chest infiltrates on x-ray, a PaO_2_/FiO_2_ ratio below 40 kPa in mechanically ventilated patients with positive end-expiratory pressure ≥ 5 cm of H_2_O or CPAP ≥ 5 cm H_2_O. The patient’s respiratory failure must not have been fully explainable by cardiac failure or fluid overload

Sequential Organ Failure Assessment (SOFA) score

Data for the SOFA score^4^ was assessed in the 24 hours before randomisation, except for the respiration score, which was assessed at randomisation. The aggregated SOFA score ranges from 0-24, with sub-scores ranging from 0 to 4 for each of 6 components (respiration, coagulation, liver, cardiovascular, central nervous system, and renal). Higher scores indicate more severe organ failure.

## Model definitions, priors, technical details and model diagnostics

**Model definitions**

All statistical models were run using R version 4.0.4 (R Core Team, R Foundation for Statistical Computing, Vienna, Austria) and Stan^5^ through the *brms*^6,7^ R package version 2.15.0, with *rstan* version 2.21.2 as the backend. All analyses were conducted in accordance with our published protocol.^8^ In these analyses we used logistic regression models (*family = bernoulli(link = “logit”)* in *brms*) with adjustment for the same stratification variables as in the primary analysis (know chronic obstructive pulmonary disease (COPD), active haematological malignancy, and site). We present results as adjusted relative risks (RRs), and risk differences (RDs) calculated for reference patients as stated in the primary text and the protocol.^8^ To ease comparison with other trials we also decided to present the results on the odds ratio (OR) scale.

*Secondary analysis of the primary outcome:*

The models for the primary outcome were specified as (brms syntax):

*mortality ~ 1 + treatment + site + COPD + haema_malign*

With *mortality* denoting the primary outcome of 90-day all-cause mortality, *1* is the intercept (corresponding to the baseline risk for patients in the reference group for all the following variables; all intercepts were regular, non-centred intercepts), *treatment* denoting the intervention (being the lower oxygenation target, with the higher oxygenation target as the reference), *site* representing the recruiting site (with the site including most patients being the reference), *COPD* denoting known chronic obstructive pulmonary disease (with patients without known COPD being the reference), and *haema_malign* representing active haematological malignancy (reference was patients without active haematological malignancy).

*Subgroup-based heterogeneity of treatment effect analyses:*

All models for the subgroup-based heterogeneity of treatment effect analyses were specified as (brms syntax) as:

*mortality ~ 1 + treatment + (1 + treatment || subgroup) + site + COPD + haema_malign + system_type*

Treatment effect and baseline risk were estimated separately for each of the predefined subgroups (“random effects”) in addition to the population-level (“fixed effect”) using the *(1 + treatment || subgroup)* statement. When analysing the subgroups defined as per baseline PaO_2_/FiO_2_ ratios, additional adjustment for the type of oxygen supplementation system used at baseline (with closed system (invasive, non-invasive or mask/helmet CPAP) being the reference group) was encompassed using *system_type*; this term was not included for the other subgroup analyses. Any additional variables had identical meanings as stated above.

*Continuous heterogeneity of treatment effect analyses:*

All models for the analyses of heterogeneity of treatment effect on the continuous scale were specified as (*brms* syntax):

*mortality ~ 1 + treatment + variable + treatment:variable + site + COPD + haema_malign + system_type*

The parameter of interest (defined as *variable*: either baseline SOFA score, baseline PaO_2_/FiO_2_ ratio, baseline norepinephrine dose, or baseline plasma lactate concentration) was encompassed as a linear effect (on the log OR scale) and also per an interaction term with the intervention effect *(treatment:variable*). As specified above, *system_type* denotes adjustment for the type of oxygen supplementation system used at baseline (open or closed) and is only included in the models assessing baseline PaO_2_/FiO_2_ ratios.

**Priors – general principles**

Priors were specified on the log-odds ratio scale (as used by the logistic regression models) and described using odds ratios here. Results are primarily presented on the RR scale and secondarily on the RD and OR scales, as outlined above. For the primary analyses we used *weakly informative* priors centred on no difference (OR = 1.00, corresponding to an RR = 1 and an RD = 0) and encompassing all plausible effect sizes. Due to the large size of the HOT-ICU trial we expected the data (via the likelihood function) to dominate the posterior distributions.^8^ For the variables of main interest, we used different priors in two pre-specified sensitivity analyses as described below (*evidence-based* priors and *sceptic* priors). For all adjustment parameters not of primary interest we used the same *weakly informative* priors in all analyses.

*Bayesian analysis of 90-day all-cause mortality:*

We used the following priors (on the log-odds ratio scale):

- Intercept: *normal*(-1.1, 1.5) prior (a normally distributed prior with mean -1.1 and standard deviation (SD) 1.5), equivalent to a baseline risk that with 95% probability is between 2% and 86%, with a mean probability of 25%, conforming to the control group risk used in the trial sample size calculations.^1,9^
- Stratification variables (known COPD, active haematological malignancy, and site): *normal*(0, 1), equivalent to ORs that with 95% probability are between 0.14 and 7.10, centred on 1.00 (i.e. no difference).
- Intervention effect:
- Primary analysis: a *normal*(0, 1) prior was used as described above.
- Sensitivity analysis using an *evidence-based* prior: a *normal*(-0.068, 0.130) prior was used, equivalent to an OR that with 95% probability is between 0.72 and 1.20, with a mean of 0.93. This prior favours a lower target, and is based on an updated conventional random-effects meta-analysis of previous studies, as described in our protocol.^8^
- Sensitivity analysis using a *sceptic* prior: a *normal*(0, 0.15) prior was used, equivalent to an OR that with 95% probability is between 0.75 and 1.34, with a mean of 1.00. This prior favours results close to *no difference* and is sceptical of large effect sizes.

*Subgroup based heterogeneity of treatment effect analyses:*

We used the following priors the subgroup based heterogeneity of treatment effect analyses:

- For all priors also encompassed in the analysis of the primary outcome, similar priors were used as stated previously.
- We used the same prior for type of oxygen supplementation system at baseline as specified above.
- Group-level intercepts and treatment effects:
- For the primary analysis and sensitivity analysis using *evidence-based* priors we used *normal*(0, omega) priors. Omega is the shrinkage factor which is estimated from the data by ways of a *half-normal* prior with SD of 1, corresponding to a standard deviation (SD) that with 95% probability is between 0.031 and 2.24 with a mean of 0.80 and a median of 0.67.
- For the sensitivity analysis using *sceptic* priors we used a half-normal prior with SD 0.35 for omega, equivalent to an SD which with 95% probability is between 0.011 and 0.78 and has a mean of 0.28 and a median of 0.24.

*Continuous heterogeneity of treatment effect analyses:*

We used the following priors for the heterogeneity of treatment effect analyses conducted on the continuous scale:

- For all priors also encompassed in the analysis of the primary outcome, similar priors were used as stated previously.
- For the analyses of baseline PaO_2_/FiO_2_ ratios, a *normal*(0, 1) prior was used for the type of oxygen supplementation system adjustment (with a closed system as the reference group).
- For all analyses, including sensitivity analyses, we used *weakly informative* priors for the effect of the parameters of interest (SOFA score, PaO_2_/FiO_2_ ratio, norepinephrine dose, and plasma lactate concentration) and the interaction between these and the treatment effect. Since different scales were used for the four variables, priors were defined as *normal*(0, x) with x being 1 scaled by division with the SD of the parameter of interest. With each increment of the SD of the parameter at interest, this corresponds to ORs that with 95% probability are between 0.14 and 7.10, centred on 1.00.

**Technical details and model diagnostics**

Four chains (using the no U-turn sampler) with 5,000 warmup and 5,000 post-warmup draws per chain (20,000 post-warmup draws in total) and approximately 6,000-40,000 bulk and 8,000-15,000 tail effective sample sizes for the parameters of interest with no divergent transitions were used. We had a-priori defined to require an effective sample size of at least 1,000 for the parameters of interest.^8^ Chain convergence was assessed by visual inspection of overlain trace and density plots and by the updated Rhat statistic^10,11^ which we required to be ≤ 1.01 for all parameters.^11^ Model fits were assessed using graphical posterior predictive checks^12^ and Pareto-smoothed importance sampling leave-one-out cross-validation^13,14^, primarily focused on the effective number of parameters (*p_loo*) compared to the actual number of parameters in the models. All models were adequate according to these diagnostics.

## Missing data

In the HOT-ICU trial 2928 patients were enrolled. Of these, 21 patients in the lower oxygenation group had missing information for the primary outcome of 90-day all-cause mortality due to withdrawal or unobtainable consent for the use of data (20 patients) or loss to follow-up (1 patient). In the higher oxygenation 19 patients were missing due to withdrawal or unobtainable consent for the use of data (18 patients) or loss to follow-up (1 patient). This was equivalent to 1.4% (40/2928) of the trial population, leaving 2888 cases used in this secondary Bayesian analysis of the primary outcome.

Additionally, the aggregated SOFA score was missing in 44 patients in the lower oxygenation group, and in 45 patients in the higher oxygenation group due to one or more missing sub-scores of the SOFA score, leaving 2799 patients (95.6%) for the analyses including SOFA scores.

Baseline lactate concentration was missing in 8 patients in the lower oxygenation group, and in 11 patients in the higher oxygenation group, leaving 2869 patients (98.0%) for the analyses including baseline lactate.

No baseline dose of norepinephrine was missing, leaving 2888 patients (98.6%).

Baseline PaO_2_/FiO_2_ ratio was missing in 5 patients in the lower oxygenation group, and in 7 patients in the higher oxygenation group, leaving 2876 patients (98.2%) for the analyses including baseline PaO_2_/FiO_2_ ratios.

In accordance to the protocol for this study,^8^ no imputation was performed as the percentage of patients with missing values for one or more variables included in an analysis was less than 5% for all analyses.

## Table S1a Baseline characteristics in the SOFA score-based subgroups

| SOFA score range^a^ | All patients^b^ n = 2888 | SOFA score: 0-4 n = 486 | SOFA score: 5-6 n = 501 | SOFA score: 7-7  n = 352 | SOFA score: 8-10 n = 881 | SOFA score: 11-19 n = 579 |
| --- | --- | --- | --- | --- | --- | --- |
| Median age (IQR) - years | 70 (61 - 77) | 69 (59 - 77) | 71 (63 - 78) | 71 (62 - 78) | 70 (61 - 77) | 68 (59 - 75) |
| Male sex, n (%) | 1855 (64.2) | 305 (62.8) | 325 (68.9) | 222 (63.1) | 567 (64.4) | 382 (66.0) |
| Median interval from hospital admission to randomisation (IQR) - days | 1 (0 - 5) | 2 (0 - 5) | 2 (1 - 6) | 1 (0 - 4) | 1 (0 - 4) | 1 (0 - 5) |
| Median interval from ICU admission to randomisation (IQR) - hours | 4 (2 - 7) | 2 (1 - 5) | 3 (1 - 6) | 4 (2 - 7) | 4 (2 -8) | 4 (2 - 8) |
| Chronic co-morbidities, n (%) |  |  |  |  |  |  |
| Ischaemic heart disease | 409 (16.2) | 74 (15.2) | 62 (12.4) | 53 (15.1) | 117 (13.3) | 88 (15.2) |
| Chronic heart failure | 285 (9.9) | 53 (10.9) | 55 (11.0) | 35 (9.9) | 73 (8.3) | 59 (10.2) |
| Active metastatic cancer | 126 (4.4) | 20 (4.1) | 20 (4.0) | 16 (4.6) | 45 (5.1) | 20 (3.5) |
| Chronic dialysis | 47 (1.6) | 7 (1.4) | 3 (0.6) | 4 (1.1) | 17 (1.9) | 16 (2.8) |
| Chronic obstructive  pulmonary disease | 562 (19.5) | 101 (20.8) | 111 (22.2) | 83 (23.6) | 164 (18.6) | 84 (14.5) |
| Active haematological   cancer | 167 (5.8) | 13 (2.7) | 33 (6.6) | 12 (3.4) | 54 (6.1) | 52 (9.0) |
| Type of admission, n (%) |  |  |  |  |  |  |
| Medical | 2471 (85.6) | 446 (91.8) | 450 (89.8) | 276 (78.4) | 725 (82.3) | 496 (85.7) |
| Elective surgical | 39 (1.4) | 8 (1.7) | 5 (1.0) | 5 (1.4) | 13 (1.5) | 4 (0.7) |
| Emergency surgical | 378 (13.1) | 32 (6.6) | 46 (9.2) | 71 (20.2) | 143 (16.2) | 79 (13.6) |
| Acute illness, n (%) |  |  |  |  |  |  |
| Pneumonia | 1664 (57.6) | 309 (63.6) | 330 (65.9) | 181 (51.4) | 494 (56.1) | 295 (51.0) |
| Multiple trauma | 52 (1.8) | 8 (1.7) | 13 (2.6) | 8 (2.3) | 15 (1.7) | 4 (0.7) |
| Haemorrhagic or ischaemic   stroke | 47 (1.6) | 2 (0.4) | 6 (1.2) | 7 (2.0) | 17 (1.9) | 13 (2.3) |
| Traumatic brain injury | 22 (0.8) | 3 (0.6) | 5 (1.0) | 1 (0.3) | 4 (0.5) | 8 (1.4) |
| Myocardial infarction | 182 (6.3) | 28 (5.8) | 24 (4.8) | 27 (7.7) | 53 (6.0) | 46 (7.9) |
| Intestinal ischaemia | 68 (2.4) | 5 (1.0) | 9 (1.8) | 9 (2.6) | 29 (3.3) | 16 (2.8) |
| Cardiac arrest | 332 (11.5) | 0 (0.0) | 6 (1.2) | 10 (2.8) | 104 (11.8) | 201 (34.7) |
| ARDS | 366 (12.7) | 54 (11.1) | 60 (12.0) | 33 (9.4) | 120 (13.6) | 95 (16.4) |
| Closed system, n (%) | 2062 (71.4) | 244 (50.2) | 267 (53.3) | 265 (75.3) | 700 (79.5) | 529 (91.4) |
| Invasive mechanical   ventilation, n (%) | 1689 (58.5) | 119 (24.5) | 157 (31.3) | 228 (64.8) | 628 (71.3) | 512 (88.4) |
| Median tidal volume   (IQR) – ml | 499 (429 - 574) | 502 (429 - 609) | 482 (421 - 539) | 503 (432 - 594) | 491 (419 - 570) | 507 (438 - 579) |
| Median end expiratory   pressure (IQR) - cm H_2_O | 9 (7 - 10) | 8 (6 - 10) | 9 (7 - 10) | 9 (7 - 10) | 9 (7 - 10) | 9 (7 - 10) |
| Median peak inspiratory   pressure (IQR) - cm H_2_O | 25 (21 - 29) | 24 (20 - 29) | 23 (20 - 29) | 24 (20 - 28) | 25 (20 - 29) | 26 (21 - 32) |
| Non-invasive ventilation or   CPAP, n (%) | 373 (12.9) | 125 (25.7) | 110 (22.0) | 37 (10.5) | 72 (8.2) | 17 (2.9) |
| Median end expiratory   pressure (IQR) - cm H_2_O | 7 (5 - 9) | 7 (5 - 8) | 8 (6 -10) | 8 (6 - 8) | 7 (5 - 8) | 7 (6 - 8) |
| Open system, n (%) | 826 (28.6) | 242 (49.8) | 234 (24.7) | 87 (24.7) | 181 (20.5) | 50 (8.6) |
| Median PaO_2_ (IQR) - kPa | 10.3 (8.7 - 12.5) | 10.4 (8.7 - 12.9) | 9.8 (8.4 - 11.9) | 10.8 (9.2 - 13.3) | 10.1 (8.8 - 12.3) | 10.5 (8.1 - 12.3) |
| Median SaO_2_ (IQR) - % | 94 (91 - 97) | 95 (92 - 97) | 94 (91 - 97) | 95 (92 - 97) | 94 (91- 97) | 95 (91 - 97) |
| Median FiO_2_ (IQR) - fraction | 0.70 (0.55 - 0.85) | 0.60 (0.50 - 0.75) | 0.70 (0.54 - 0.85) | 0.60 (0.60 - 0.70) | 0.70 (0.60 - 0.90) | 0.74 (0.60 - 0.90) |
| Median PaO_2_/FiO_2_ ratio (IQR) |  |  |  |  |  |  |
| In all systems | 15.7 (11.9 - 20.8) | 17.0 (14.0 - 22.2) | 14.7 (11.1 - 20.6) | 17.4 (14.0 - 21.8) | 15.1 (11.1 - 20.0) | 15.0 (11.5 - 20.0) |
| In closed systems | 16.6 (12.4 – 21.6) | 18.7 (15.4 - 26.9) | 16.5 (12.4 - 24.0) | 17.8 (15.0 - 22.6) | 15.6 (11.6 - 20.6) | 17.5 (11.1 - 21.4) |
| Median lactate concentration (IQR) - mM | 1.8 (1.1 - 3.2) | 1.3 (0.9 - 2.0) | 1.3 (0.9 -2.3) | 1.8 (1.2 - 1.9) | 1.9 (1.2 - 3.5) | 2.8 (1.6 - 5.5) |
| Median lowest mean arterial pressure (IQR) - mmHg | 58 (48 - 68) | 74 (65 - 83) | 63 (55 - 73) | 58 (52 - 65) | 55 (46 - 63) | 47 (0 - 57) |
| Any use of inotropes, n (%) | 69 (2.4) | 2 (0.4) | 2 (0.4) | 15 (4.3) | 22 (2.5) | 24 (4.2) |
| Any use of vasopressors, n (%) | 1578 (56.6) | 19 (3.9) | 91 (18.2) | 227 (64.5) | 665 (75.5) | 538 (92.9) |
| Median highest dose of norepinephrine (IQR) - µg/kg/min | 0.21 (0.10 - 0.40) | 0.00 (0.00 - 0.11) | 0.08 (0.04 - 0.15) | 0.18 (0.08 - 0.35) | 0.20 (0.10 - 0.40) | 0.28 (0.15 - 0.50) |
| Median SOFA score (IQR) | 8 (5 - 10) | 4 (3 - 4) | 5 (5 - 6) | 7 (7 - 7) | 9 (8 - 9) | 12 (11 - 13) |

Baseline characteristics of the SOFA score-based subgroups. SOFA score denotes Sequential Organ Failure Assessment score, IQR interquartile range, ARDS acute respiratory distress syndrome, CPAP continuous positive airway pressure, PaO_2_ arterial partial pressure of oxygen, SaO_2_ arterial oxygen saturation, FiO_2_ fraction of inspired oxygen, n the number of patients in each group.

^a^ The aggregated SOFA score ranges from 0-24, with sub-score from 0-4 for 6 organ systems (respiration, coagulation, liver, cardiovascular, central nervous system, and renal), with higher scores indication higher degrees of organ failure. In total, 89 of 2888 patients had one or more missing SOFA sub-scores.

^b^ Data on all patients are included. Totals from SOFA score-based subgroups may not correspond to values in this column.

## Table S1b Baseline characteristics in the SOFA score-based subgroups stratified by treatment

| SOFA score range^a^ | All patients^b^ n = 2888 | SOFA score: 0-4 | | SOFA score: 5-6 | | SOFA score: 7-7 | | SOFA score: 8-10 | | SOFA score: 11-19 | |
| --- | --- | --- | --- | --- | --- | --- | --- | --- | --- | --- | --- |
|  |  | **Lower target n = 235** | **Higher target n = 251** | **Lower target, n = 270** | **Higher target n = 231** | **Lower target n = 179** | **Higher target n = 173** | **Lower target n = 435** | **Higher target n = 446** | **Lower target n = 278** | **Higher target n = 301** |
| Median age (IQR) - years | 70 (61 - 77) | 71 (59 - 77) | 67 (59 -75) | 71 (63 - 78) | 71 (62 - 78) | 71 (63 -78) | 70 (59 - 78) | 70 (62 - 77) | 70 (60 - 78) | 67 (56 - 74) | 69 (61 - 75) |
| Male sex, n (%) | 1855 (64.2) | 146 (62.1) | 159 (63.4) | 177 (65.6) | 148 (64.1) | 110 (61.5) | 112 (64.7) | 277 (63.7) | 290 (65.0) | 180 (64.8) | 202 (67.1) |
| Median interval from hospital admission to randomisation (IQR) - days | 1 (0 - 5) | 1 (0 - 4) | 2 (0 - 5) | 2 (1 - 5) | 2 (1 - 6) | 1 (0 - 5) | 1 (0 - 3) | 1 (0 - 5) | 1 (0 - 4) | 1 (0 - 5) | 1 (0 - 4) |
| Median interval from ICU admission to randomisation (IQR) - hours | 4 (2 - 7) | 3 (1 - 5) | 2 (1 - 5) | 3 (1 - 6) | 3 (1 -7) | 3 (2 - 8) | 4 (2 -7) | 4 (2 - 8) | 4 (2 -8) | 4 (2 - 8) | 4 (2 - 8) |
| Chronic co-morbidities, n (%) |  |  |  |  |  |  |  |  |  |  |  |
| Ischaemic heart disease | 409 (16.2) | 39 (16.6) | 35 (13.9) | 36 (13.3) | 26 (11.3) | 31 (17.3) | 22 (12.7) | 57 (13.1) | 60 (13.5) | 35 (12.6) | 53 (17.6) |
| Chronic heart failure | 285 (9.9) | 27 (11.5) | 26 (10.4) | 27 (10.0) | 28 (12.1) | 18 (10.1) | 17 (9.8) | 40 (9.2) | 33 (7.4) | 23 (8.3) | 36 (12.0) |
| Active metastatic cancer | 126 (4.4) | 11 (4.7) | 9 (3.6) | 10 (3.7) | 10 (4.3) | 10 (5.6) | 6 (3.5) | 23 (5.3) | 22 (4.9) | 11 (4.0) | 9 (3.0) |
| Chronic dialysis | 47 (1.6) | 4 (1.7) | 3 (1.2) | 0 (0.0) | 3 (1.3) | 2 (1.1) | 2 (1.2) | 8 (1.8) | 9 (2.0) | 5 (1.8) | 11 (3.7) |
| Chronic obstructive  pulmonary disease | 562 (19.5) | 50 (21.3) | 51 (20.3) | 58 (21.5) | 53 (22.9) | 47 (26.3) | 25 (20.8) | 79 (18.2) | 85 (19.1) | 35 (12.6) | 49 (16.3) |
| Active haematological   cancer | 167 (5.8) | 3 (1.3) | 10 (4.0) | 12 (4.4) | 21 (9.1) | 5 (2.8) | 7 (4.1) | 29 (6.7) | 25 (5.6) | 31 (11.2) | 21 (7.0) |
| Type of admission, n (%) |  |  |  |  |  |  |  |  |  |  |  |
| Medical | 2471 (85.6) | 215 (91.5) | 231 (92.0) | 242 (89.6) | 208 (90.0) | 135 (75.4) | 141 (81.5) | 358 (82.3) | 367 (82.3) | 248 (89.2) | 248 (82.4) |
| Elective surgical | 39 (1.4) | 5 (2.1) | 3 (1.2) | 0 (0.0) | 5 (2.2) | 4 (2.2) | 1 (0.6) | 8 (1.8) | 5 (1.1) | 1 (0.4) | 3 (1.0) |
| Emergency surgical | 378 (13.1) | 15 (6.4) | 17 (6.8) | 28 (10.4) | 18 (7.8) | 40 (22.4) | 31 (17.9) | 69 (15.9) | 74 (16.6) | 29 (10.4) | 50 (16.6) |
| Acute illness, n (%) |  |  |  |  |  |  |  |  |  |  |  |
| Pneumonia | 1664 (57.6) | 146 (62.1) | 163 (64.9) | 176 (65.2) | 154 (66.7) | 99 (55.3) | 82 (47.4) | 246 (56.6) | 248 (55.6) | 137 (49.3) | 158 (52.5) |
| Multiple trauma | 52 (1.8) | 3 (1.3) | 5 (2.0) | 4 (1.5) | 9 (3.9) | 4 (2.2) | 4 (2.3) | 8 (1.8) | 7 (1.6) | 2 (0.7) | 2 (0.7) |
| Haemorrhagic or ischaemic   stroke | 47 (1.6) | 1 (0.4) | 1 (0.4) | 4 (1.7) | 2 (0.7) | 3 (1.7) | 4 (2.3) | 12 (2.8) | 5 (1.1) | 5 (1.8) | 8 (2.7) |
| Traumatic brain injury | 22 (0.8) | 1 (0.4) | 2 (0.8) | 2 (0.7) | 3 (1.3) | 0 (0.0) | 1 (0.6) | 1 (0.2) | 3 (0.7) | 4 (1.4) | 4 (1.3) |
| Myocardial infarction | 182 (6.3) | 14 (6.0) | 14 (5.6) | 12 (4.4) | 12 (5.2) | 13 (7.3) | 14 (8.1) | 26 (6.0) | 27 (6.1) | 18 (6.5) | 28 (9.3) |
| Intestinal ischaemia | 68 (2.4) | 0 (0.0) | 5 (2.0) | 4 (1.5) | 5 (2.2) | 3 (1.7) | 6 (3.5) | 13 (3.0) | 16 (3.6) | 7 (2.5) | 9 (3.0) |
| Cardiac arrest | 332 (11.5) | 0 (0.0) | 0 (0.0) | 3 (1.1) | 3 (1.3) | 4 (2.3) | 6 (3.5) | 45 (10.3) | 59 (13.2) | 89 (32.0) | 112 (37.2) |
| ARDS | 366 (12.7) | 15 (6.4) | 39 (26.6) | 32 (11.9) | 28 (12.1) | 18 (10.1) | 15 (8.7) | 64 (14.7) | 56 (12.6) | 44 (15.8) | 51 (16.9) |
| Closed system, n (%) | 2062 (71.4) | 107 (45.5) | 137 (54.6) | 148 (54.8) | 119 (51.5) | 135 (75.4) | 130 (75.1) | 535 (81.2) | 347 (77.8) | 251 (90.3) | 278 (92.4) |
| Invasive mechanical   ventilation, n (%) | 1689 (58.5) | 48 (20.4) | 71 (28.3) | 82 (30.4) | 75 (32.5) | 114 (63.7) | 114 (65.9) | 313 (72.0) | 315 (70.6) | 244 (87.8) | 268 (89.0) |
| Median tidal volume   (IQR) – ml | 499 (429 - 574) | 502 (439 - 584) | 501 (421 - 634) | 489 (424 - 537) | 481 (416 - 543) | 503 (440 - 573) | 502 (419 - 600) | 492 (412- 576) | 491 (422 - 569) | 501 (436 - 553) | 513 (452 - 595) |
| Median end expiratory   pressure (IQR) - cm H_2_O | 9 (7 - 10) | 8 (6 - 10) | 8 (5 - 10) | 10 (8 - 12) | 8 (6 - 10) | 10 (8 - 10) | 8 (7 - 10) | 10 (7 - 12) | 9 (7 - 10) | 10 (7 - 10) | 9 (7 - 10) |
| Median peak inspiratory   pressure (IQR) - cm H_2_O | 25 (21 - 29) | 24 (21 - 28) | 24 (20 - 30) | 25 (21 - 30) | 22 (20 - 27) | 24 (21 - 28) | 25 (20 - 28) | 25 (20 - 29) | 25 (21 - 28) | 26 (22 - 30) | 25 (21 - 30) |
| Non-invasive ventilation or   CPAP, n (%) | 373 (12.9) | 59 (25.1) | 66 (26.3) | 66 (24.4) | 44 (19.1) | 21 (11.7) | 16 (9.3) | 40 (9.2) | 32 (7.2) | 7 (2.5) | 10 (3.3) |
| Median end expiratory   pressure (IQR) - cm H_2_O | 7 (5 - 9) | 6 (5 - 8) | 7 (5 - 8) | 8 (6 - 10) | 8 (7 - 10) | 8 (6 - 10) | 8 (6 - 8) | 7 (5 - 8) | 8 (6 - 9) | 8 (6 - 8) | 7 (5 - 10) |
| Open system, n (%) | 826 (28.6) | 128 (54.5) | 114 (45.4) | 122 (45.2) | 112 (48.5) | 44 (24.6) | 43 (24.9) | 80 (18.9) | 99 (22.2) | 27 (9.7) | 23 (7.6) |
| Median PaO_2_ (IQR) - kPa | 10.3 (8.7 - 12.5) | 10.4 (8.7 - 12.7) | 10.3 (8.7 - 13.4) | 9.9 (8.5- 12.0) | 9.7 (8.2 - 11.8) | 11.3 (9.4 - 13.5) | 10.5 (8.9 - 12.4) | 9.8 (8.7 - 12.2) | 10.4 (8.9 - 12.4) | 10.5 (8.9 - 12.7) | 10.5 (9.0 - 12.6) |
| Median SaO_2_ (IQR) - % | 94 (91 - 97) | 95 (93 - 97) | 95 (92 - 98) | 94 (91 - 97) | 94 (91 - 97) | 96 (93 - 97) | 95 (92 - 97) | 94 (91- 96) | 95 (92 - 97) | 94 (91 - 97) | 95 (90 - 97) |
| Median FiO_2_ (IQR) - fraction | 0.70 (0.55 - 0.85) | 0.60 (0.50 - 0.75) | 0.60 (0.50 - 0.70) | 0.70 (0.54 - 0.85) | 0.70 (0.54 - 0.90) | 0.65 (0.55 - 0.80) | 0.60 (0.55 - 0.80) | 0.70 (0.59 - 0.92) | 0.70 (0.60 - 0.90) | 0.75 (0.60 - 0.95) | 0.70 (0.60 - 0.90) |
| Median PaO_2_/FiO_2_ ratio (IQR) |  |  |  |  |  |  |  |  |  |  |  |
| In all systems | 15.7 (11.9 - 20.8) | 16.8 (14.0 - 22.2) | 17.1 (13.9 - 22.6) | 15.0 (11.1 - 21.4) | 14.5 (11.0 - 20.0) | 17.5 (14.0 - 22.6) | 17.0 (14.0 - 21.0) | 14.8 (10.8 - 19.9) | 15.2 (11.6 - 20.1) | 15.2 (11.4 - 19.8) | 14.9 (11.7 - 20.3) |
| In closed systems | 16.5 (12.4 - 21.6) | 19.0 (15.6 - 27.5) | 18.2 (15.0 - 26.5) | 16.5 (12.3 - 23.0) | 16.8 (12.5 - 24.5) | 18.0 (14.7 - 23.6) | 17.6 (15.1 - 21.7) | 15.3 (10.9 - 20.7) | 15.8 (11.9 - 20.6) | 15.5 (11.6 - 20.2) | 15.2 (11.8 - 20.4) |
| Median lactate concentration (IQR) - mM | 1.8 (1.1 - 3.2) | 1.3 (0.9 - 2.1) | 1.3 (0.9 - 1.9) | 1.3 (0.9 - 2.4) | 1.3 (0.9 - 2.1) | 2.0 (1.3 - 3.4) | 1.7 (1.2 - 2.8) | 1.8 (1.2 - 3.5) | 2.0 (1.3 - 3.5) | 2.8 (1.7 - 5.4) | 2.8 (1.6 - 5.6) |
| Median lowest mean arterial pressure (IQR) - mmHg | 58 (48 - 68) | 73 (65 - 81) | 74 (65 - 85) | 63 (55 - 73) | 64 (55 - 74) | 59 (52 - 65) | 58 (51 - 65) | 55 (46 - 63) | 55 (45 - 62) | 48 (0 - 59) | 47 (0 - 56) |
| Any use of inotropes, n (%) | 69 (2.4) | 2 (0.9) | 0 (0.0) | 1 (0.4) | 1 (0.4) | 5 (2.8) | 10 (5.8) | 8 (1.8) | 14 (3.1) | 13 (4.7) | 11 (3.7) |
| Any use of vasopressors, n (%) | 1578 (56.6) | 8 (3.4) | 11 (4.4) | 50 (18.5) | 41 (17.8) | 118 (65.9) | 109 (63.0) | 336 (77.2) | 329 (73.8) | 259 (93.2) | 279 (92.7) |
| Median highest dose of norepinephrine (IQR) - µg/kg/min | 0.21 (0.10 - 0.40) | 0.02 (0.00 - 0.16) | 0.00 (0.00 - 0.08) | 0.08 (0.05 - 0.20) | 0.07 (0.03 - 0.09) | 0.18 (0.08 - 0.35) | 0.18 (0.09 - 0.35) | 0.20 (0.10 - 0.39) | 0.20 (0.10 - 0.40) | 0.27 (0.15 - 0.50) | 0.28 (0.16 - 0.55) |
| Median SOFA score (IQR) | 8 (5 - 10) | 4 (3 - 4) | 4 (3 - 4) | 6 (5 - 6) | 5 (5 - 6) | 7 (7 - 7) | 7 (7 - 7) | 9 (8 - 9) | 9 (8 - 9) | 12 (11 - 13) | 12 (11 - 13) |

Baseline characteristics of the SOFA score-based subgroups. SOFA score denotes Sequential Organ Failure Assessment score, IQR interquartile range, ARDS acute respiratory distress syndrome, CPAP continuous positive airway pressure, PaO_2_ arterial partial pressure of oxygen, SaO_2_ arterial oxygen saturation, FiO_2_ fraction of inspired oxygen, n the number of patients in each group.

^a^ The aggregated SOFA score ranges from 0-24, with sub-score from 0-4 for 6 organ systems (respiration, coagulation, liver, cardiovascular, central nervous system, and renal), with higher scores indication higher degrees of organ failure. In total, 89 of 2888 patients had one or more missing SOFA sub-scores.

^b^ Data on all patients are included. Totals from SOFA score-based subgroups may not correspond to values in this column.

## Table S2a Baseline characteristics in the lactate concentration-based subgroups

| Plasma lactate concentration range - mM^a^ | All patients^b^ n = 2888 | Lactate: 0.2-0.9  n = 501 | Lactate: 1.0-1.4 n = 631 | Lactate: 1.5-2.1 n = 577 | Lactate: 2.2-3.6 n = 576 | Lactate: 3.7-24.0 n = 584 |
| --- | --- | --- | --- | --- | --- | --- |
| Median age (IQR) - years | 70 (61 - 77) | 68 (59 - 76) | 70 (60 - 77) | 71 (61 - 78) | 70 (61 - 76) | 70 (61 - 77) |
| Male sex, n (%) | 1855 (64.2) | 316 (63.1) | 404 (64.0) | 384 (66.6) | 371 (64.4) | 367 (62.8) |
| Median interval from hospital admission to randomisation (IQR) - days | 1 (0 - 5) | 2 (1 - 6) | 2 (1 - 5) | 1 (0 - 6) | 1 (0 - 4) | 1 (0 - 3) |
| Median interval from ICU admission to randomisation (IQR) - hours | 4 (2 - 7) | 4 (2 - 8) | 4 (2 - 7) | 3 (2 - 8) | 4 (2 - 7) | 3 (2 - 7) |
| Chronic co-morbidities, n (%) |  |  |  |  |  |  |
| Ischaemic heart disease | 409 (16.2) | 73 (14.6) | 88 (14.0) | 74 (12.8) | 93 (16.2) | 80 (13.7) |
| Chronic heart failure | 285 (9.9) | 55 (11.0) | 67 (10.6) | 54 (9.4) | 53 (9.2) | 55 (9.4) |
| Active metastatic cancer | 126 (4.4) | 16 (3.2) | 27 (4.3) | 25 (4.3) | 13 (5.4) | 26 (4.5) |
| Chronic dialysis | 47 (1.6) | 12 (2.4) | 10 (1.6) | 9 (1.6) | 6 (1.0) | 10 (1.7) |
| Chronic obstructive  pulmonary disease | 562 (19.5) | 120 (24.0) | 130 (20.6) | 113 (19.6) | 111 (19.3) | 87 (14.9) |
| Active haematological   cancer | 167 (5.8) | 24 (4.8) | 39 (6.2) | 33 (5.7) | 28 (4.9) | 42 (7.2) |
| Type of admission, n (%) |  |  |  |  |  |  |
| Medical | 2471 (85.6) | 460 (91.8) | 560 (88.8) | 497 (86.1) | 471 (81.8) | 467 (80.0) |
| Elective surgical | 39 (1.4) | 6 (1.2) | 8 (1.3) | 11 (1.9) | 7 (1.2) | 6 (1.0) |
| Emergency surgical | 378 (13.1) | 35 (7.0) | 63 (10.0) | 59 (12.0) | 98 (17.0) | 111 (19.0) |
| Acute illness, n (%) |  |  |  |  |  |  |
| Pneumonia | 1664 (57.6) | 328 (65.5) | 407 (64.5) | 355 (61.5) | 323 (56.1) | 244 (41.8) |
| Multiple trauma | 52 (1.8) | 8 (1.6) | 14 (2.2) | 12 (2.1) | 9 (1.6) | 5 (0.9) |
| Haemorrhagic or ischaemic   stroke | 47 (1.6) | 10 (2.0) | 10 (1.6) | 6 (1.0) | 9 (1.6) | 11 (1.9) |
| Traumatic brain injury | 22 (0.8) | 7 (1.4) | 4 (0.6) | 2 (0.4) | 7 (1.2) | 1 (0.2) |
| Myocardial infarction | 182 (6.3) | 15 (3.0) | 26 (4.1) | 37 (6.4) | 59 (10.2) | 45 (7.7) |
| Intestinal ischaemia | 68 (2.4) | 6 (1.2) | 7 (1.1) | 8 (1.4) | 14 (2.4) | 33 (5.7) |
| Cardiac arrest | 332 (11.5) | 18 (3.6) | 38 (6.0) | 58 (10.1) | 72 (12.5) | 145 (24.8) |
| ARDS | 366 (12.7) | 50 (14.0) | 86 (13.6) | 80 (13.9) | 70 (12.2) | 59 (10.1) |
| Closed system, n (%) | 2062 (71.4) | 328 (65.5) | 414 (65.6) | 398 (69.0) | 424 (73.6) | 484 (82.9) |
| Invasive mechanical   ventilation, n (%) | 1689 (58.5) | 233 (46.5) | 328 (52.0) | 324 (56.2) | 356 (61.8) | 439 (75.2) |
| Median tidal volume   (IQR) – ml | 499 (429 - 574) | 479 (422 - 550) | 484 (426 - 588) | 503 (417 - 582) | 500 (428 - 569) | 507 (441 - 586) |
| Median end expiratory   pressure (IQR) - cm H_2_O | 9 (7 - 10) | 9 (7 - 10) | 10 (7 - 10) | 9 (7 - 10) | 9 (8 - 10) | 8 (7 - 10) |
| Median peak inspiratory   pressure (IQR) - cm H_2_O | 25 (21 - 29) | 25 (20 - 30) | 25 (21 - 29) | 25 (21 - 29) | 25 (21 - 29) | 25 (21 - 29) |
| Non-invasive ventilation or   CPAP, n (%) | 373 (12.9) | 95 (19.0) | 89 (13.6) | 74 (12.8) | 68 (11.8) | 45 (7.7) |
| Median end expiratory   pressure (IQR) - cm H_2_O | 7 (5 - 9) | 5 (5 - 9) | 8 (5 - 10) | 7 (5 - 9) | 7 (5 - 8) | 7 (6 - 8) |
| Open system, n (%) | 826 (28.6) | 173 (34.5) | 217 (34.4) | 179 (31.0) | 152 (26.4) | 100 (17.1) |
| Median PaO_2_ (IQR) - kPa | 10.3 (8.7 - 12.5) | 10.3 (8.9 - 12.6) | 10.1 (8.6 - 12.1) | 10.2 (8.7 - 12.1) | 10.2 (8.7 - 12.4) | 10.6 (8.9 - 13.4) |
| Median SaO_2_ (IQR) - % | 94 (91 - 97) | 95 (92 - 97) | 94 (92 - 97) | 95 (92 - 97) | 94 (91 - 97) | 94 (90 - 97) |
| Median FiO_2_ (IQR) - fraction | 0.70 (0.55 - 0.85) | 0.65 (0.54 - 0.80) | 0.65 (0.55 - 0.80) | 0.65 (0.58 - 0.80) | 0.70 (0.60 - 0.90) | 0.75 (0.60 - 0.96) |
| Median PaO_2_/FiO_2_ ratio (IQR) |  |  |  |  |  |  |
| In all systems | 15.7 (11.9 - 20.8) | 16.6 (12.6 - 21.4) | 16.0 (11.8 - 20.5) | 15.8 (12.1 - 20.2) | 15.3 (11.5 - 21.0) | 15.3 (11.4 - 21.0) |
| In closed systems | 16.5 (12.4 - 21.6) | 18.0 (13.5 - 22.6) | 16.7 (12.8 - 21.0) | 16.5 (12.8 - 21.2) | 15.7 (12.0 - 21.6) | 15.7 |
| Median lactate concentration (IQR) - mM | 1.8 (1.1 - 3.2) | 0.8 (0.7 - 0.9) | 1.2 (1.1 - 1.3) | 1.7 (1.6 - 1.9) | 2.8 (2.4 - 3.1) | 5.8 (4.5 - 8.6) |
| Median lowest mean arterial pressure (IQR) - mmHg | 58 (48 - 68) | 64 (54 - 74) | 61 (53 - 73) | 59 (48 - 66) | 57 (48 - 66) | 50 (0 - 58) |
| Any use of inotropes, n (%) | 69 (2.4) | 3 (0.6) | 8 (1.3) | 15 (2.6) | 22 (3.8) | 21 (3.6) |
| Any use of vasopressors, n (%) | 1578 (56.6) | 186 (37.1) | 306 (48.5) | 284 (49.2) | 359 (62.3) | 434 (74.3) |
| Median highest dose of norepinephrine (IQR) - µg/kg/min | 0.21 (0.10 - 0.40) | 0.15 (0.08 - 0.20) | 0.17 (0.09 - 0.29) | 0.18 (0.09 - 0.30) | 0.24 (0.10 - 0.44) | 0.36 (0.20 - 0.60) |
| Median SOFA score (IQR) | 8 (5 - 10) | 6 (4 - 8) | 7 (5 - 9) | 7 (5 - 9) | 8 (6 - 11) | 9 (7 - 12) |

Baseline characteristics of the plasma lactate concentration-based subgroups. SOFA score denotes Sequential Organ Failure Assessment score, IQR interquartile range, ARDS acute respiratory distress syndrome, CPAP continuous positive airway pressure, PaO_2_ arterial partial pressure of oxygen, SaO_2_ arterial oxygen saturation, FiO_2_ fraction of inspired oxygen, n the number of patients in each group.

^a^ In total, 19 of 2888 patients had a missing plasma lactate concentration at baseline.

^b^ Data on all patients are included. Totals from plasma lactate-based subgroups may not correspond to values in this column.

## Table S2b Baseline characteristics in the lactate concentration-based subgroups stratified by treatment

| Plasma lactate concentration range - mM^a^ | All patients^b^ n = 2888 | Lactate: 0.2-0.9 | | Lactate: 1.0-1.4 | | Lactate: 1.5-2.1 | | Lactate: 1.5-2.1 | | Lactate: 3.7-24.0 | |
| --- | --- | --- | --- | --- | --- | --- | --- | --- | --- | --- | --- |
|  |  | **Lower target n = 250** | **Higher target n = 251** | **Lower target n = 325** | **Higher target n = 306** | **Lower target n = 266** | **Higher target n = 311** | **Lower target n = 302** | **Higher target n = 274** | **Lower target n = 290** | **Higher target n = 294** |
| Median age (IQR) - years | 70 (61 - 77) | 69 (61 - 77) | 67 (59 - 75) | 69 (60 - 77) | 71 (61 - 78) | 70 (60 -77) | 71 (62 - 78) | 71 (60 - 77) | 69 (60 - 76) | 70 (62 - 77) | 70 (61 - 77) |
| Male sex, n (%) | 1855 (64.2) | 151 (60.4) | 165 (65.7) | 211 (64.9) | 193 (63.0) | 173 (65.0) | 211 (67.9) | 200 (66.2) | 171 (62.4) | 175 (60.3) | 192 (65.3) |
| Median interval from hospital admission to randomisation (IQR) - days | 1 (0 - 5) | 2 (1 - 5) | 2 (1 - 7) | 2 (1 - 6) | 2 (1 - 5) | 2 (1 - 6) | 1 (0 - 6) | 1 (0 - 6) | 1 (0 - 4) | 1 (0 - 3) | 1 (0 - 2) |
| Median interval from ICU admission to randomisation (IQR) - hours | 4 (2 - 7) | 4 (2 - 7) | 4 (2 - 8) | 4 (1 - 8) | 3 (2 - 7) | 4 (2 - 8) | 3 (2 - 7) | 4 (2 - 7) | 4 (2 - 7) | 3 (2 - 6) | 4 (2 - 7) |
| Chronic co-morbidities, n (%) |  |  |  |  |  |  |  |  |  |  |  |
| Ischaemic heart disease | 409 (16.2) | 37 (14.8) | 36 (14.3) | 44 (13.5) | 44 (14.4) | 37 (13.9) | 37 (11.9) | 47 (15.6) | 46 (16.8) | 39 (13.5) | 41 (14.0) |
| Chronic heart failure | 285 (9.9) | 24 (9.6) | 31 (12.4) | 39 (12.0) | 28 (9.2) | 25 (9.4) | 29 (9.3) | 25 (8.3) | 28 (10.2) | 26 (9.0) | 29 (9.9) |
| Active metastatic cancer | 126 (4.4) | 7 (2.8) | 9 (3.6) | 17 (5.2) | 10 (3.3) | 10 (3.8) | 15 (4.8) | 17 (5.6) | 14 (5.1) | 14 (4.8) | 12 (4.1) |
| Chronic dialysis | 47 (1.6) | 6 (2.4) | 6 (2.4) | 3 (0.9) | 7 (2.3) | 4 (1.5) | 5 (1.6) | 2 (0.7) | 4 (1.5) | 4 (1.4) | 6 (2.0) |
| Chronic obstructive  pulmonary disease | 562 (19.5) | 65 (26.0) | 55 (21.9) | 60 (18.5) | 70 (22.9) | 54 (20.3) | 59 (19.0) | 51 (17.2) | 59 (21.5) | 46 (15.9) | 41 (14.0) |
| Active haematological   cancer | 167 (5.8) | 10 (4.0) | 14 (5.6) | 20 (6.2) | 19 (6.2) | 15 (5.6) | 18 (5.8) | 15 (5.0) | 13 (4.7) | 21 (7.2) | 21 (7.1) |
| Type of admission, n (%) |  |  |  |  |  |  |  |  |  |  |  |
| Medical | 2471 (85.6) | 230 (92.0) | 230 (91.6) | 289 (88.9) | 271 (88.6) | 323 (87.2) | 265 (85.2) | 254 (84.1) | 217 (79.2) | 227 (78.3) | 240 (81.6) |
| Elective surgical | 39 (1.4) | 3 (1.02) | 3 (1.2) | 4 (1.2) | 4 (1.3) | 4 (1.5) | 7 (2.3) | 3 (1.0) | 4 (1.5) | 3 (1.0) | 3 (1.0) |
| Emergency surgical | 378 (13.1) | 17 (6.8) | 18 (7.2) | 32 (9.9) | 31 (10.1) | 30 (11.3) | 39 (12.5) | 45 (14.9) | 53 (19.3) | 60 (20.7) | 51 (17.4) |
| Acute illness, n (%) |  |  |  |  |  |  |  |  |  |  |  |
| Pneumonia | 1664 (57.6) | 172 (68.8) | 156 (62.2) | 207 (63.7) | 200 (65.4) | 161 (60.5) | 194 (62.4) | 173 (57.3) | 150 (54.7) | 118 (40.7) | 126 (42.9) |
| Multiple trauma | 52 (1.8) | 3 (1.2) | 5 (2.0) | 7 (2.2) | 7 (2.3) | 6 (2.3) | 6 (1.9) | 3 (1.0) | 6 (2.2) | 2 (0.7) | 3 (1.0) |
| Haemorrhagic or ischaemic   stroke | 47 (1.6) | 4 (1.6) | 6 (2.4) | 8 (2.5) | 2 (0.7) | 1 (0.4) | 5 (1.6) | 7 (2.3) | 2 (0.7) | 4 (1.4) | 7 (2.4) |
| Traumatic brain injury | 22 (0.8) | 3 (1.2) | 4 (1.6) | 2 (0.6) | 2 (0.7) | 1 (0.4) | 1 (0.3) | 1 (0.3) | 6 (2.2) | 1 (0.3) | 0 (0.0) |
| Myocardial infarction | 182 (6.3) | 6 (2.4) | 9 (3.6) | 12 (3.7) | 14 (4.6) | 15 (5.6) | 22 (4.6) | 30 (9.9) | 29 (10.6) | 21 (7.2) | 24 (8.2) |
| Intestinal ischaemia | 68 (2.4) | 1 (0.4) | 5 (2.0) | 1 (0.3) | 6 (2.0) | 2 (0.8) | 6 (1.9) | 5 (1.7) | 9 (3.3) | 18 (6.2) | 15 (5.1) |
| Cardiac arrest | 332 (11.5) | 13 (5.2) | 5 (2.0) | 10 (3.1) | 28 (9.2) | 21 (7.9) | 37 (11.9) | 40 (13.3) | 32 (11.7) | 63 (21.7 | 82 (27.9) |
| ARDS | 366 (12.7) | 29 (11.6) | 41 (16.3) | 40 (12.3) | 46 (15.0) | 36 (13.5) | 44 (14.2) | 39 (12.9) | 31 (11.3) | 30 (10.3) | 29 (9.9) |
| Closed system, n (%) | 2062 (71.4) | 167 (66.8) | 161 (64.1) | 215 (66.2) | 199 (65.0) | 185 (69.6) | 213 (68.5) | 215 (71.2) | 209 (76.3) | 238 (82.1) | 246 (83.7) |
| Invasive mechanical   ventilation, n (%) | 1689 (58.5) | 112 (44.8) | 121 (48.2) | 171 (52.6) | 157 (51.3) | 152 (57.1) | 172 (55.3) | 176 (58.3) | 180 (65.7) | 213 (73.5) | 226 (76.9) |
| Median tidal volume   (IQR) – ml | 499 (429 - 574) | 483 (435 - 578) | 471 (400 - 546) | 491 (429 - 558) | 480 (418 - 558) | 503 (433 - 569) | 505 (416 - 596) | 499 (408 - 552) | 502 (434 - 581) | 505 (429 - 566) | 512 (448 - 598) |
| Median end expiratory   pressure (IQR) - cm H_2_O | 9 (7 - 10) | 8 (7 -10) | 9 (7- 11) | 10 (7 - 12) | 10 (7 - 10) | 10 (8 - 11) | 8 (7 - 10) | 9 (8 -10) | 9 (7 - 10) | 8 (7 - 10) | 8 (7 - 10) |
| Median peak inspiratory   pressure (IQR) - cm H_2_O | 25 (21 - 29) | 24 (20 - 30) | 25 (20 - 30) | 26 (21 - 30) | 24 (20 - 28) | 26 (22 - 29) | 25 (20 - 28) | 25 (21 - 29) | 25 (21 - 30) | 25 (21 - 30) | 25 (21 - 29) |
| Non-invasive ventilation or   CPAP, n (%) | 373 (12.9) | 55 (22.0) | 40 (15.9) | 44 (13.5) | 42 (13.7) | 33 (12.4) | 41 (13.2) | 39 (12.9) | 29 (10.6) | 25 (8.6) | 20 (6.8) |
| Median end expiratory   pressure (IQR) - cm H_2_O | 7 (5 - 9) | 6 (5 - 8) | 8 (7 - 10) | 8 (5 - 10) | 8 (5 - 9) | 7 (6 - 8) | 7 (5 - 9) | 8 (6 - 9) | 7 (5 - 8) | 7 (6 - 8) | 8 (6 - 9) |
| Open system, n (%) | 826 (28.6) | 83 (33.2) | 90 (35.9) | 110 (33.9) | 107 (35.0) | 81 (30.5) | 98 (31.5) | 87 (28.8) | 65 (23.7) | 52 (17.9) | 48 (16.3) |
| Median PaO_2_ (IQR) - kPa | 10.3 (8.7 - 12.5) | 10.1 (9.0 - 12.4) | 10.3 (8.8 - 12.7) | 10.0 (8.5 - 12.0) | 10.1 (8.7 - 12.2) | 10.3 (8.9 - 12.3) | 10.0 (8.6 - 11.9) | 10.2 (8.6 - 12.6) | 10.2 (8.8 - 12.2) | 10.6 (8.9 - 14.0) | 10. 7 (9.0 - 12.8) |
| Median SaO_2_ (IQR) - % | 94 (91 - 97) | 95 (92 - 97) | 95 (92 - 97) | 94 (91 - 97) | 95 (92 - 97) | 95 (92 - 97) | 94 (91 - 96) | 94 (91 - 97) | 94 (91 - 97) | 94 (90 - 97) | 94 (90 - 97) |
| Median FiO_2_ (IQR) - fraction | 0.70 (0.55 - 0.85) | 0.62 (0.50) | 0.65 (0.55 - 0.80) | 0.65 (0.55 - 0.85) | 0.65 (0.55 - 0.80) | 0.65 (0.59 - 0.80) | 0.65 (0.55 - 0.80) | 0.70 (0.55 - 0.90) | 0.70 (0.55 - 0.85) | 0.75 (0.60 - 1.00) | 0.75 (0.60 - 0.95) |
| Median PaO_2_/FiO_2_ ratio (IQR) |  |  |  |  |  |  |  |  |  |  |  |
| In all systems | 15.7 (11.9 - 20.8) | 16.7 (12.5 - 21.6) | 16.2 (12.6 - 20.8) | 15.6 (12.0 - 20.6) | 16.2 (11.8 - 20.3) | 15.9 (12.1 - 20.2). | 15.5 (12.3 - 20.0) | 15.5 (11,5 - 21.2) | 15.2 (11.5 -20.5) | 15.6 (11.3 - 22.0) | 15.2 (11.7 - 20.5) |
| In closed systems | 16.5 (12.4 - 21.6) | 18.0 (13.5 - 22.3) | 17.9 (13.4 - 23.3) | 16.2 (12.5 - 21.0) | 17.1 (13.1) | 16.6 (13.0 - 21.5) | 16.3 (12.7 - 20.9) | 15.7 (11.6 - 21.8) | 15.8 (12.2 - 21.2) | 16.0 (11.4 - 22.3) | 15.6 (12.0 - 20.8) |
| Median lactate concentration (IQR) - mM | 1.8 (1.1 - 3.2) | 0.8 (0.6 - 0.8) | 0.8 (0.7 - 0.9) | 1.2 (1.1 - 1.3) | 1.2 (1.1 - 1.3) | 1.8 (1.6 - 1.9) | 1.7 (1.6 - 1.9) | 2.8 (2.4 - 3.2) | 2.8 (2.4 - 3.1) | 5.9 (4.6 - 9.0) | 5.8 (4.3 - 8.3) |
| Median lowest mean arterial pressure (IQR) - mmHg | 58 (48 - 68) | 64 ( 53 - 73) | 64 (55 - 75) | 53 (55 - 73) | 60 (52 - 73) | 59 (50 - 68) | 60 (50 - 70) | 57 (47 - 66) | 57 (48 - 67) | 50 (32 - 57) | 49 (0 - 58) |
| Any use of inotropes, n (%) | 69 (2.4) | 1 (0.4) | 2 (0.8) | 3 (0.9) | 5 (1.6) | 5 (1.9) | 10 (3.2) | 11 (3.6) | 11 (4.0) | 12 (4.14) | 9 (3.1) |
| Any use of vasopressors, n (%) | 1578 (56.6) | 95 (38.0) | 91 (36.3) | 148 (45.5) | 158 (51.6) | 138 (51.9) | 146 (47.0) | 192 (63.6) | 167 (61.0) | 218 (75.2) | 216 (73.5) |
| Median highest dose of norepinephrine (IQR) - µg/kg/min | 0.21 (0.10 - 0.40) | 0.15 (0.08 - 0.21) | 0.14 (0.07 - 0.20) | 0.15 (0.09 - 0.26) | 0.18 (0.08 - 0.30) | 0.17 (0.09 - 0.30) | 0.19 (0.09 - 0.35) | 0.22 (0.10 - 0.43) | 0.25 (0.11 - 0.45) | 0.35 (0.21 - 0.61) | 0.38 (0.20 - 0.60) |
| Median SOFA score (IQR) | 8 (5 - 10) | 6 (4 - 8) | 6 (4 - 8) | 7 (5 - 9) | 7 (5 - 9) | 8 (6 - 9) | 7 (5 - 9) | 8 (6 - 11) | 8 (6 - 10) | 9 (7 - 11) | 10 (8 - 12) |

Baseline characteristics of the plasma lactate concentration-based subgroups. SOFA score denotes Sequential Organ Failure Assessment score, IQR interquartile range, ARDS acute respiratory distress syndrome, CPAP continuous positive airway pressure, PaO_2_ arterial partial pressure of oxygen, SaO_2_ arterial oxygen saturation, FiO_2_ fraction of inspired oxygen, n the number of patients in each group.

^a^ In total, 19 of 2888 patients had a missing plasma lactate concentration at baseline.

^b^ Data on all patients are included. Totals from plasma lactate-based subgroups may not correspond to values in this column.

## Table S3a Baseline characteristics in the norepinephrine dose-based subgroups

| Norepinephrine dose range - µg/kg/min^a^ | All patients^b^ n = 2888 | Norepinephrine dose: 0.00-0.00 n = 1373 | Norepinephrine dose: 0.01-0.10 n = 366 | Norepinephrine dose: 0.11-0.21 n = 372 | Norepinephrine dose: 0.22-0.39 n = 348 | Norepinephrine dose: 0.40-2.40 n = 429 |
| --- | --- | --- | --- | --- | --- | --- |
| Median age (IQR) - years | 70 (61 - 77) | 69 (59 - 77) | 71 (62 - 78) | 70 (61 - 76) | 71 (62 - 77) | 70 (62 - 76) |
| Male sex, n (%) | 1855 (64.2) | 871 (63.4) | 238 (65.0) | 249 (67.0) | 218 (62.6) | 279 (65.0) |
| Median interval from hospital admission to randomisation (IQR) - days | 1 (0 - 5) | 2 (0 - 5) | 1 (0 - 5) | 1 (0 - 5) | 1 (0 - 5) | 1 (0 - 3) |
| Median interval from ICU admission to randomisation (IQR) - hours | 4 (2 - 7) | 3 (1 - 6) | 4 (2 - 8) | 4 (2 - 8) | 4 (2 - 8) | 5 (3 - 9) |
| Chronic co-morbidities, n (%) |  |  |  |  |  |  |
| Ischaemic heart disease | 409 (16.2) | 193 (14.1) | 52 (14.2) | 51 (13.7) | 49 (14.1) | 64 (14.9) |
| Chronic heart failure | 285 (9.9) | 156 (11.4) | 29 (7.9) | 32 (8.6) | 28 (8.1) | 40 (9.3) |
| Active metastatic cancer | 126 (4.4) | 51 (3.7) | 14 (3.8) | 18 (4.8) | 25 (7.2) | 18 (4.2) |
| Chronic dialysis | 47 (1.6) | 14 (1.0) | 3 (0.8) | 8 (2.2) | 9 (2.6) | 13 (3.0) |
| Chronic obstructive  pulmonary disease | 562 (19.5) | 280 (20.4) | 68 (18.6) | 69 (18.6) | 72 (20.7) | 73 (17.0) |
| Active haematological   cancer | 167 (5.8) | 85 (6.2) | 15 (4.1) | 19 (5.1) | 19 (5.5) | 29 (6.8) |
| Type of admission, n (%) |  |  |  |  |  |  |
| Medical | 2471 (85.6) | 1276 (92.9) | 292 (79.8) | 309 (83.1) | 275 (79.0) | 319 (74.4) |
| Elective surgical | 39 (1.4) | 15 (1.1) | 6 (1.6) | 7 (1.9) | 7 (2.0) | 4 (0.9) |
| Emergency surgical | 378 (13.1) | 82 (6.0) | 68 (18.6) | 56 (15.1) | 66 (19.0) | 106 (24.7) |
| Acute illness, n (%) |  |  |  |  |  |  |
| Pneumonia | 1664 (57.6) | 854 (62.2) | 188 (51.4) | 223 (60.0) | 192 (55.2) | 207 (48.3) |
| Multiple trauma | 52 (1.8) | 29 (2.1) | 6 (1.6) | 7 (1.9) | 3 (0.9) | 7 (1.6) |
| Haemorrhagic or ischaemic   stroke | 47 (1.6) | 17 (1.2) | 8 (2.2) | 10 (2.7) | 6 (1.7) | 6 (1.4) |
| Traumatic brain injury | 22 (0.8) | 11 (0.8) | 2 (0.6) | 6 (1.6) | 2 (0.6) | 2 (0.6) |
| Myocardial infarction | 182 (6.3) | 73 (5.3) | 28 (7.7) | 27 (7.3) | 25 (7.2) | 29 (6.8) |
| Intestinal ischaemia | 68 (2.4) | 14 (1.0) | 9 (2.5) | 2 (0.5) | 10 (2.9) | 33 (7.7) |
| Cardiac arrest | 332 (11.5) | 98 (7.1) | 52 (14.2) | 47 (12.6) | 50 (14.37) | 85 (19.8) |
| ARDS | 366 (12.7) | 151 (11.0) | 55 (15.0) | 51 (13.7) | 58 (16.7) | 51 (11.9) |
| Closed system, n (%) | 2062 (71.4) | 761 (55.4) | 295 (80.6) | 309 (83.1) | 300 (86.2) | 397 (92.5) |
| Invasive mechanical   ventilation, n (%) | 1689 (58.5) | 450 (32.8) | 277 (75.7) | 289 (77.7) | 285 (81.9) | 388 (90.4) |
| Median tidal volume   (IQR) – ml | 499 (429 - 574) | 469 (425 - 570) | 500 (430 - 570) | 500 (426 - 570) | 496 (428 - 588) | 501 (436 - 578) |
| Median end expiratory   pressure (IQR) - cm H_2_O | 9 (7 - 10) | 8 (7 - 10) | 8 (6 - 10) | 10 (8 - 10) | 10 (8 - 10) | 10 (7 - 11) |
| Median peak inspiratory   pressure (IQR) - cm H_2_O | 25 (21 - 29) | 24 (20 - 29) | 25 (20 - 29) | 25 (21 - 29) | 25 (21 - 29) | 25 (21 - 29) |
| Non-invasive ventilation or   CPAP, n (%) | 373 (12.9) | 311 (22.7) | 18 (4.9) | 20 (5.4) | 15 (4.3) | 9 (2.1) |
| Median end expiratory   pressure (IQR) - cm H_2_O | 7 (5 - 9) | 7 (5 - 9) | 8 (7 - 8) | 8 (6 - 10) | 7 (5 - 8) | 8 (8 - 10) |
| Open system, n (%) | 826 (28.6) | 612 (44.6) | 71 (19.4) | 63 (16.9) | 48 (13.8) | 32 (7.5) |
| Median PaO_2_ (IQR) - kPa | 10.3 (8.7 - 12.5) | 9.8 (8.5 - 11.7) | 10.6 (9.1 - 13.3) | 10.6 (8.9 - 13.6) | 10.6 (9.2 - 13.0) | 10.6 (9.0 - 12.8) |
| Median SaO_2_ (IQR) - % | 94 (91 - 97) | 94 (91 - 96) | 95 (93 - 97) | 95 (92 - 97) | 95 (91 -97) | 94 (91 - 97) |
| Median FiO_2_ (IQR) - fraction | 0.70 (0.55 - 0.85) | 0.65 (0.55 - 0.80) | 0.62 (0.55 - 0.80) | 0.70 (0.55 - 0.85) | 0.70 (0.60 - 0.90) | 0.75 (0.60 - 0.90) |
| Median PaO_2_/FiO_2_ ratio (IQR) |  |  |  |  |  |  |
| In all systems | 15.7 (11.9 - 20.8) | 15.2 (11.6 - 20.0) | 16.8 (12.8 - 22.2) | 17.0 (13.0 - 21.6) | 15.8 (11.8 - 20.8) | 15.3 (11.5 - 20.7) |
| In closed systems | 16.5 (12.4 - 21.6) | 16.5 (12.7 - 21.4) | 17.6 (13.7 - 22.8) | 17.1 (13.4 - 21.8) | 16.0 (11.7 - 21.1) | 15.3 (11.6 - 20.7) |
| Median lactate concentration (IQR) - mM | 1.8 (1.1 - 3.2) | 1.5 (1.0 - 2.4) | 1.6 (1.1 - 2.5) | 1.6 (1.1 - 2.9) | 2.3 (1.4 - 4.3) | 3.5 (2.0 - 6.2) |
| Median lowest mean arterial pressure (IQR) - mmHg | 58 (48 - 68) | 65 (55 - 77) | 57 (48 - 62) | 55 (46 - 61) | 53 (43 - 60) | 49 (36 - 56) |
| Any use of inotropes, n (%) | 69 (2.4) | 13 (1.0) | 18 (4.9) | 7 (1.9) | 11.(3.2) | 20 (4.7) |
| Any use of vasopressors, n (%) | 1578 (56.6) | 63 (4.6) | 366 (100.0) | 372 (100.0) | 348 (100.0) | 429 (100.0) |
| Median highest dose of norepinephrine (IQR) - µg/kg/min | 0.21 (0.10 - 0.40) | 0.00 (0.00 - 0.00) | 0.07 (0.05 - 0.10) | 0.16 (0.14 - 0.20) | 0.30 (0.25 - 0.33) | 0.60 (0.48 - 0.75) |
| Median SOFA score (IQR) | 8 (5 - 10) | 5 (4 - 7) | 8 (7 - 10) | 9 (8 - 11) | 10 (8 - 12) | 10 (8 - 12) |

Baseline characteristics of the norepinephrine dose -based subgroups. SOFA score denotes Sequential Organ Failure Assessment score, IQR interquartile range, ARDS acute respiratory distress syndrome, CPAP continuous positive airway pressure, PaO_2_ arterial partial pressure of oxygen, SaO_2_ arterial oxygen saturation, FiO_2_ fraction of inspired oxygen, n the number of patients in each group.

^a^ No patients had missing data on norepinephrine dose at baseline. Due to the zero-inflated distribution of the parameter, the number of patients in the first subgroup (norepinephrine dose: 0.00 – 0.00 µg/kg/min) is larger than the other subgroups.

^b^ Data on all patients are included. Totals from norepinephrine dose-based subgroups may not correspond to values in this column.

## Table S3b Baseline characteristics in the norepinephrine dose-based subgroups stratified by treatment

| Norepinephrine dose range - µg/kg/min^a^ | All patients^b^ n = 2888 | Norepinephrine dose: 0.00-0.00 | | Norepinephrine dose: 0.01-0.10 | | Norepinephrine dose: 0.11-0.21 | | Norepinephrine dose:  0.22-0.39 | | Norepinephrine dose: 0.40-2.40 | |
| --- | --- | --- | --- | --- | --- | --- | --- | --- | --- | --- | --- |
|  |  | **Lower target n = 677** | **Higher target n = 696** | **Lower target**  **n = 192** | **Higher target n = 174** | **Lower target n = 185** | **Higher target n = 187** | **Lower target n = 181** | **Higher target n = 167** | **Lower target n = 206** | **Higher target n = 223** |
| Median age (IQR) - years | 70 (61 - 77) | 70 (60 - 77) | 68 (58 - 76) | 70 (61 - 77) | 71 (62 - 79) | 70 (61 - 76) | 69 (61 - 77) | 70 (62 - 76) | 72 (63 - 78) | 68 (62 - 76) | 70 (62 - 76) |
| Male sex, n (%) | 1855 (64.2) | 425 (62.78) | 446 (64.0) | 126 (65.6) | 112 (64.4) | 120 (64.9) | 129 (69.0) | 113 (62.4) | 105 (62.9) | 132 (64.08) | 147 (65.9) |
| Median interval from hospital admission to randomisation (IQR) - days | 1 (0 - 5) | 2 (1 - 5) | 1 (0 - 5) | 1 (0 - 6) | 1 (0 - 4) | 1 (0 - 5) | 1 (0 - 4) | 1 (0 - 5) | 1 (0- 5) | 1 (0 - 4) | 1 (0 - 3) |
| Median interval from ICU admission to randomisation (IQR) - hours | 4 (2 - 7) | 3 (1 - 6) | 3 (1 - 6) | 5 (2 - 8) | 4 (2 - 7) | 4 (2 - 8) | 4 (2 - 8) | 5 (2 - 8) | 4 (2 - 8) | 5 (3 - 8) | 6 (3 - 9) |
| Chronic co-morbidities, n (%) |  |  |  |  |  |  |  |  |  |  |  |
| Ischaemic heart disease | 409 (16.2) | 102 (15.1) | 91 (13.1) | 24 (12.5) | 28 (16.1) | 23 (12.4) | 28 (15.9) | 23 (12.7) | 26 (15.6) | 32 (15.5) | 32 (14.4) |
| Chronic heart failure | 285 (9.9) | 73 (10.8) | 83 (11.9) | 16 (8.3) | 13 (7.5) | 14 (7.6) | 18 (9.6) | 13 (7.2) | 15 (9.0) | 23 (11.2) | 17 (7.6) |
| Active metastatic cancer | 126 (4.4) | 24 (3.6) | 27 (3.9) | 7 (3.7) | 7 (4.0) | 10 (5.4) | 8 (4.3) | 17 (9.4) | 8 (4.8) | 7 (3.4) | 11 (4.9) |
| Chronic dialysis | 47 (1.6) | 5 (0.7) | 9 (1.3) | 1 (0.5) | 2 (1.2) | 7 (3.8) | 1 (0.5) | 3 (1.7) | 6 (3.6) | 3 (1.5) | 10 (4.5) |
| Chronic obstructive  pulmonary disease | 562 (19.5) | 142 (21.0) | 138 (19.8) | 37 (19.3) | 31 (17.8) | 32 (17.3) | 37 (19.8) | 32 (17.7) | 40 (24.0) | 34 (16.5) | 39 (17.5) |
| Active haematological   cancer | 167 (5.8) | 36 (5.3) | 49 (7.0) | 9 (4.7) | 6 (3.5) | 11 (6.0) | 8 (4.3) | 9 (5.0) | 10 (6.0) | 16 (7.8) | 13 (5.8) |
| Type of admission, n (%) |  |  |  |  |  |  |  |  |  |  |  |
| Medical | 2471 (85.6) | 630 (93.1) | 646 (92.8) | 149 (77.6) | 143 (82.2) | 155 (83.8) | 154 (82.4) | 145 (80.1) | 130 (77.8) | 159 (77.2) | 160 (71.8) |
| Elective surgical | 39 (1.4) | 6 (0.9) | 9 (1.3) | 5 (2.6) | 1 (0.6) | 2 (1.1) | 5 (2.7) | 3 (1.7) | 4 (2.4) | 2 (1.0) | 2 (0.9) |
| Emergency surgical | 378 (13.1) | 41 (6.1) | 41 (5.9) | 38 (19.8) | 30 (17.2) | 28 (15.1) | 28 (15.0) | 33 (18.2) | 33 (19.8) | 45 (21.8) | 61 (27.4) |
| Acute illness, n (%) |  |  |  |  |  |  |  |  |  |  |  |
| Pneumonia | 1664 (57.6) | 418 (61.7) | 436 (62.6) | 100 (52.1) | 88 (50.6) | 112 (60.5) | 111 (59.4) | 104 (57.5) | 88 (52.7) | 99 (48.1) | 108 (48.4) |
| Multiple trauma | 52 (1.8) | 11 (1.6) | 18 (2.6) | 4 (2.1) | 2 (1.2) | 5 (2.7) | 2 (1.2) | 1 (0.6) | 5 (2.2) | 2 (1.0) | 11 (1.62) |
| Haemorrhagic or ischaemic   stroke | 47 (1.6) | 10 (1.5) | 7 (1.0) | 5 (2.6) | 3 (1.7) | 5 (2.7) | 5 (2.7) | 1 (0.6) | 5 (3.0) | 4 (1.9) | 2 (0.9) |
| Traumatic brain injury | 22 (0.8) | 4 (0.6) | 7 (1.0) | 0 (0.0) | 2 (1.2) | 2 (1.1) | 4 (2.1) | 1 (0.6) | 1 (0.6) | 1 (0.5) | 0 (0.0) |
| Myocardial infarction | 182 (6.3) | 33 (4.9) | 40 (5.8) | 14 (7.3) | 14 (8.1) | 13 (7.0) | 14 (7.5) | 8 (4.4) | 17 (10.2) | 16 (7.8) | 13 (5.8) |
| Intestinal ischaemia | 68 (2.4) | 3 (0.4) | 11 (1.6) | 3 (1.6) | 6 (3.5) | 0 (0.0) | 2 (1.1) | 6 (3.3) | 4 (2.4) | 15 (7.3) | 18 (8.1) |
| Cardiac arrest | 332 (11.5) | 40 (5.9) | 58 (8.3) | 24 (12.5) | 28 (16.1) | 25 (13.5) | 22 (11.8) | 21 (11.6) | 29 (17.4) | 37 (18.0) | 48 (21.5) |
| ARDS | 366 (12.7) | 60 (8.9) | 91 (3.1) | 32 (16.7) | 23 (13.2) | 20 (10.8) | 31 (16.6) | 37 (20.4) | 21 (12.6) | 26 (12.6) | 25 (11.2) |
| Closed system, n (%) | 2062 (71.4) | 368 (54.4) | 393 (56.5) | 152 (79.2) | 143 (82.2) | 154 (83.2) | 155 (83.9) | 159 (87.9) | 141 (84.4) | 191 (92.7) | 105 (92.4) |
| Invasive mechanical   ventilation, n (%) | 1689 (58.5) | 202 (29.8) | 248 (35.6) | 144 (75.0) | 133 (76.4) | 142 (76.8) | 147 (78.6) | 152 (84.0) | 133 (79.6) | 186 (90.3) | 202 (90.6) |
| Median tidal volume   (IQR) – ml | 499 (429 - 574) | 496 (429 - 550) | 493 (423 - 594) | 495 (426 - 560) | 505 (437 - 570) | 501 (442 - 563) | 497 (415 - 573) | 491 (413 - 580) | 508 (448 - 590) | 505 (437 - 565) | 499 (434 - 594) |
| Median end expiratory   pressure (IQR) - cm H_2_O | 9 (7 - 10) | 8 (7 - 10) | 8 (7 - 10) | 8 (7 - 10) | 8 (5 - 10) | 9 (7 - 11) | 10 (8 - 10) | 10 (8 - 10) | 10 (7 - 10) | 10 (8 - 12) | 9 (7 - 10) |
| Median peak inspiratory   pressure (IQR) - cm H_2_O | 25 (21 - 29) | 24 (20 - 29) | 24 (20 - 28) | 25 (21 - 29) | 25 (20 - 30) | 25 (21 - 30) | 25 (21 - 29) | 25 (22 - 30) | 25 (21 - 28) | 26 (22 - 30) | 21 (21 - 29) |
| Non-invasive ventilation or   CPAP, n (%) | 373 (12.9) | 166 (24.5) | 145 (20.8) | 4 (4.2) | 10 (5.8) | 12 (6.5) | 8 (8.3) | 7 (3.9) | 8 (4.8) | 5 (2.4) | 4 (1.8) |
| Median end expiratory   pressure (IQR) - cm H_2_O | 7 (5 - 9) | 7 (5 - 8) | 8 (5 - 9) | 7 )6 - 8) | 8 (8 - 9) | 8 (6 - 9) | 8 (6 - 10) | 8 (7 - 10) | 6 (5 - 8) | 8 (8 - 9) | 9 (8 - 12) |
| Open system, n (%) | 826 (28.6) | 309 (45.6) | 303 (43.5) | 40 (20.8) | 31 (17.8) | 31 (16.8) | 32 (17.1) | 22 (12.2) | 26 (15.6) | 15 (7.3) | 17 (7.6) |
| Median PaO_2_ (IQR) - kPa | 10.3 (8.7 - 12.5) | 9.8 (8.5 - 11.7) | 9.8 (8.4 - 11.7) | 10.6 (9.1 - 13.1) | 10.7 (9.2 - 13.7) | 10.6 (8.8 - 14.3) | 10.7 (9.0 - 13.4) | 10.6 (9.2 - 13.3) | 10.6 (9.3 - 12.7) | 10.5 (8.9 - 12.9) | 10.6 (9.2 - 12.8) |
| Median SaO_2_ (IQR) - % | 94 (91 - 97) | 94 (91 - 96) | 94 (91 - 97) | 95 (93 - 97) | 96 (93 - 98) | 95 (92 - 98) | 95 (92 - 97) | 95 (92 -97) | 95 (91 - 97) | 94 (91 - 97) | 94 (91 - 97) |
| Median FiO_2_ (IQR) - fraction | 0.70 (0.55 - 0.85) | 0.65 (0.55 - 0.85) | 0.65 (0.55 - 0.80) | 0.62 (0.55 - 0.80) | 0.62 (0.55 - 0.85) | 0.70 (0.55 - 0.90) | 0.67 (0.59 - 0.80) | 0.70 (0.59 - 0.90) | 0.70 (0.60 - 0.90) | 0.70 (0.60 - 0.90) | 0.75 (0.60 - 0.90) |
| Median PaO_2_/FiO_2_ ratio (IQR) |  |  |  |  |  |  |  |  |  |  |  |
| In all systems | 15.7 (11.9 - 20.8) | 15.0 (11.6 - 19.8) | 15.2 (11.7 - 20.3) | 16.6 (12.8 - 22.2) | 17.1 (16.7 - 22.2) | 17.4 (12.4 - 21.7) | 16.5 (13.4 - 21.4) | 15.8 (11.6 - 22.0) | 15.8 (11.8 - 20.0) | 15.7 (11.4 - 20.8) | 14.9 (11.5 - 20.6) |
| In closed systems | 16.5 (12.4 - 21.6) | 16.3 (12.3 - 21.2) | 16.8 (12.9 - 21.7) | 17.5 (13.4 - 22.6) | 17.6 (13.9 - 22.8) | 18.0 (13.0 - 22.8) | 17.0 (13.9 - 21.6) | 16.0 (11.5 - 22.1) | 16.0 (11.8 - 20.0) | 15.6 (11.6 - 20.8) | 14.9 (11.6 - 20.6) |
| Median lactate concentration (IQR) - mM | 1.8 (1.1 - 3.2) | 1.4 (1.0 - 2.4) | 1.5 (1.0 - 2.4) | 1.6 (1.1 - 2.7) | 1.6 (1.1 - 2.5) | 1.7 (1.1 - 2.9) | 1.5 (1.0 - 3.0) | 2.3 (1.4 - 4.8) | 2.2 (1.4 - 3.8) | 3.6 (2.2 - 6.6) | 3.3 (2.0 - 5.9) |
| Median lowest mean arterial pressure (IQR) - mmHg | 58 (48 - 68) | 65 (55 - 75) | 66 (52 - 78) | 56 (48 - 53) | 57 (48 - 62) | 55 (45 - 62) | 55 (48 - 60) | 54 (45 - 60) | 52 (39 - 58) | 50 (37 - 56) | 49 (33 - 57) |
| Any use of inotropes, n (%) | 69 (2.4) | 6 (0.9) | 7 (1.0) | 12 (6.3) | 6 (3.5) | 3 (1.6) | 4 (2.1) | 6 (3.3) | 5 (3.0) | 5 (2.4) | 15 (6.7) |
| Any use of vasopressors, n (%) | 1578 (56.6) | 29 (4.3) | 34 (4.9) | 192 (100.0) | 174 (100.0) | 185 (100.0) | 187 (100.0) | 181 (100.0) | 167 (100.0) | 206 (100.0) | 223 (100.0) |
| Median highest dose of norepinephrine (IQR) - µg/kg/min | 0.21 (0.10 - 0.40) | 0.00 (0.00 - 0.00) | 0.00 (0.00 - 0.00) | 0.07 (0.05 - 0.10) | 0.07 (0.05 - 0.09) | 0.16 (0.14 - 0.20) | 0.16 (0.14 - 0.20) | 0.30 (0.25 - 0.32) | 0.30 (0.25 - 0.34) | 0.60 (0.48 - 0.80) | 0.60 (0.46 - 0.70) |
| Median SOFA score (IQR) | 8 (5 - 10) | 5 (4 - 7) | 5 (4 - 7) | 8 (7 -10) | 8 (7 - 10) | 9 (8 -11) | 9 (8 -11) | 10 (8 - 12) | 10 (8 - 11) | 10 (8 - 12) | 10 (8 - 12) |

Baseline characteristics of the norepinephrine dose -based subgroups. SOFA score denotes Sequential Organ Failure Assessment score, IQR interquartile range, ARDS acute respiratory distress syndrome, CPAP continuous positive airway pressure, PaO_2_ arterial partial pressure of oxygen, SaO_2_ arterial oxygen saturation, FiO_2_ fraction of inspired oxygen, n the number of patients in each group.

^a^ No patients had missing data on norepinephrine dose at baseline. Due to the zero-inflated distribution of the parameter, the number of patients in the first subgroup (norepinephrine dose: 0.00 – 0.00 µg/kg/min) is larger than the other subgroups.

^b^ Data on all patients are included. Totals from norepinephrine dose-based subgroups may not correspond to values in this column.

## Table S4a Baseline characteristics in the PaO_2_/FiO_2_ ratio-based subgroups

| PaO_2_/FiO_2_ ratio range - kPa^a^ | All patients^b^ n = 2888 | PaO_2_/FiO_2_ ratio: 4.5-11.0 n = 565 | PaO_2_/FiO_2_ ratio: 11.0-14.1 n = 584 | PaO_2_/FiO_2_ ratio: 14.1-17.4  n = 574 | PaO_2_/FiO_2_ ratio:  17.4-22.2 n = 577 | PaO_2_/FiO_2_ ratio: 22.2-157.6 n = 576 |
| --- | --- | --- | --- | --- | --- | --- |
| Median age (IQR) - years | 70 (61 - 77) | 70 (60 - 76) | 70 (62 - 77) | 70 (60 - 77) | 70 (60 - 77) | 70 (60 - 77) |
| Male sex, n (%) | 1855 (64.2) | 343 (60.7) | 373 (63.9) | 386 (67.3) | 371 (64.3) | 372 (64.6) |
| Median interval from hospital admission to randomisation (IQR) - days | 1 (0 - 5) | 2 (0 - 5) | 1 (0 - 5) | 2 (0 - 5) | 1 (0 - 5) | 1 (0 - 4) |
| Median interval from ICU admission to randomisation (IQR) - hours | 4 (2 - 7) | 3 (1 - 6) | 4 (2 - 7) | 3 (2 - 8) | 4 (2 - 8) | 3 (1 - 6) |
| Chronic co-morbidities, n (%) |  |  |  |  |  |  |
| Ischaemic heart disease | 409 (16.2) | 79 (14.0) | 80 (13.7) | 83 (14.5) | 87 (15.1) | 79 (13.7) |
| Chronic heart failure | 285 (9.9) | 62 (11.0) | 63 (10.8) | 59 (10.3) | 50 (10.4) | 41 (7.1) |
| Active metastatic cancer | 126 (4.4) | 31 (5.5) | 32 (5.5) | 21 (3.7) | 18 (3.1) | 23 (4.0) |
| Chronic dialysis | 47 (1.6) | 6 (1.1) | 10 (1.7) | 6 (1.1) | 12 (2.1) | 13 (2.3) |
| Chronic obstructive  pulmonary disease | 562 (19.5) | 72 (12.7) | 131 (22.4) | 113 (19.7) | 122 (21.1) | 124 (21.5) |
| Active haematological   cancer | 167 (5.8) | 36 (6.4) | 32 (5.5) | 33 (5.8) | 36 (6.2) | 30 (5.2) |
| Type of admission, n (%) |  |  |  |  |  |  |
| Medical | 2471 (85.6) | 513 (90.8) | 521 (89.2) | 503 (87.6) | 465 (80.6) | 459 (79.7) |
| Elective surgical | 39 (1.4) | 9 (1.6) | 4 (0.7) | 5 (0.9) | 11 (1.9) | 10 (1.7) |
| Emergency surgical | 378 (13.1) | 43 (7.6) | 59 (10.1) | 66 (11.5) | 101 (17.5) | 107 (18.6) |
| Acute illness, n (%) |  |  |  |  |  |  |
| Pneumonia | 1664 (57.6) | 380 (67.3) | 361 (61.8) | 350 (61.0) | 310 (53.7) | 261 (45.3) |
| Multiple trauma | 52 (1.8) | 8 (1.4) | 6 (1.0) | 10 (1.7) | 8 (1.4) | 17 (3.0) |
| Haemorrhagic or ischaemic   stroke | 47 (1.6) | 6 (1.1) | 7 (1.2) | 11 (1.9) | 12 (2.1) | 11 (1.9) |
| Traumatic brain injury | 22 (0.8) | 3 (0.5) | 2 (0.3) | 3 (0.5) | 4 (0.7) | 9 (1.6) |
| Myocardial infarction | 182 (6.3) | 36 (6.4) | 33 (5.7) | 32 (5.6) | 37 (6.4) | 44 (7.6) |
| Intestinal ischaemia | 68 (2.4) | 9 (1.6) | 13 (2.2) | 9 (1.6) | 14 (2.4) | 23 (4.0) |
| Cardiac arrest | 332 (11.5) | 56 (9.9) | 62 (10.6) | 56 (9.8) | 76 (13.2) | 80 (13.9) |
| ARDS | 366 (12.7) | 120 (21.2) | 79 (13.5) | 72 (12.5) | 54 (9.36) | 40 (6.9) |
| Closed system, n (%) | 2062 (71.4) | 348 (61.6) | 381 (65.2) | 405 (70.6) | 445 (77.1) | 474 (82.3) |
| Invasive mechanical   ventilation, n (%) | 1689 (58.5) | 291 (51.5) | 301 (51.5) | 311 (54.2) | 366 (63.4) | 413 (71.7) |
| Median tidal volume   (IQR) – ml | 499 (429 - 574) | 495 (406 - 578) | 478 (418 - 557) | 503 (443 - 578) | 502 (428 - 580) | 501 (438 - 570) |
| Median end expiratory   pressure (IQR) - cm H_2_O | 9 (7 - 10) | 10 (8 - 14) | 10 (8 - 12) | 9 (7 - 10) | 8 (7 - 10) | 8 (5 - 10) |
| Median peak inspiratory   pressure (IQR) - cm H_2_O | 25 (21 - 29) | 27 (24 - 31) | 26 (22 - 30) | 25 (20 - 29) | 24 (20 - 28) | 23 (20 - 28) |
| Non-invasive ventilation or   CPAP, n (%) | 373 (12.9) | 57 (10.1) | 80 (13.7) | 94 (16.4) | 79 (13.7) | 61 (10.6) |
| Median end expiratory   pressure (IQR) - cm H_2_O | 7 (5 - 9) | 8 (6 - 10) | 7 (5 - 9) | 7 (6 - 8) | 7 (5 - 8) | 7 (5 - 8) |
| Open system, n (%) | 826 (28.6) | 217 (38.4) | 203 (34.8) | 169 (29.4) | 132 (22.9) | 102 (17.7) |
| Median PaO_2_ (IQR) - kPa | 10.3 (8.7 - 12.5) | 8.4 (7.6 - 9.3) | 9.6 (8.4 - 10.9) | 9.9 (8.8 - 11.6) | 11.0 (10.1 - 12.7) | 15.6 (12.6 - 21.1) |
| Median SaO_2_ (IQR) - % | 94 (91 - 97) | 90 (87 - 93) | 93 (90 - 95) | 94 (92 - 96) | 96 (94 - 97) | 98 (97 - 99) |
| Median FiO_2_ (IQR) - fraction | 0.70 (0.55 - 0.85) | 1.00 (0.90 - 1.00) | 0.80 (0.70 - 0.85) | 0.60 (0.56 - 0.70) | 0.60 (0.50 - 0.65) | 0.55 (0.50 - 0.62) |
| Median PaO_2_/FiO_2_ ratio (IQR) |  |  |  |  |  |  |
| In all systems | 15.7 (11.9 - 20.8) | 9.1 (8.1 - 10.1) | 12.7 (11.9 - 13.4) | 15.7 (15.0 - 16.6) | 19.4 (18.4 - 20.7) | 28.0 (24.1 - 35.5) |
| In closed systems | 16.5 (12.4 - 21.6) | 9.1 (8.1 - 10.0) | 12.7 (11.9 - 13.4) | 15.7 (15.0 - 16.6) | 19.4 (18.5 - 20.7) | 28.5 (24.2 - 35.5) |
| Median lactate concentration (IQR) - mM | 1.8 (1.1 - 3.2) | 1.9 (1.2 - 3.4) | 1.8 (1.2 - 3.3) | 1.7 (1.1 - 2.9) | 1.6 (1.1 - 2.9) | 1.8 (1.1 - 3.5) |
| Median lowest mean arterial pressure (IQR) - mmHg | 58 (48 - 68) | 59 (48 - 70) | 58 (48 - 67) | 60 (50 - 69) | 58 (49 - 67) | 56 (47 - 67) |
| Any use of inotropes, n (%) | 69 (2.4) | 13 (2.3) | 13 (2.2) | 13 (2.2) | 15 (2.6) | 15 (2.6) |
| Any use of vasopressors, n (%) | 1578 (56.6) | 289 (51.2) | 305 (52.2) | 296 (51.6) | 336 (58.2) | 346 (60.1) |
| Median highest dose of norepinephrine (IQR) - µg/kg/min | 0.21 (0.10 - 0.40) | 0.25 (0.11 - 0.49) | 0.24 (0.10 - 0.42) | 0.20 (0.10 - 0.39) | 0.20 (0.10 - 0.39) | 0.20 (0.10 - 0.39) |
| Median SOFA score (IQR) | 8 (5 - 10) | 8 (6 - 10) | 8 (5 - 10) | 7 (5 - 10) | 8 (6 - 10) | 7 (5 - 9) |

Baseline characteristics of the PaO_2_/FiO_2_ ratio-based subgroups. SOFA score denotes Sequential Organ Failure Assessment score, IQR interquartile range, ARDS acute respiratory distress syndrome, CPAP continuous positive airway pressure, PaO_2_ arterial partial pressure of oxygen, SaO_2_ arterial oxygen saturation, FiO_2_ fraction of inspired oxygen, n the number of patients in each group.

^a^ Lower PaO_2_/FiO_2_ ratios indicate more severe pulmonary dysfunction. In total, 12 of 2888 patients had a missing PaO_2_/FiO_2_ ratio at baseline.

^b^ Data on all patients are included. Totals from norepinephrine PaO_2_/FiO_2_ ratio-based subgroups may not correspond to values in this column.

## Table S4b Baseline characteristics in the PaO_2_/FiO_2_ ratio-based subgroups stratified by treatment

| PaO_2_/FiO_2_ ratio range - kPa^a^ | All patients n = 2888 | PaO_2_/FiO_2_ ratio: 4.5-11.0 | | PaO_2_/FiO_2_ ratio: 11.0-14.1 | | PaO_2_/FiO_2_ ratio: 14.1-17.4 | | PaO_2_/FiO_2_ ratio: 17.4-22.2 | | PaO_2_/FiO_2_ ratio: 22.2-157.6 | |
| --- | --- | --- | --- | --- | --- | --- | --- | --- | --- | --- | --- |
|  |  | **Lower target n = 293** | **Higher target n = 272** | **Lower target n = 279** | **Higher target n = 305** | **Lower target n = 290** | **Higher target n = 284** | **Lower target n = 284** | **Higher target n = 293** | **Lower target n = 290** | **Higher target n = 286** |
| Median age (IQR) - years | 70 (61 - 77) | 70 (60 - 76) | 70 (59 - 77) | 71 (63 - 78) | 69 (61 - 76) | 68 (59 - 76) | 71 (61 - 77) | 70 (60 - 76) | 70 (59 - 78) | 71 (61 - 78) | 69 (60 - 76) |
| Male sex, n (%) | 1855 (64.2) | 186 (63.5) | 157 (57.7) | 170 (60.9) | 203 (66.6) | 194 (66.9) | 192 (67.6) | 181 (63.7) | 190 (64.9) | 180 (62.1) | 192 (67.1) |
| Median interval from hospital admission to randomisation (IQR) - days | 1 (0 - 5) | 2 (1 - 4) | 2 (0 - 5) | 1 (0 - 5) | 1 (0 - 5) | 2 (1 - 5) | 2 (0 - 5) | 1 (0 - 5) | 1 (0 - 5) | 1 (0 - 5) | 1 (0 - 3) |
| Median interval from ICU admission to randomisation (IQR) - hours | 4 (2 - 7) | 3 (1 - 6) | 3 (1 - 7) | 4 (2 - 7) | 4 (2 - 8) | 4 (2 - 8) | 3 (2 - 8) | 4 (2 - 9= | 5 (2 - 8) | 4 (2 - 7) | 3 (1 - 6) |
| Chronic co-morbidities, n (%) |  |  |  |  |  |  |  |  |  |  |  |
| Ischaemic heart disease | 409 (16.2) | 41 (14.0) | 38 (14.0) | 34 (12.2) | 46 (15.1) | 35 (12.1) | 48 (16.9) | 46 (16.2) | 41 (14.0) | 48 (16.6) | 31 (10.8) |
| Chronic heart failure | 285 (9.9) | 30 (10.2) | 32 (11.8) | 28 (10.0) | 35 (11.5) | 24 (8.3) | 35 (12.3) | 36 (12.7) | 24 (8.2) | 21 (7.2) | 20 (7.0) |
| Active metastatic cancer | 126 (4.4) | 20 (6.8) | 11 (4.0) | 16 (5.7) | 16 (5.3) | 8 (2.8) | 13 (4.6) | 8 (2.8) | 10 (3.4) | 13 (4.5) | 10 (3.5) |
| Chronic dialysis | 47 (1.6) | 2 (0.7) | 4 (1.5) | 2 (0.7) | 8 (2.6) | 1 (0.3) | 5 (1.8) | 6 (2.1) | 6 (2.1) | 8 (2.8) | 5 (1.8) |
| Chronic obstructive  pulmonary disease | 562 (19.5) | 31 (10.6) | 41 (15.1) | 68 (24.4) | 63 (20.7) | 51 (17.6) | 62 (21.8) | 62 (21.8) | 60 (20.5) | 65 (22.4) | 59 (20.6) |
| Active haematological   cancer | 167 (5.8) | 18 (6.1) | 18 (6.6) | 13 (4.7) | 19 (6.2) | 18 (6.2) | 15 (5.3) | 19 (6.7) | 17 (5.8) | 13 (4.5) | 17 (5.9) |
| Type of admission, n (%) |  |  |  |  |  |  |  |  |  |  |  |
| Medical | 2471 (85.6) | 269 (91.8) | 244 (89.7) | 247 (88.5) | 274 (89.8) | 257 (88.6) | 246 (86.6) | 234 (82.4) | 231 (78.8) | 227 (78.3) | 232 (81.1) |
| Elective surgical | 39 (1.4) | 3 (1.0) | 6 (2.2) | 3 (1.1) | 1 (0.3) | 3 (1.0) | 2 (0.7) | 4 (1.4) | 7 (2.4) | 5 (1.7) | 5 (1.8) |
| Emergency surgical | 378 (13.1) | 21 (7.2) | 22 (8.1) | 29 (10.4) | 30 (9.8) | 30 (10.3) | 36 (12.7) | 46 (16.2) | 55 (18.8) | 58 (20.0) | 49 (17.1) |
| Acute illness, n (%) |  |  |  |  |  |  |  |  |  |  |  |
| Pneumonia | 1664 (57.6) | 197 (67.2) | 183 (67.3) | 166 (59.5) | 195 (63.9) | 176 (61.0) | 174 (61.3) | 157 (55.3) | 153 (52.2) | 136 (46.9) | 125 (43.7) |
| Multiple trauma | 52 (1.8) | 3 (1.0) | 5 (1.8) | 1 (0.4) | 5 (1.6) | 6 (2.1) | 4 (1.4) | 4 (1.4) | 4 (1.4) | 8 (2.8) | 9 (3.2) |
| Haemorrhagic or ischaemic   stroke | 47 (1.6) | 2 (0.7) | 4 (1.5) | 4 (1.4) | 3 (1.0) | 7 (2.4) | 4 (1.4) | 6 (2.1) | 6 (2.1) | 6 (2.1) | 5 (1.8) |
| Traumatic brain injury | 22 (0.8) | 2 (0.7) | 1 (0.4) | 1 (0.4) | 1 (0.3) | 0 (0.0) | 3 (1.1) | 1 (0.4) | 3 (1.0) | 4 (1.4) | 5 (1.8) |
| Myocardial infarction | 182 (6.3) | 16 (5.5) | 20 (7.4) | 13 (4.7) | 20 (6.6) | 15 (5.2) | 17 (6.0) | 20 (7.0) | 17 (5.8) | 20 (6.9) | 24 (8.4) |
| Intestinal ischaemia | 68 (2.4) | 4 (1.4) | 5 (1.8) | 7 (2.5) | 6 (2.0) | 1 (0.3) | 8 (2.8) | 6 (2.1) | 8 (2.7) | 9 (3.1) | 14 (4.9) |
| Cardiac arrest | 332 (11.5) | 23 (7.9) | 33 (12.1) | 27 (9.7) | 35 (11.5) | 25 (8.6) | 31 (10.9) | 34 (12.0) | 42 (14.3) | 37 (12.8) | 43 (15.0) |
| ARDS | 366 (12.7) | 62 (21.2) | 58 (21.3) | 37 (13.3) | 42 (13.8) | 29 (10.0) | 43 (15.1) | 25 (8.8) | 29 (9.9) | 22 (7.6) | 18 (6.3) |
| Closed system, n (%) | 2062 (71.4) | 187 (63.8) | 161 (59.2) | 180 (65.5) | 201 (65.9) | 201 (69.3) | 204 (71.8) | 217 (76.4) | 228 (77.8) | 236 (81.4) | 234 (83.2) |
| Invasive mechanical   ventilation, n (%) | 1689 (58.5) | 152 (51.9) | 139 (51.1) | 141 (50.5) | 160 (52.5) | 151 (52.1) | 160 (56.3) | 180 (63.4) | 186 (63.5) | 200 (69.0) | 213 (74.5) |
| Median tidal volume   (IQR) – ml | 499 (429 - 574) | 496 (406 - 578) | 495 (407 - 579) | 469 (407 - 538) | 481 (430 - 579) | 499 (443 - 551) | 508 (443 - 607) | 508 (432 - 580) | 499 (422 - 579) | 500 (440 - 565) | 502 (435 - 574) |
| Median end expiratory   pressure (IQR) - cm H_2_O | 9 (7 - 10) | 10 (8 - 14) | 10 (8 - 14) | 10 (8 - 11) | 10 (8 - 12) | 10 (7 - 12) | 8 (7 - 10) | 8 (7 -10) | 8 (6 - 10) | 8 (6 - 10) | 8 (5 - 10) |
| Median peak inspiratory   pressure (IQR) - cm H_2_O | 25 (21 - 29) | 28 (24 - 31) | 27 (23 - 31) | 25 (22 - 30) | 26 (22 - 30) | 25 (22 - 29) | 24 (20 - 28) | 24 (20 - 28) | 24 (20 - 28) | 23 (20 - 28) | 24 (21 - 29) |
| Non-invasive ventilation or   CPAP, n (%) | 373 (12.9) | 35 (12.0) | 22 (8.1) | 39 (14.0) | 41 (13.4) | 50 (17.2) | 44 (15.5) | 37 (13.0) | 42 (14.3) | 36 (12.4) | 25 (8.7) |
| Median end expiratory   pressure (IQR) - cm H_2_O | 7 (5 - 9) | 7 (5 - 10) | 8 (7 - 10) | 7 (6 - 8) | 7 (5 - 9) | 7 (6 - 8) | 8 (6 - 8) | 6 (5 - 8) | 8 (6 - 8) | 6 (5 - 8) | 8 (5 - 10) |
| Open system, n (%) | 826 (28.6) | 106 (36.2) | 111 (40.8) | 99 (35.5) | 104 (34.1) | 89 (30.7) | 80 (28.2) | 67 (23.6) | 65 (22.2) | 54 (18.6) | 48 (16.8) |
| Median PaO_2_ (IQR) - kPa | 10.3 (8.7 - 12.5) | 8.5 (7.6 - 9.4) | 8.4 (7.6 - 9.3) | 9.5 (8.3 - 10.9) | 9.7 (8.5 - 11.0) | 9.8 (8.9 - 11.7) | 10.0 (8.8 - 11.5) | 11.0 (10.2 - 12.9) | 11.0 (10.1 - 12.6) | 16.0 (12.5 - 21.5) | 15.3 (12.7 - 20.8) |
| Median SaO_2_ (IQR) - % | 94 (91 - 97) | 91 (87 - 93) | 90 (86 - 93) | 93 (90 - 95) | 93 (91 - 95) | 94 (92 - 96) | 94 (92 - 96) | 96 (94 - 97) | 96 (94 - 97) | 98 (96 - 99) | 98 (97 - 99) |
| Median FiO_2_ (IQR) - fraction | 0.70 (0.55 - 0.85) | 1.00 (0.90 - 1.00) | 1.00 (0.90 - 1.00) | 0.75 (0.65 - 0.85) | 0.80 (0.70 - 0.90) | 0.60 (0.55 - 0.75) | 0.60 (0.59 - 0.70) | 0.60 (0.50 - 0.65) | 0.60 (0.50 - 0.65) | 0.55 (0.50 - 0.65) | 0.54 (0.50 - 0.60) |
| Median PaO_2_/FiO_2_ ratio (IQR) |  |  |  |  |  |  |  |  |  |  |  |
| In all systems | 15.7 (11.9 - 20.8) | 9.2 (8.1 - 10.1) | 9.1 (8.1 - 10.0) | 12.7 (11.9 - 13.5) | 12.6 (11.8 - 13.4) | 15.8 (15.0 - 16.6) | 15.7 (14.9 - 16.5) | 19.6 (18.5 - 21.0) | 19.3 (18.4 - 20.5) | 28.6 (24.4 - 35.8) | 27.4 (23.8 - 34.6) |
| In closed systems | 16.5 (12.4 - 21.6) | 9.0 (7.9 - 10.0) | 9.3 (8.1 - 10-1) | 12.7 (11.9 - 13.5) | 12.6 (11.9 - 13.4) | 15.8 (15.0 - 16.7) | 15.6 (15.0 - 16.5) | 19.6 (18.5 - 20.9) | 19.3 (18.4 - 20.5) | 28.7 (24.3 - 35.7) | 28.1 (24.2 - 35.0) |
| Median lactate concentration (IQR) - mM | 1.8 (1.1 - 3.2) | 2.0 (1.2 - 3.5) | 1.9 (1.2 - 3.3) | 1.8 (1.1 - 3.3) | 1.7 (1.2 - 3.2) | 1.7 (1.1 - 2.8) | 1.7 (1.2 - 3.0) | 1.6 (1.0 - 3.0) | 1.6 (1.1 - 2.9) | 1.9 (1.1 - 3.6) | 1.8 (1.1 - 3.3) |
| Median lowest mean arterial pressure (IQR) - mmHg | 58 (48 - 68) | 58 (47 - 67) | 60 (49 - 73) | 58 (49 - 68) | 58 (48 - 66) | 60 (51 - 69) | 60 (50 - 69) | 59 (50 - 68) | 56 (48 - 67) | 57 (47 - 67) | 56 (45 - 67) |
| Any use of inotropes, n (%) | 69 (2.4) | 5 (1.7) | 8 (2.9) | 4 (1.4) | 9 (3.0) | 7 (2.4) | 6 (2.1) | 10 (3.5) | 5 (1.7) | 6 (2.1) | 9 (3.2) |
| Any use of vasopressors, n (%) | 1578 (56.6) |  |  |  |  |  |  |  |  |  |  |
| Median highest dose of norepinephrine (IQR) - µg/kg/min | 0.21 (0.10 - 0.40) | 0.25 (0.12 - 0.44) | 0.25 (0.10 - 0.50) | 0.22 (0.09 - 0.40) | 0.25 (0.13 - 0.45) | 0.21 (0.10 - 0.40) | 0.20 (0.12 - 0.38) | 0.20 (0.10 - 0.39) | 0.20 (0.10 - 0.39) | 0.20 (0.10 - 0.37) | 0.20 (0.10 - 0.40) |
| Median SOFA score (IQR) | 8 (5 - 10) | 8 (6 - 10) | 8 (6 - 10) | 8 (5 - 10) | 8 (6 - 10) | 7 (5 - 9) | 7 (5 - 10) | 8 (6 - 10) | 8 (5 - 10) | 7 (5 - 9) | 7 (5 - 10) |

Baseline characteristics of the PaO_2_/FiO_2_ ratio-based subgroups. SOFA score denotes Sequential Organ Failure Assessment score, IQR interquartile range, ARDS acute respiratory distress syndrome, CPAP continuous positive airway pressure, PaO_2_ arterial partial pressure of oxygen, SaO_2_ arterial oxygen saturation, FiO_2_ fraction of inspired oxygen, n the number of patients in each group.

^a^ Lower PaO_2_/FiO_2_ ratios indicate more severe pulmonary dysfunction. In total, 12 of 2888 patients had a missing PaO_2_/FiO_2_ ratio at baseline.

^b^ Data on all patients are included. Totals from norepinephrine PaO_2_/FiO_2_ ratio-based subgroups may not correspond to values in this column.

## Table S5 Odds ratios for 90-day all-cause mortality in the primary analysis using weakly informative priors

| Group | Odds ratio | |
| --- | --- | --- |
| All patients | 1.03 (95% CrI: 0.88 to 1.19) | |
| Baseline SOFA score^a^ | | |
| SOFA score: 0-4 | 1.04 (95% CrI: 0.78 to 1.35) | |
| SOFA score: 5-6 | 1.00 (95% CrI: 0.73 to 1.26) | |
| SOFA score: 7-7 | 1.16 (95% CrI: 0.90 to 1.82) | |
| SOFA score: 8-10 | 1.03 (95% CrI: 0.82 to 1.28) | |
| SOFA score: 11-19 | 1.06 (95% CrI: 0.83 to 1.37) | |
| Baseline lactate concentration (mM) | | |
| Lactate: 0.2-0.9 | 0.90 (95% CrI: 0.58 to 1.18) | |
| Lactate: 1.0-1.4 | 1.01 (95% CrI: 0.76 to 1.28) | |
| Lactate: 1.5-2.1 | 1.14 (95% CrI: 0.89 to 1.59) | |
| Lactate: 2.2-3.6 | 1.10 (95% CrI: 0.87 to 1.48) | |
| Lactate: 3.7-24.0 | 1.04 (95% CrI: 0.79 to 1.35) | |
| Baseline norepinephrine dose (µg/kg/min) | | |
| Norepinephrine: 0.00-0.00 | 0.98 (95% CrI: 0.80 to 1.19) | |
| Norepinephrine: 0.01-0.10 | 1.00 (95% CrI: 0.72 to 1.30) | |
| Norepinephrine: 0.11-0.21 | 1.01 (95% CrI: 0.74 to 1.33) | |
| Norepinephrine: 0.22-0.39 | 1.07 (95% CrI: 0.82 to 1.52) | |
| Norepinephrine: 0.40-2.40 | 1.17 (95% CrI: 0.90 to 1.76) | |
| Baseline PaO_2_/FiO_2_ ratio (kPa) | |  |
| PaO_2_/FiO_2_ ratio: 4.5-11.0 | 1.03 (95% CrI: 0.82 to 1.27) | |
| PaO_2_/FiO_2_ ratio: 11.0-14.1 | 1.06 (95% CrI: 0.86 to 1.35) | |
| PaO_2_/FiO_2_ ratio: 14.1-17.4 | 1.05 (95% CrI: 0.86 to 1.33) | |
| PaO_2_/FiO_2_ ratio: 17.4-22.2 | 0.98 (95% CrI: 0.74 to 1.20) | |
| PaO_2_/FiO_2_ ratio: 22.2-157.6 | 1.04 (95% CrI: 0.84 to 1.30) | |

SOFA score denotes Sequential Organ Failure Assessment score, PaO_2_ arterial partial pressure of oxygen, FiO_2_ fraction of inspired oxygen, CrI denotes credibility interval.

^a^ The aggregated SOFA score ranges from 0-24, with sub-score from 0-4 for 6 organ systems (respiration, coagulation, liver, cardiovascular, central nervous system, and renal), with higher scores indication higher degrees of organ failure.

^b^ Lower PaO_2_/FiO_2_ ratios indicate more severe pulmonary dysfunction.

## Table S6 Posterior probabilities of different effect sizes for 90-day all-cause mortality

| Group | RR < 0.80 | RR < 0.85 | RR < 0.90 | RR < 0.95 | RR < 0.99 | RR < 1.00 | RR > 1.00 | RR > 1.01 | RR > 1.05 | RR > 1.11 | RR > 1.18 | RR > 1.25 | |
| --- | --- | --- | --- | --- | --- | --- | --- | --- | --- | --- | --- | --- | --- |
| All patients | 0.0% | 0.0% | 0.3% | 6.4% | 28.1% | 36.5% | 63.5% | 54.5% | 20.0% | 2.0% | 0.1% | 0.0% | |
| Baseline SOFA score^a^ | | | | | | | | | | | | | |
| SOFA score: 0-4 | 0.9% | 2.6% | 7.4% | 18.2% | 33.2% | 37.8% | 62.2% | 57.3% | 37.6% | 17.3% | 5.9% | 1.6% | |
| SOFA score: 5-6 | 1.8% | 5.2% | 13.3% | 28.1% | 45.3% | 50.1% | 49.9% | 45.0% | 25.4% | 8.5% | 1.8% | 0.3% | |
| SOFA score: 7-7 | 0.0% | 0.1% | 0.7% | 3.8% | 11.0% | 13.8% | 86.2% | 83.0% | 67.6% | 46.9% | 30.1% | 17.8% | |
| SOFA score: 8-10 | 0.1% | 0.5% | 3.3% | 13.9% | 32.8% | 38.7% | 61.3% | 54.8% | 29.3% | 8.0% | 1.2% | 0.1% | |
| SOFA score: 11-19 | 0.0% | 0.1% | 1.1% | 7.3% | 24.3% | 31.2% | 68.8% | 61.3% | 29.8% | 7.1% | 1.0% | 0.1% | |
| Baseline lactate concentration (mM) | | | | | | | | | | | | | |
| Lactate: 0.2-0.9 | 19.2% | 30.1% | 42.9% | 57.4% | 70.1% | 72.9% | 27.1% | 24.2% | 13.3% | 4.6% | 1.0% | 0.2% | |
| Lactate: 1.0-1.4 | 0.6% | 2.8% | 9.6% | 24.6% | 42.6% | 47.8% | 52.2% | 47.0% | 26.2% | 8.6% | 1.9% | 0.3% | |
| Lactate: 1.5-2.1 | 0.0% | 0.1% | 0.7% | 4.1% | 13.0% | 16.1% | 83.9% | 80.3% | 62.0% | 37.2% | 18.4% | 7.2% | |
| Lactate: 2.2-3.6 | 0.0% | 0.1% | 1.1% | 6.1% | 17.7% | 22.1% | 77.9% | 73.1% | 51.3% | 24.9% | 9.4% | 2.5% | |
| Lactate: 3.7-24.0 | 0.0% | 0.1% | 1.4% | 9.7% | 30.2% | 38.1% | 61.9% | 53.6% | 22.1% | 4.0% | 0.4% | 0.0% | |
| Baseline norepinephrine dose (µg/kg/min) | | | | | | | | | | | | |  |
| Norepinephrine: 0.00-0.00 | 0.1% | 1.2% | 7.4% | 26.3% | 50.2% | 56.8% | 43.2% | 37.0% | 15.2% | 2.6% | 0.2% | 0.0% | |
| Norepinephrine: 0.01-0.10 | 1.6% | 4.9% | 12.9% | 28.1% | 46.4% | 51.5% | 48.5% | 43.9% | 24.0% | 8.1% | 2.1% | 0.4% | |
| Norepinephrine: 0.11-0.21 | 1.2% | 3.9% | 10.6% | 24.7% | 41.9% | 47.0% | 53.0% | 47.9% | 27.6% | 10.4% | 3.1% | 0.7% | |
| Norepinephrine: 0.22-0.39 | 0.1% | 0.4% | 2.1% | 9.5% | 24.5% | 29.6% | 70.4% | 65.2% | 41.4% | 19.1% | 7.3% | 2.1% | |
| Norepinephrine: 0.40-2.40 | 0.0% | 0.0% | 0.2% | 2.6% | 10.8% | 14.0% | 86.0% | 82.1% | 62.6% | 38.3% | 20.2% | 8.1% | |
| Baseline PaO_2_/FiO_2_ ratio (kPa)^b^ | | | | | | | | | | | | | |
| PaO_2_/FiO_2_ ratio: 4.5-11.0 | 0.1% | 0.7% | 3.7% | 14.7% | 33.4% | 39.3% | 60.7% | 54.3% | 28.9% | 8.0% | 1.2% | 0.1% | |
| PaO_2_/FiO_2_ ratio: 11.0-14.1 | 0.0% | 0.2% | 1.3% | 7.7% | 22.6% | 28.0% | 72.0% | 66.3% | 40.4% | 15.5% | 4.1% | 0.7% | |
| PaO_2_/FiO_2_ ratio: 14.1-17.4 | 0.0% | 0.3% | 1.6% | 8.7% | 24.8% | 30.3% | 69.7% | 64.0% | 38.6% | 14.0% | 3.4% | 0.6% | |
| PaO_2_/FiO_2_ ratio: 17.4-22.2 | 1.5% | 5.2% | 13.9% | 31.6% | 51.8% | 57.4% | 42.6% | 36.9% | 17.0% | 3.7% | 0.5% | 0.0% | |
| PaO_2_/FiO_2_ ratio: 22.2-157.6 | 0.1% | 0.5% | 2.6% | 11.8% | 29.1% | 34.6% | 65.4% | 59.6% | 34.4% | 11.9% | 2.9% | 0.5% | |

SOFA score denotes Sequential Organ Failure Assessment score, PaO_2_ arterial partial pressure of oxygen, FiO_2_ fraction of inspired oxygen. RRs < 1 favours the lower oxygenation target; RRs > 1 favours the higher oxygenation target.

^a^ SOFA score ranges from 0-24, with sub-score from 0-4 for 6 organ system (respiration, coagulation, liver, cardiovascular, central nervous system, and renal), with higher aggregated scores indication higher degrees of organ failure.

^b^ PaO_2_/FiO_2_ ratio: lower scores indicate more severe pulmonary dysfunction.

## Table S7 Summarised effect measures for 90-day all-cause mortality in the sensitivity analysis using evidence-based priors

| Group | n | Event probability, lower target | Event probability, higher target | Relative risk | Risk difference | Odds ratio |
| --- | --- | --- | --- | --- | --- | --- |
| All patients | 2888 | 42.7% (95% CrI: 38.2% to 47.4%) | 42.7% (95% CrI: 38.0% to 47.3%) | 1.00 (95% CrI: 0.93 to 1.08) | 0.0% (95% CrI: -3.1% to 3.2%) | 1.00 (95% CrI: 0.88 to 1.14) |
| Baseline SOFA score^a^ | 2799 |  |  |  |  |  |
| SOFA score: 0-4 | 486 | 32.2% (95% CrI: 26.2% to 38.7%) | 32.0% (95% CrI: 26.1% to 38.4%) | 1.01 (95% CrI: 0.84 to 1.19) | 0.3% (95% CrI: -5.5% to 5.7%) | 1.01 (95% CrI: 0.78 to 1.30) |
| SOFA score: 5-6 | 501 | 35.3% (95% CrI: 29.1% to 41.8%) | 36.0% (95% CrI: 29.8% to 42.8%) | 0.99 (95% CrI: 0.81 to 1.13) | -0.5% (95% CrI: -7.5% to 4.5%) | 0.98 (95% CrI: 0.72 to 1.22) |
| SOFA score: 7-7 | 352 | 37.1% (95% CrI: 30.2% to 45.0%) | 34.1% (95% CrI: 26.8% to 41.3%) | 1.07 (95% CrI: 0.93 to 1.43) | 2.4% (95% CrI: -2.7% to 12.4%) | 1.11 (95% CrI: 0.89 to 1.73) |
| SOFA score: 8-10 | 881 | 41.9% (95% CrI: 36.1% to 47.8%) | 41.7% (95% CrI: 36.1% to 47.5%) | 1.01 (95% CrI: 0.89 to 1.13) | 0.2% (95% CrI: -5.1% to 5.0%) | 1.01 (95% CrI: 0.81 to 1.23) |
| SOFA score: 11-19 | 579 | 56.8% (95% CrI: 50.5% to 63.1%) | 56.0% (95% CrI: 49.8% to 62.2%) | 1.01 (95% CrI: 0.92 to 1.13) | 0.7% (95% CrI: -4.9% to 6.7%) | 1.03 (95% CrI: 0.82 to 1.31) |
| Baseline lactate concentration [mM]^b^ | 2869 |  |  |  |  |  |
| Lactate concentration: 0.2-0.9 | 501 | 22.9% (95% CrI: 17.4% to 28.8%) | 25.5% (95% CrI: 20.0% to 32.2%) | 0.92 (95% CrI: 0.66 to 1.10) | -2.1% (95% CrI: -9.9% to 2.4%) | 0.89 (95% CrI: 0.58 to 1.14) |
| Lactate concentration: 1.0-1.4 | 631 | 37.9% (95% CrI: 32.0% to 44.1%) | 38.3% (95% CrI: 32.3% to 44.7%) | 0.99 (95% CrI: 0.84 to 1.15) | -0.3% (95% CrI: -6.4% to 5.2%) | 0.99 (95% CrI: 0.76 to 1.25) |
| Lactate concentration: 1.5-2.1 | 577 | 41.7% (95% CrI: 35.1% to 48.8%) | 39.0% (95% CrI: 32.6% to 45.4%) | 1.06 (95% CrI: 0.93 to 1.30) | 2.4% (95% CrI: -3.1% to 10.4%) | 1.10 (95% CrI: 0.88 to 1.54) |
| Lactate concentration: 2.2-3.6 | 576 | 44.7% (95% CrI: 38.5% to 51.3%) | 42.8% (95% CrI: 36.4% to 49.2%) | 1.04 (95% CrI: 0.92 to 1.23) | 1.7% (95% CrI: -3.8% to 9.0%) | 1.07 (95% CrI: 0.86 to 1.45) |
| Lactate concentration: 3.7-24.0 | 584 | 61.4% (95% CrI: 55.0% to 67.6%) | 61.0% (95% CrI: 54.6% to 67.3%) | 1.01 (95% CrI: 0.91 to 1.11) | 0.3% (95% CrI: -5.6% to 6.5%) | 1.01 (95% CrI: 0.79 to 1.32) |
| Baseline norepinephrine dose [µg/kg/min]^c^ | 2888 |  |  |  |  |  |
| Norepinephrine dose: 0.00-0.00 | 1373 | 37.9% (95% CrI: 32.8% to 43.3%) | 38.7% (95% CrI: 33.5% to 44.1%) | 0.98 (95% CrI: 0.87 to 1.09) | -0.7% (95% CrI: -5.5% to 3.4%) | 0.97 (95% CrI: 0.79 to 1.15) |
| Norepinephrine dose: 0.01-0.10 | 366 | 39.5% (95% CrI: 32.5% to 46.9%) | 40.3% (95% CrI: 33.4% to 47.7%) | 0.99 (95% CrI: 0.82 to 1.14) | -0.6% (95% CrI: -8.0% to 5.1%) | 0.98 (95% CrI: 0.72 to 1.24) |
| Norepinephrine dose: 0.11-0.21 | 372 | 39.2% (95% CrI: 32.3% to 46.6%) | 39.7% (95% CrI: 32.9% to 46.7%) | 0.99 (95% CrI: 0.83 to 1.16) | -0.3% (95% CrI: -7.2% to 5.7%) | 0.99 (95% CrI: 0.74 to 1.27) |
| Norepinephrine dose: 0.22-0.39 | 348 | 49.5% (95% CrI: 42.1% to 57.0%) | 48.3% (95% CrI: 40.8% to 55.8%) | 1.02 (95% CrI: 0.90 to 1.21) | 0.9% (95% CrI: -5.2% to 9.1%) | 1.04 (95% CrI: 0.81 to 1.45) |
| Norepinephrine dose: 0.40-2.40 | 429 | 52.0% (95% CrI: 45.0% to 59.6%) | 48.6% (95% CrI: 41.3% to 55.6%) | 1.06 (95% CrI: 0.94 to 1.30) | 2.7% (95% CrI: -2.9% to 13.1%) | 1.11 (95% CrI: 0.89 to 1.70) |
| Baseline PaO_2_/FiO_2_ ratio (kPa)^d^ | 2876 |  |  |  |  |  |
| PaO_2_/FiO_2_ ratio: 4.5-11.0 | 565 | 45.6% (95% CrI: 39.5% to 52.0%) | 45.5% (95% CrI: 39.8% to 51.6%) | 1.00 (95% CrI: 0.90 to 1.11) | 0.1% (95% CrI: -4.9% to 4.9%) | 1.00 (95% CrI: 0.82 to 1.22) |
| PaO_2_/FiO_2_ ratio: 11.0-14.1 | 584 | 46.3% (95% CrI: 40.1% to 52.8%) | 45.3% (95% CrI: 39.6% to 51.4%) | 1.02 (95% CrI: 0.92 to 1.15) | 0.8% (95% CrI: -3.7% to 6.5%) | 1.03 (95% CrI: 0.86 to 1.30) |
| PaO_2_/FiO_2_ ratio: 14.1-17.4 | 574 | 46.2% (95% CrI: 40.3% to 52.6%) | 45.4% (95% CrI: 39.7% to 51.6%) | 1.01 (95% CrI: 0.92 to 1.14) | 0.7% (95% CrI: -3.9% to 6.2%) | 1.03 (95% CrI: 0.86 to 1.29) |
| PaO_2_/FiO_2_ ratio: 17.4-22.2 | 577 | 41.4% (95% CrI: 34.7% to 47.8%) | 42.6% (95% CrI: 36.4% to 48.5%) | 0.98 (95% CrI: 0.84 to 1.09) | -1.0% (95% CrI: -7.3% to 3.5%) | 0.96 (95% CrI: 0.74 to 1.15) |
| PaO_2_/FiO_2_ ratio: 22.2-157.6 | 576 | 43.6% (95% CrI: 37.5% to 50.0%) | 43.2% (95% CrI: 37.2% to 49.0%) | 1.01 (95% CrI: 0.90 to 1.14) | 0.4% (95% CrI: -4.3% to 5.7%) | 1.02 (95% CrI: 0.84 to 1.26) |

Posterior event probabilities, relative risks, risk differences and odd ratios for mortality. CrI denotes credibility interval, SOFA score denotes Sequential Organ Failure Assessment score, PaO_2_ arterial partial pressure of oxygen, FiO_2_ fraction of inspired oxygen, n the number of patients in each group (after excluding patients with missing data for one or more variables included in the analyses).

^a^ SOFA score ranges from 0-24, with sub-score from 0-4 for 6 organ system (respiration, coagulation, liver, cardiovascular, central nervous system, and renal), with higher aggregated scores indication higher degrees of organ failure.

^b^ Lower PaO_2_/FiO_2_ ratios indicate more severe pulmonary dysfunction.

## Table S8 Posterior probabilities of different effect sizes of 90-day all-cause mortality in the s**ensitivity analysis using evidence-based priors**

| Group | RR < 0.80 | RR < 0.85 | RR < 0.90 | RR < 0.95 | RR < 0.99 | RR < 1.00 | RR > 1.00 | RR > 1.01 | RR > 1.05 | RR > 1.11 | RR > 1.18 | RR > 1.25 | | |
| --- | --- | --- | --- | --- | --- | --- | --- | --- | --- | --- | --- | --- | --- | --- |
| All patients | 0.0% | 0.0% | 0.3% | 8.4% | 38.6% | 49.1% | 50.9% | 40.6% | 9.4% | 0.4% | 0.0% | 0.0% | | |
| Baseline SOFA score^a^ | | | | | | | | | | | | | |  |
| SOFA score: 0-4 | 0.9% | 0.8% | 2.9% | 8.8% | 22.0% | 40.3% | 45.6% | 54.4% | 49.0% | 27.5% | 10.3% | 3.3% | | |
| SOFA score: 5-6 | 1.8% | 1.9% | 5.8% | 14.9% | 32.1% | 52.5% | 58.2% | 41.8% | 36.3% | 16.8% | 4.3% | 0.9% | | |
| SOFA score: 7-7 | 0.0% | 0.0% | 0.1% | 1.0% | 5.4% | 16.1% | 19.9% | 80.1% | 75.9% | 56.9% | 36.3% | 21.8% | | |
| SOFA score: 8-10 | 0.1% | 0.1% | 0.6% | 4.0% | 16.7% | 39.3% | 46.3% | 53.7% | 46.5% | 20.0% | 4.0% | 0.5% | | |
| SOFA score: 11-19 | 0.0% | 0.0% | 0.2% | 1.3% | 9.1% | 30.9% | 38.8% | 61.2% | 52.6% | 20.6% | 3.9% | 0.6% | | |
| Baseline lactate concentration (mM) | | | | | | | | | | | | | |  |
| Lactate: 0.2-0.9 | 19.4% | 30.6% | 44.9% | 60.8% | 74.9% | 78.4% | 21.6% | 18.6% | 8.5% | 2.0% | 0.3% | 0.1% | | |
| Lactate: 1.0-1.4 | 0.6% | 3.0% | 10.6% | 27.8% | 49.2% | 54.8% | 45.2% | 39.4% | 19.2% | 5.6% | 1.2% | 0.2% | | |
| Lactate: 1.5-2.1 | 0.0% | 0.1% | 0.9% | 5.8% | 17.3% | 21.6% | 78.4% | 74.2% | 53.8% | 30.2% | 14.4% | 5.2% | | |
| Lactate: 2.2-3.6 | 0.0% | 0.2% | 1.3% | 7.6% | 22.6% | 28.0% | 72.0% | 66.4% | 42.7% | 19.1% | 6.8% | 1.8% | | |
| Lactate: 3.7-24.0 | 0.0% | 0.1% | 1.5% | 10.8% | 35.6% | 44.8% | 55.2% | 45.8% | 15.8% | 2.7% | 0.2% | 0.0% | | |
| Baseline norepinephrine dose (µg/kg/min) | | | | | | | | | | | | |  |  |
| Norepinephrine: 0.00-0.00 | 0.1% | 1.3% | 8.1% | 28.4% | 55.1% | 62.0% | 38.0% | 31.2% | 10.1% | 1.2% | 0.1% | 0.0% | | |
| Norepinephrine: 0.01-0.10 | 1.7% | 5.4% | 14.0% | 31.4% | 52.7% | 58.5% | 41.5% | 35.9% | 16.4% | 4.8% | 0.9% | 0.2% | | |
| Norepinephrine: 0.11-0.21 | 1.1% | 4.0% | 11.6% | 27.9% | 48.8% | 54.8% | 45.2% | 39.4% | 19.5% | 6.3% | 1.6% | 0.2% | | |
| Norepinephrine: 0.22-0.39 | 0.1% | 0.5% | 2.6% | 11.3% | 30.6% | 37.2% | 62.8% | 56.0% | 31.2% | 13.0% | 4.5% | 1.2% | | |
| Norepinephrine: 0.40-2.40 | 0.0% | 0.0% | 0.2% | 3.4% | 15.4% | 20.1% | 79.9% | 74.7% | 51.5% | 29.5% | 14.3% | 5.2% | | |
| Baseline PaO_2_/FiO_2_ ratio (kPa)^b^ | | | | | | | | | | | | | |  |
| PaO_2_/FiO_2_ ratio: 4.5-11.0 | 0.1% | 0.4% | 2.9% | 14.9% | 40.0% | 47.8% | 52.2% | 44.3% | 16.8% | 2.6% | 0.3% | 0.0% | | |
| PaO_2_/FiO_2_ ratio: 11.0-14.1 | 0.0% | 0.1% | 1.0% | 8.4% | 29.0% | 36.2% | 63.8% | 56.1% | 27.0% | 7.3% | 1.2% | 0.1% | | |
| PaO_2_/FiO_2_ ratio: 14.1-17.4 | 0.0% | 0.1% | 1.0% | 9.0% | 30.6% | 38.3% | 61.7% | 53.5% | 24.7% | 6.0% | 0.9% | 0.1% | | |
| PaO_2_/FiO_2_ ratio: 17.4-22.2 | 0.8% | 3.7% | 12.5% | 33.2% | 59.2% | 66.0% | 34.0% | 27.6% | 8.8% | 1.0% | 0.1% | 0.0% | | |
| PaO_2_/FiO_2_ ratio: 22.2-157.6 | 0.0% | 0.2% | 2.1% | 12.7% | 35.7% | 43.0% | 57.0% | 49.2% | 22.1% | 5.2% | 0.9% | 0.1% | | |

SOFA score denotes Sequential Organ Failure Assessment score, PaO_2_ arterial partial pressure of oxygen, FiO_2_ fraction of inspired oxygen.

^a^ SOFA score ranges from 0-24, with sub-score from 0-4 for 6 organ system (respiration, coagulation, liver, cardiovascular, central nervous system, and renal), with higher aggregated scores indication higher degrees of organ failure.

^b^ Lower PaO_2_/FiO_2_ ratios indicate more severe pulmonary dysfunction.

## Table S9 Summarised effect measures for 90-day all-cause mortality in the sensitivity analysis using sceptic priors

| Group | n | Event probability, lower target | Event probability, higher target | Relative risk | Risk difference | Odds ratio |
| --- | --- | --- | --- | --- | --- | --- |
| All patients | 2888 | 42.9% (95% CrI: 38.4% to 47.6%) | 42.4% (95% CrI: 37.8% to 47.2%) | 1.01 (95% CrI: 0.94 to 1.09) | 0.5% (95% CrI:  -2.8% to 3.8%) | 1.02 (95% CrI: 0.89 to 1.17) |
| Baseline SOFA score^a^ | 2799 |  |  |  |  |  |
| SOFA score: 0-4 | 486 | 32.6% (95% CrI: 26.7% to 39.0%) | 32.0% (95% CrI: 26.2% to 38.3%) | 1.02 (95% CrI: 0.86 to 1.19) | 0.6% (95% CrI:  -4.7% to 5.6%) | 1.03 (95% CrI: 0.80 to 1.29) |
| SOFA score: 5-6 | 501 | 35.6% (95% CrI: 29.7% to 42.0%) | 35.8% (95% CrI: 29.8% to 42.4%) | 1.00 (95% CrI: 0.83 to 1.14) | 0.0% (95% CrI:  -6.6% to 4.7%) | 1.00 (95% CrI: 0.75 to 1.23) |
| SOFA score: 7-7 | 352 | 37.2% (95% CrI: 30.4% to 44.8%) | 34.3% (95% CrI: 27.4% to 41.3%) | 1.07 (95% CrI: 0.94 to 1.37) | 2.4% (95% CrI:  -2.4% to 11.2%) | 1.11 (95% CrI: 0.90 to 1.63) |
| SOFA score: 8-10 | 881 | 42.1% (95% CrI: 36.4% to 48.0%) | 41.5% (95% CrI: 36.0% to 47.2%) | 1.02 (95% CrI: 0.90 to 1.14) | 0.6% (95% CrI:  -4.3% to 5.3%) | 1.03 (95% CrI: 0.84 to 1.25) |
| SOFA score: 11-19 | 579 | 56.9% (95% CrI: 50.6% to 63.0%) | 55.6% (95% CrI: 49.3% to 61.7%) | 1.02 (95% CrI: 0.93 to 1.13) | 1.2% (95% CrI:  -4.2% to 7.0%) | 1.05 (95% CrI: 0.84 to 1.33) |
| Baseline lactate concentration [mM] | 2869 |  |  |  |  |  |
| Lactate concentration: 0.2-0.9 | 501 | 23.5% (95% CrI: 18.0% to 29.4%) | 25.4% (95% CrI: 20.2% to 31.9%) | 0.94 (95% CrI: 0.68 to 1.12) | -1.6% (95% CrI: -9.2% to 2.8%) | 0.92 (95% CrI: 0.60 to 1.17) |
| Lactate concentration: 1.0-1.4 | 631 | 38.2% (95% CrI: 32.1% to 44.4%) | 38.1% (95% CrI: 32.3% to 44.5%) | 1.00 (95% CrI: 0.86 to 1.15) | 0.1% (95% CrI:  -5.9% to 5.2%) | 1.01 (95% CrI: 0.78 to 1.25) |
| Lactate concentration: 1.5-2.1 | 577 | 41.7% (95% CrI: 35.2% to 48.7%) | 39.0% (95% CrI: 32.7% to 45.3%) | 1.06 (95% CrI: 0.93 to 1.28) | 2.5% (95% CrI:  -2.8% to 10.0%) | 1.11 (95% CrI: 0.89 to 1.52) |
| Lactate concentration: 2.2-3.6 | 576 | 44.8% (95% CrI: 38.5% to 51.2%) | 42.7% (95% CrI: 36.3% to 49.2%) | 1.04 (95% CrI: 0.92 to 1.23) | 1.9% (95% CrI:  -3.5% to 8.9%) | 1.08 (95% CrI: 0.87 to 1.44) |
| Lactate concentration: 3.7-24.0 | 584 | 61.4% (95% CrI: 55.0% to 67.5%) | 60.5% (95% CrI: 54.0% to 66.6%) | 1.01 (95% CrI: 0.92 to 1.12) | 0.9% (95% CrI:  -4.9% to 6.8%) | 1.04 (95% CrI: 0.81 to 1.33) |
| Baseline norepinephrine dose [µg/kg/min] | 2888 |  |  |  |  |  |
| Norepinephrine dose: 0.00-0.00 | 1373 | 38.1% (95% CrI: 33.0% to 43.5%) | 38.6% (95% CrI: 33.4% to 43.9%) | 0.99 (95% CrI: 0.87 to 1.10) | -0.4% (95% CrI: -5.1% to 3.7%) | 0.98 (95% CrI: 0.80 to 1.17) |
| Norepinephrine dose: 0.01-0.10 | 366 | 39.8% (95% CrI: 33.0% to 47.0%) | 40.2% (95% CrI: 33.4% to 47.1%) | 1.00 (95% CrI: 0.83 to 1.15) | -0.1% (95% CrI: -7.2% to 5.5%) | 1.00 (95% CrI: 0.74 to 1.26) |
| Norepinephrine dose: 0.11-0.21 | 372 | 39.5% (95% CrI: 32.5% to 46.8%) | 39.5% (95% CrI: 32.8% to 46.4%) | 1.00 (95% CrI: 0.84 to 1.16) | 0.1% (95% CrI:  -6.6% to 5.8%) | 1.01 (95% CrI: 0.76 to 1.28) |
| Norepinephrine dose: 0.22-0.39 | 348 | 49.5% (95% CrI: 42.3% to 57.1%) | 47.8% (95% CrI: 40.6% to 55.3%) | 1.03 (95% CrI: 0.91 to 1.22) | 1.4% (95% CrI:  -4.5% to 9.3%) | 1.06 (95% CrI: 0.83 to 1.46) |
| Norepinephrine dose: 0.40-2.40 | 429 | 51.8% (95% CrI: 45.0% to 59.3%) | 48.2% (95% CrI: 41.1% to 55.0%) | 1.06 (95% CrI: 0.95 to 1.29) | 3.1% (95% CrI:  -2.5% to 12.6%) | 1.13 (95% CrI: 0.90 to 1.66) |
| Baseline PaO_2_/FiO_2_ ratio (kPa)^b^ | 2876 |  |  |  |  |  |
| PaO_2_/FiO_2_ ratio: 4.5-11.0 | 565 | 45.9% (95% CrI: 39.8% to 52.2%) | 45.2% (95% CrI: 39.7% to 51.3%) | 1.01 (95% CrI: 0.91 to 1.12) | 0.6% (95% CrI:  -4.4% to 5.4%) | 1.02 (95% CrI: 0.84 to 1.24) |
| PaO_2_/FiO_2_ ratio: 11.0-14.1 | 584 | 46.5% (95% CrI: 40.4% to 52.9%) | 45.1% (95% CrI: 39.6% to 51.2%) | 1.03 (95% CrI: 0.93 to 1.16) | 1.2% (95% CrI:  -3.4% to 6.9%) | 1.05 (95% CrI: 0.87 to 1.32) |
| PaO_2_/FiO_2_ ratio: 14.1-17.4 | 574 | 46.4% (95% CrI: 40.5% to 52.8%) | 45.2% (95% CrI: 39.7% to 51.2%) | 1.02 (95% CrI: 0.93 to 1.15) | 1.1% (95% CrI:  -3.5% to 6.6%) | 1.05 (95% CrI: 0.87 to 1.30) |
| PaO_2_/FiO_2_ ratio: 17.4-22.2 | 577 | 41.8% (95% CrI: 35.2% to 48.1%) | 42.5% (95% CrI: 36.4% to 48.3%) | 0.99 (95% CrI: 0.85 to 1.10) | -0.5% (95% CrI: -6.6% to 4.0%) | 0.98 (95% CrI: 0.76 to 1.18) |
| PaO_2_/FiO_2_ ratio: 22.2-157.6 | 576 | 43.9% (95% CrI: 37.7% to 50.2%) | 43.1% (95% CrI: 37.1% to 48.8%) | 1.02 (95% CrI: 0.91 to 1.15) | 0.8% (95% CrI:  -4.0% to 6.0%) | 1.03 (95% CrI: 0.85 to 1.28) |

Posterior event probabilities, relative risks, risk differences and odd ratios for mortality. CrI denotes credibility interval, SOFA score denotes Sequential Organ Failure Assessment score, PaO_2_ arterial partial pressure of oxygen, FiO_2_ fraction of inspired oxygen, n the number of patients in each group (after excluding patients with missing data for one or more variables included in the analyses).

^a^ SOFA score ranges from 0-24, with sub-score from 0-4 for 6 organ system (respiration, coagulation, liver, cardiovascular, central nervous system, and renal), with higher aggregated scores indication higher degrees of organ failure.

^b^ Lower PaO_2_/FiO_2_ ratios indicate more severe pulmonary dysfunction.

## Table S10 Posterior probabilities of different effect sizes of 90-day all-cause mortality in the sensitivity analysis using sceptic priors

| Group | n | RR < 0.80 | RR < 0.85 | RR < 0.90 | RR < 0.95 | RR < 0.99 | RR < 1.00 | RR > 1.00 | RR > 1.01 | RR > 1.05 | RR > 1.11 | RR > 1.18 | RR > 1.25 |
| --- | --- | --- | --- | --- | --- | --- | --- | --- | --- | --- | --- | --- | --- |
| All patients | 2888 | 0.0% | 0.0% | 0.1% | 5.6% | 28.8% | 38.1% | 61.9% | 51.8% | 15.8% | 1.0% | 0.0% | 0.0% |
| Baseline SOFA score^a^ |  |  |  |  |  |  |  |  |  |  |  |  |  |
| SOFA score: 0-4 | 486 | 0.5% | 1.9% | 6.2% | 17.2% | 34.3% | 39.3% | 60.7% | 55.2% | 32.8% | 12.0% | 3.4% | 0.8% |
| SOFA score: 5-6 | 501 | 1.1% | 3.7% | 10.6% | 25.1% | 44.2% | 49.8% | 50.2% | 44.4% | 22.6% | 6.0% | 1.0% | 0.1% |
| SOFA score: 7-7 | 352 | 0.0% | 0.1% | 0.6% | 4.3% | 14.2% | 18.0% | 82.0% | 77.9% | 58.3% | 34.5% | 18.6% | 9.3% |
| SOFA score: 8-10 | 881 | 0.0% | 0.3% | 2.4% | 12.0% | 32.4% | 39.2% | 60.8% | 53.7% | 25.5% | 5.4% | 0.6% | 0.0% |
| SOFA score: 11-19 | 579 | 0.0% | 0.1% | 0.7% | 6.0% | 24.4% | 31.5% | 68.5% | 60.0% | 25.9% | 5.0% | 0.7% | 0.1% |
| Baseline lactate concentration (mM) | |  |  |  |  |  |  |  |  |  |  |  |  |
| Lactate: 0.2-0.9 | 501 | 14.9% | 24.6% | 38.0% | 54.2% | 68.3% | 72.0% | 28.0% | 24.4% | 12.2% | 3.3% | 0.6% | 0.1% |
| Lactate: 1.0-1.4 | 631 | 0.3% | 2.1% | 7.8% | 21.9% | 42.1% | 47.9% | 52.1% | 45.6% | 23.4% | 6.1% | 1.2% | 0.1% |
| Lactate: 1.5-2.1 | 577 | 0.0% | 0.0% | 0.7% | 4.5% | 15.2% | 19.1% | 80.9% | 76.5% | 55.2% | 29.8% | 13.0% | 4.4% |
| Lactate: 2.2-3.6 | 576 | 0.0% | 0.1% | 0.9% | 6.1% | 19.5% | 24.4% | 75.5% | 70.2% | 44.7% | 19.8% | 6.8% | 1.5% |
| Lactate: 3.7-24.0 | 584 | 0.0% | 0.1% | 0.9% | 7.8% | 28.5% | 36.7% | 63.3% | 54.0% | 20.7% | 3.4% | 0.3% | 0.0% |
| Baseline norepinephrine dose (µg/kg/min) | |  |  |  |  |  |  |  |  |  |  |  |  |
| Norepinephrine: 0.00-0.00 | 1373 | 0.1% | 1.0% | 6.2% | 24.9% | 50.3% | 57.2% | 42.8% | 35.8% | 12.9% | 1.7% | 0.1% | 0.0% |
| Norepinephrine: 0.01-0.10 | 366 | 1.1% | 3.8% | 10.6% | 26.0% | 45.8% | 51.7% | 48.3% | 42.5% | 20.6% | 5.6% | 1.3% | 0.3% |
| Norepinephrine: 0.11-0.21 | 372 | 0.7% | 2.9% | 8.8% | 22.8% | 42.3% | 47.9% | 52.1% | 46.3% | 24.1% | 7.2% | 1.6% | 0.3% |
| Norepinephrine: 0.22-0.39 | 348 | 0.0% | 0.3% | 1.7% | 8.6% | 24.9% | 30.9% | 69.1% | 62.5% | 36.9% | 14.9% | 5.0% | 1.2% |
| Norepinephrine: 0.40-2.40 | 429 | 0.0% | 0.0% | 0.2% | 2.4% | 12.1% | 16.3% | 83.7% | 78.6% | 55.8% | 30.7% | 14.2% | 4.9% |
| Baseline PaO_2_/FiO_2_ ratio (kPa)^b^ | | | | | | | | | | | | |  |
| PaO_2_/FiO_2_ ratio: 4.5-11.0 | 565 | 0.0% | 0.3% | 1.9% | 11.2% | 32.5% | 40.0% | 60.0% | 51.8% | 21.8% | 3.8% | 0.5% | 0.0% |
| PaO_2_/FiO_2_ ratio: 11.0-14.1 | 584 | 0.0% | 0.1% | 0.7% | 6.2% | 23.1% | 29.7% | 70.3% | 63.0% | 31.9% | 8.8% | 1.6% | 0.1% |
| PaO_2_/FiO_2_ ratio: 14.1-17.4 | 574 | 0.0% | 0.0% | 0.8% | 6.7% | 24.5% | 31.4% | 68.6% | 61.2% | 30.0% | 7.7% | 1.2% | 0.1% |
| PaO_2_/FiO_2_ ratio: 17.4-22.2 | 577 | 0.5% | 2.5% | 9.3% | 26.5% | 50.9% | 58.1% | 41.9% | 35.1% | 12.3% | 1.5% | 0.1% | 0.0% |
| PaO_2_/FiO_2_ ratio: 22.2-157.6 | 576 | 0.0% | 0.2% | 1.6% | 9.5% | 29.2% | 36.4% | 63.6% | 56.4% | 26.3% | 6.2% | 1.1% | 0.1% |

SOFA score denotes Sequential Organ Failure Assessment score, PaO_2_ arterial partial pressure of oxygen, FiO_2_ fraction of inspired oxygen.

^a^ SOFA score ranges from 0-24, with sub-score from 0-4 for 6 organ system (respiration, coagulation, liver, cardiovascular, central nervous system, and renal), with higher aggregated scores indication higher degrees of organ failure.

^b^ Lower PaO_2_/FiO_2_ ratios indicate more severe pulmonary dysfunction.

## Table S11 Comparison of treatment effects in baseline SOFA score-based subgroups

| Comparisons | SOFA score: 0-4 > row | SOFA score: 5-6 > row | SOFA score: 7-7 > row | SOFA score: 8-10 > row | SOFA score: 11-19 > row |
| --- | --- | --- | --- | --- | --- |
| Primary analysis | | | | | |
| SOFA score: 0-4 < col | - | 38.6% | 73.8% | 45.3% | 47.2% |
| SOFA score: 5-6 < col | 61.4% | - | 81.1% | 57.9% | 59.9% |
| SOFA score: 7-7 < col | 26.2% | 18.9% | - | 20.4% | 20.0% |
| SOFA score: 8-10 < col | 54.7% | 42.1% | 79.6% | - | 51.7% |
| SOFA score: 11-19 < col | 52.8% | 40.2% | 80.0% | 48.3% | - |
| Sensitivity analysis, evidence-based priors | | | | | |
| SOFA score: 0-4 < col | - | 39.9% | 72.6% | 47.9% | 51.6% |
| SOFA score: 5-6 < col | 60.1% | - | 79.3% | 59.2% | 63.3% |
| SOFA score: 7-7 < col | 27.4% | 20.7% | - | 23.1% | 24.8% |
| SOFA score: 8-10 < col | 52.1% | 40.8% | 76.9% | - | 54.4% |
| SOFA score: 11-19 < col | 48.4% | 36.7% | 75.2% | 45.6% | - |
| Sensitivity analysis, sceptic priors | | | | | |
| SOFA score: 0-4 < col | - | 40.2% | 70.0% | 46.7% | 49.0% |
| SOFA score: 5-6 < col | 59.8% | - | 76.9% | 56.6% | 58.9% |
| SOFA score: 7-7 < col | 30.0% | 23.1% | - | 24.7% | 25.0% |
| SOFA score: 8-10 < col | 53.3% | 43.4% | 75.3% | - | 51.7% |
| SOFA score: 11-19 < col | 51.0% | 41.1% | 75.0% | 48.3% | - |

The percentages denote the probability of the relative risk/odds ratio/risk difference for the treatment effect being higher or lower comparing the group specified in the columns (col) to the rows (row) or vice versa. SOFA score denotes Sequential Organ Failure Assessment score. SOFA score ranges from 0-24, with sub-score from 0-4 for 6 organ system (respiration, coagulation, liver, cardiovascular, central nervous system, and renal), with higher aggregated scores indication higher degrees of organ failure.

## Table S12 Comparison of treatment effects in baseline plasma lactate concentration-based subgroups

| Comparisons | Lactate concentration: 0.2-0.9 > row | Lactate concentration: 1.0-1.4 > row | Lactate concentration: 1.5-2.1 > row | Lactate concentration: 2.2-3.6 > row | Lactate concentration: 3.7-24.0 > row |
| --- | --- | --- | --- | --- | --- |
| Primary analysis | | | | | |
| Lactate concentration: 0.2-0.9 < col | - | 73.2% | 85.4% | 82.4% | 76.0% |
| Lactate concentration: 1.0-1.4 < col | 26.8% | - | 75.9% | 69.4% | 53.4% |
| Lactate concentration: 1.5-2.1 < col | 14.6% | 24.1% | - | 39.7% | 21.6% |
| Lactate concentration: 2.2-3.6 < col | 17.6% | 30.6% | 60.3% | - | 29.8% |
| Lactate concentration: 3.7-24.0 < col | 24.0% | 46.6% | 78.4% | 70.2% | - |
| Sensitivity analysis, evidence-based priors | | | | | |
| Lactate concentration: 0.2-0.9 < col | - | 74.8% | 86.6% | 84.3% | 79.2% |
| Lactate concentration: 1.0-1.4 < col | 25.2% | - | 75.7% | 70.0% | 57.4% |
| Lactate concentration: 1.5-2.1 < col | 13.5% | 24.3% | - | 41.0% | 24.7% |
| Lactate concentration: 2.2-3.6 < col | 15.7% | 30.0% | 59.0% | - | 32.0% |
| Lactate concentration: 3.7-24.0 < col | 20.8% | 42.6% | 75.3% | 68.0% | - |
| Sensitivity analysis, sceptic priors | | | | | |
| Lactate concentration: 0.2-0.9 < col | - | 72.3% | 83.6% | 81.1% | 75.4% |
| Lactate concentration: 1.0-1.4 < col | 27.7% | - | 74.4% | 67.7% | 54.7% |
| Lactate concentration: 1.5-2.1 < col | 16.4% | 25.6% | - | 41.3% | 25.1% |
| Lactate concentration: 2.2-3.6 < col | 18.9% | 32.3% | 58.7% | - | 32.5% |
| Lactate concentration: 3.7-24.0 < col | 24.6% | 45.3% | 74.9% | 67.5% | - |

The percentages denote the probability of the relative risk/odds ratio/risk difference for the treatment effect being higher or lower comparing the group specified in the columns (col) to the rows (row) or vice versa. Lactate concentrations are presented in mM.

## Table S13 Comparison of treatment effects in baseline norepinephrine dose-based subgroups

| Comparisons | Norepinephrine dose: 0.00-0.00 > row | Norepinephrine dose: 0.01-0.10 > row | Norepinephrine dose: 0.11-0.21 > row | Norepinephrine dose: 0.22-0.39 > row | Norepinephrine dose: 0.40-2.40 > row |
| --- | --- | --- | --- | --- | --- |
| Primary analysis | | | | | |
| Norepinephrine dose: 0.00-0.00 < col | - | 52.3% | 55.8% | 69.9% | 82.3% |
| Norepinephrine dose: 0.01-0.10 < col | 47.6% | - | 53.7% | 65.6% | 78.0% |
| Norepinephrine dose: 0.11-0.21 < col | 44.2% | 46.3% | - | 61.8% | 75.8% |
| Norepinephrine dose: 0.22-0.39 < col | 30.1% | 34.4% | 38.2% | - | 69.3% |
| Norepinephrine dose: 0.40-2.40 < col | 17.7% | 22.0% | 24.2% | 30.7% | - |
| Sensitivity analysis, evidence-based priors | | | | | |
| Norepinephrine dose: 0.00-0.00 < col | - | 50.5% | 54.0% | 68.6% | 81.4% |
| Norepinephrine dose: 0.01-0.10 < col | 49.5% | - | 53.0% | 65.9% | 78.2% |
| Norepinephrine dose: 0.11-0.21 < col | 46.0% | 47.0% | - | 62.5% | 75.9% |
| Norepinephrine dose: 0.22-0.39 < col | 31.4% | 34.1% | 37.5% | - | 67.3% |
| Norepinephrine dose: 0.40-2.40 < col | 18.6% | 21.8% | 24.1% | 32.7% | - |
| Sensitivity analysis, sceptic priors | | | | | |
| Norepinephrine dose: 0.00-0.00 < col | - | 52.5% | 56.5% | 69.6% | 80.8% |
| Norepinephrine dose: 0.01-0.10 < col | 47.5% | - | 53.6% | 64.9% | 76.6% |
| Norepinephrine dose: 0.11-0.21 < col | 43.5% | 46.4% | - | 61.7% | 73.8% |
| Norepinephrine dose: 0.22-0.39 < col | 30.4% | 35.1% | 38.4% | - | 67.0% |
| Norepinephrine dose: 0.40-2.40 < col | 19.2% | 23.4% | 26.2% | 33.0% | - |

The percentages denote the probability of the relative risk/odds ratio/risk difference for the treatment effect being higher or lower comparing the group specified in the columns (col) to the rows (row) or vice versa. Noradrenaline doses are presented in µg/kg/min.

## Table S14 Comparison of treatment effects in baseline PaO_2_/FiO_2_ ratio-based subgroups

| Comparisons | | | PaO_2_/FiO_2_ ratio: 4.5-11.0 > row | | PaO_2_/FiO_2_ ratio: 11.0-14.1 > row | | PaO_2_/FiO_2_ ratio: 14.1-17.4 > row | | PaO_2_/FiO_2_ ratio: 17.4-22.2 > row | PaO_2_/FiO_2_ ratio: 22.2-157.6 > row |
| --- | --- | --- | --- | --- | --- | --- | --- | --- | --- | --- |
| Primary analysis |  |  | |  | |  | |  | | |
| PaO_2_/FiO_2_ ratio: 4.5-11.0 < col | | | - | | 60.6% | | 58.9% | | 35.6% | 55.5% |
| PaO_2_/FiO_2_ ratio: 11.0-14.1 < col | | | 39.4% | | - | | 47.6% | | 27.8% | 44.7% |
| PaO_2_/FiO_2_ ratio: 14.1-17.4 < col | | | 41.1% | | 52.4% | | - | | 28.7% | 47.1% |
| PaO_2_/FiO_2_ ratio: 17.4-22.2 < col | | | 64.4% | | 72.2% | | 71.3% | | - | 69.1% |
| PaO_2_/FiO_2_ ratio: 22.2-157.6 < col | | | 44.5% | | 55.3% | | 52.9% | | 30.9% | - |
| Sensitivity analysis, evidence-based priors | | | | | | | | | | |
| PaO_2_/FiO_2_ ratio: 4.5-11.0 < col | | | - | | 60.3% | | 58.5% | | 34.6% | 54.7% |
| PaO_2_/FiO_2_ ratio: 11.0-14.1 < col | | | 39.7% | | - | | 48.3% | | 26.9% | 44.2% |
| PaO_2_/FiO_2_ ratio: 14.1-17.4 < col | | | 41.5% | | 51.7% | | - | | 28.3% | 46.0% |
| PaO_2_/FiO_2_ ratio: 17.4-22.2 < col | | | 65.4% | | 73.1% | | 71.7% | | - | 69.8% |
| PaO_2_/FiO_2_ ratio: 22.2-157.6 < col | | | 45.3% | | 55.8% | | 54.0% | | 30.2% | - |
| Sensitivity analysis, sceptic priors | | | | | | | | | | |
| PaO_2_/FiO_2_ ratio: 4.5-11.0 < col | | | - | | 59.8% | | 58.4% | | 35.4% | 54.6% |
| PaO_2_/FiO_2_ ratio: 11.0-14.1 < col | | | 40.2% | | - | | 48.1% | | 27.8% | 44.4% |
| PaO_2_/FiO_2_ ratio: 14.1-17.4 < col | | | 41.6% | | 51.9% | | - | | 29.1% | 45.9% |
| PaO_2_/FiO_2_ ratio: 17.4-22.2 < col | | | 64.6% | | 72.2% | | 70.9% | | - | 68.5% |
| PaO_2_/FiO_2_ ratio: 22.2-157.6 < col | | | 45.4% | | 55.6% | | 54.1% | | 31.5% | - |

The percentages denote the probability of the relative risk/odds ratio/risk difference for the treatment effect being higher or lower comparing the group specified in the columns (col) to the rows (row) or vice versa. PaO_2_/FiO_2_ ratio denotes partial pressure of arterial oxygen to fraction of inspired oxygen ratio. Lower PaO_2_/FiO_2_ ratios indicate more severe pulmonary dysfunction. PaO_2_/FiO_2_ ratios are presented in kPa.

## Fig. S1a Posterior probability distribution of the adjusted risk difference for 90-day all-cause mortality in the primary analysis using weakly informative priors


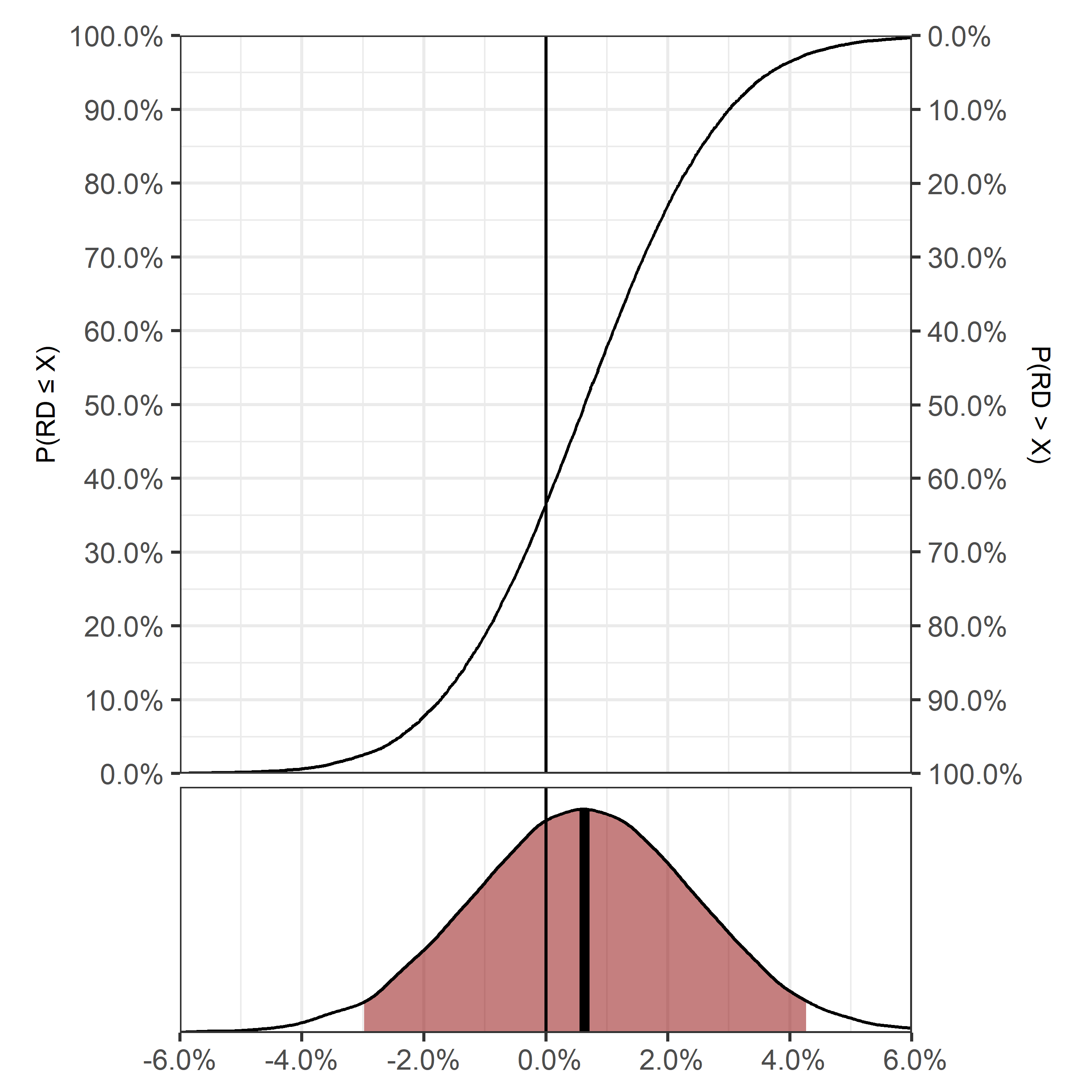


*Upper part*: cumulative posterior probability distribution for the adjusted risk difference (RD). P(RD ≤ X) is the probability that the RD is smaller or equal to any given value specified on the X-axis, being “X”; P(RD > X) is the probability that the RD is larger than any given value specified on the X-axis, being “X”. A negative RD indicates benefit from the lower oxygenation target; a positive RD indicates benefit of the higher oxygenation target. *Lower part*: full posterior probability distribution; full vertical line = median value; coloured area = 95% credibility interval.

## Fig. S1b Posterior probability distribution of the adjusted odds ratio for 90-day all-cause mortality in the primary analysis using weakly informative priors


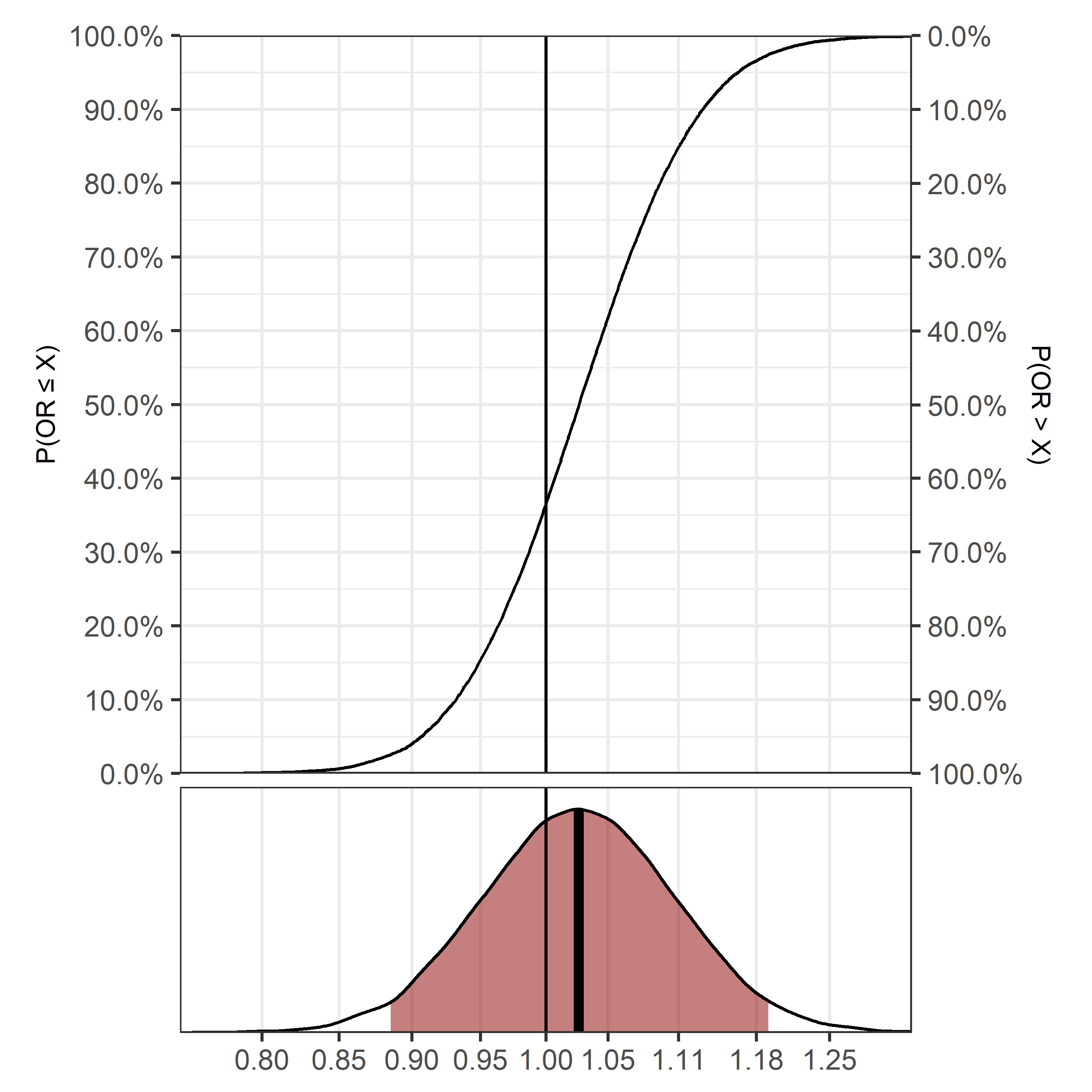


*Upper part*: cumulative posterior probability distribution for the adjusted odds ratio (OR). P(OR ≤ X) is the probability that the OR is smaller or equal to any given value specified on the X-axis, being “X”; P(OR > X) is the probability that the OR is larger than any given value specified on the X-axis, being “X”. An OR < 1 indicates benefit from the lower oxygenation target; an OR > 1 indicates benefit of the higher oxygenation target. *Lower part*: full posterior probability distribution; full vertical line = median value; coloured area = 95% credibility interval.

## Fig. S2a Posterior probability distribution of the adjusted relative risk for 90-day all-cause mortality in the sensitivity analysis using evidence-based priors


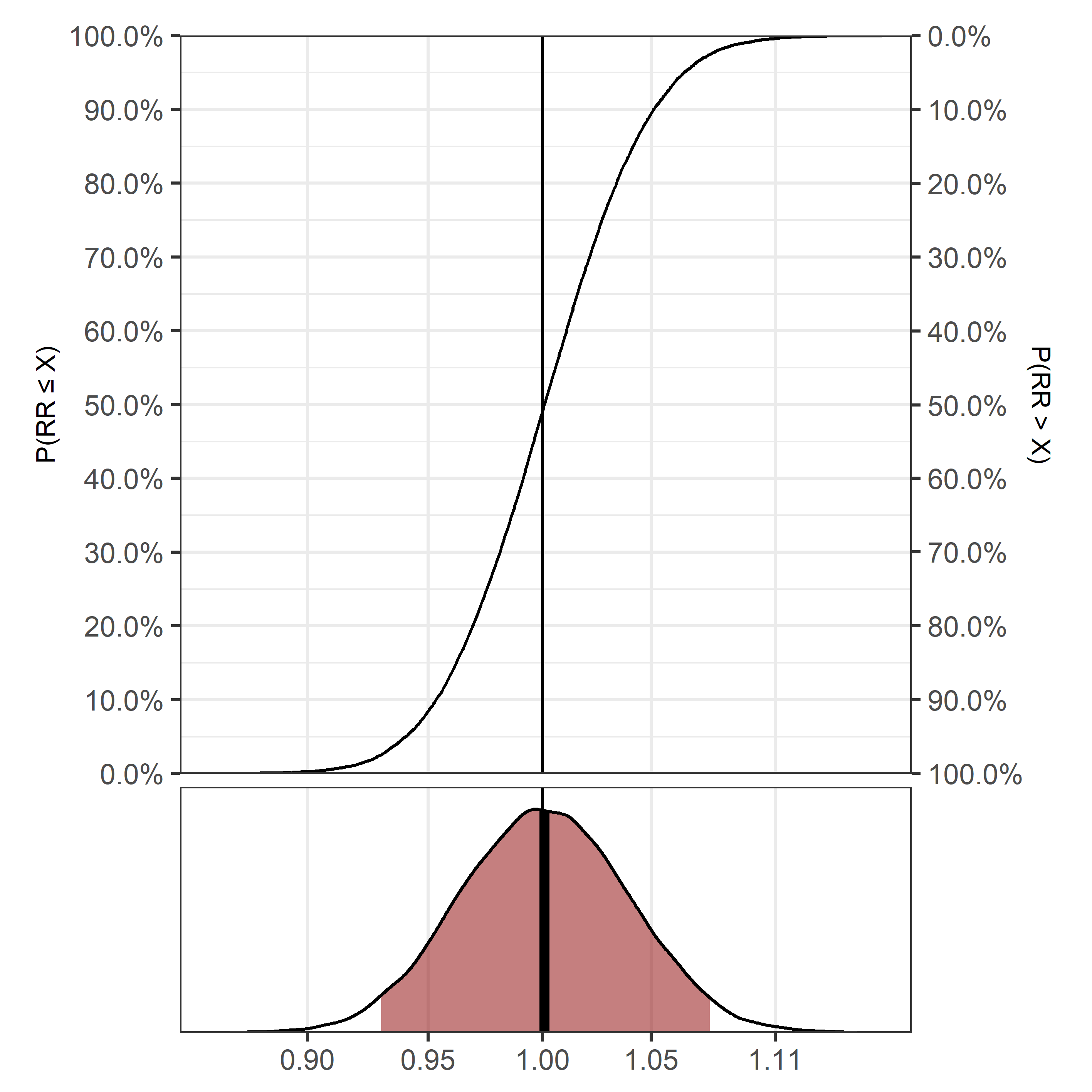


*Upper part*: cumulative posterior probability distribution for the adjusted relative risk (RR). P(RR ≤ X) is the probability that the RR is smaller or equal to any given value specified on the X-axis, being “X”; P(RR > X) is the probability that the RR is larger than any given value specified on the X-axis, being “X”. An RR < 1 indicates benefit from the lower oxygenation target; an RR > 1 indicates benefit of the higher oxygenation target. *Lower part*: full posterior probability distribution; full vertical line = median value; coloured area = 95% credibility interval.

## Fig. S2b Posterior probability distribution of the adjusted risk difference for 90-day all-cause mortality in the sensitivity analysis using evidence-based priors


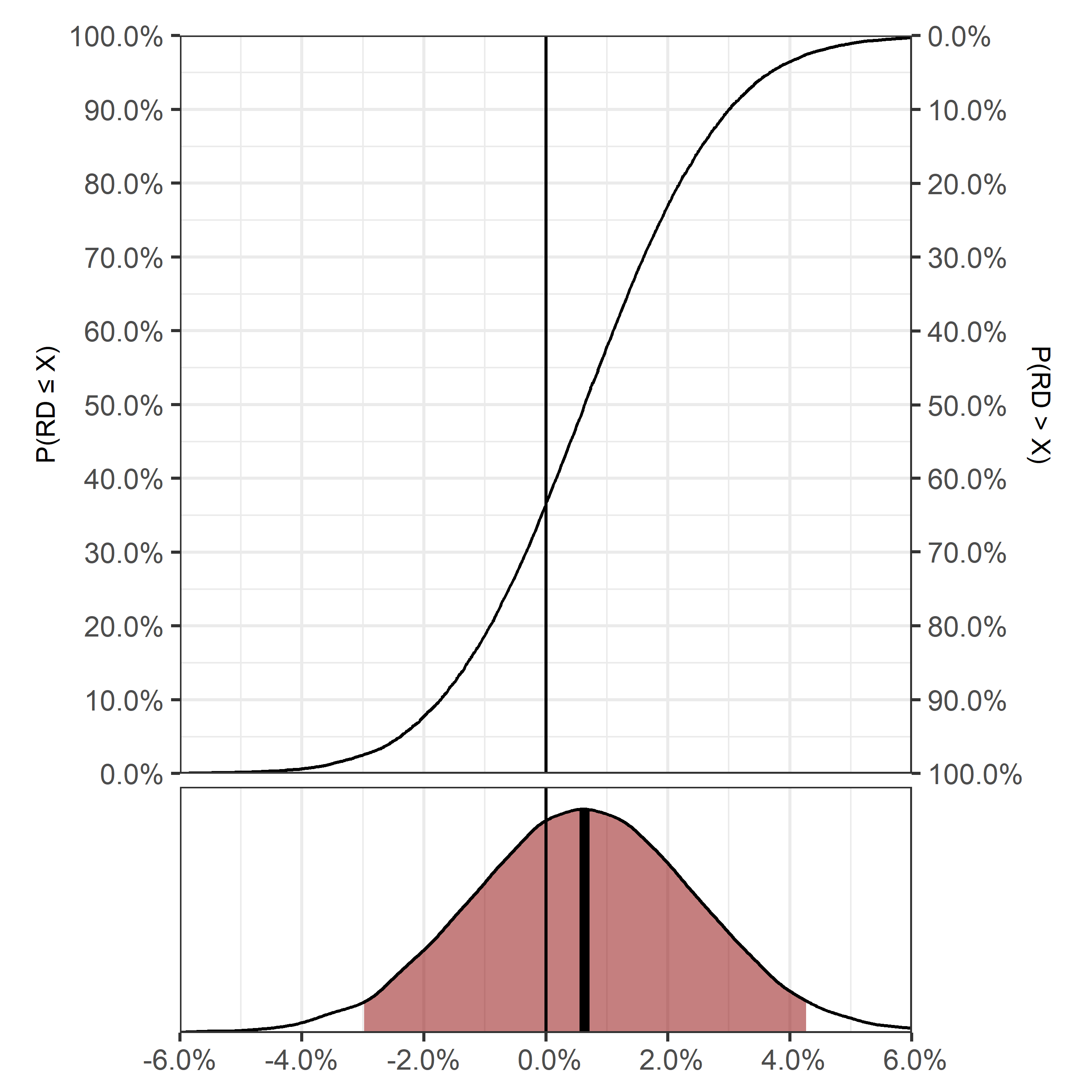


*Upper part*: cumulative posterior probability distribution for the adjusted risk difference (RD). P(RD ≤ X) is the probability that the RD is smaller or equal to any given value specified on the X-axis, being “X”; P(RD > X) is the probability that the RD is larger than any given value specified on the X-axis, being “X”. A negative RD indicates benefit from the lower oxygenation target; a positive RD indicates benefit of the higher oxygenation target. *Lower part*: full posterior probability distribution; full vertical line = median value; coloured area = 95% credibility interval.

## Fig. S2c Posterior probability distribution of the adjusted odds ratio for 90-day all-cause mortality in the sensitivity analysis using evidence-based priors


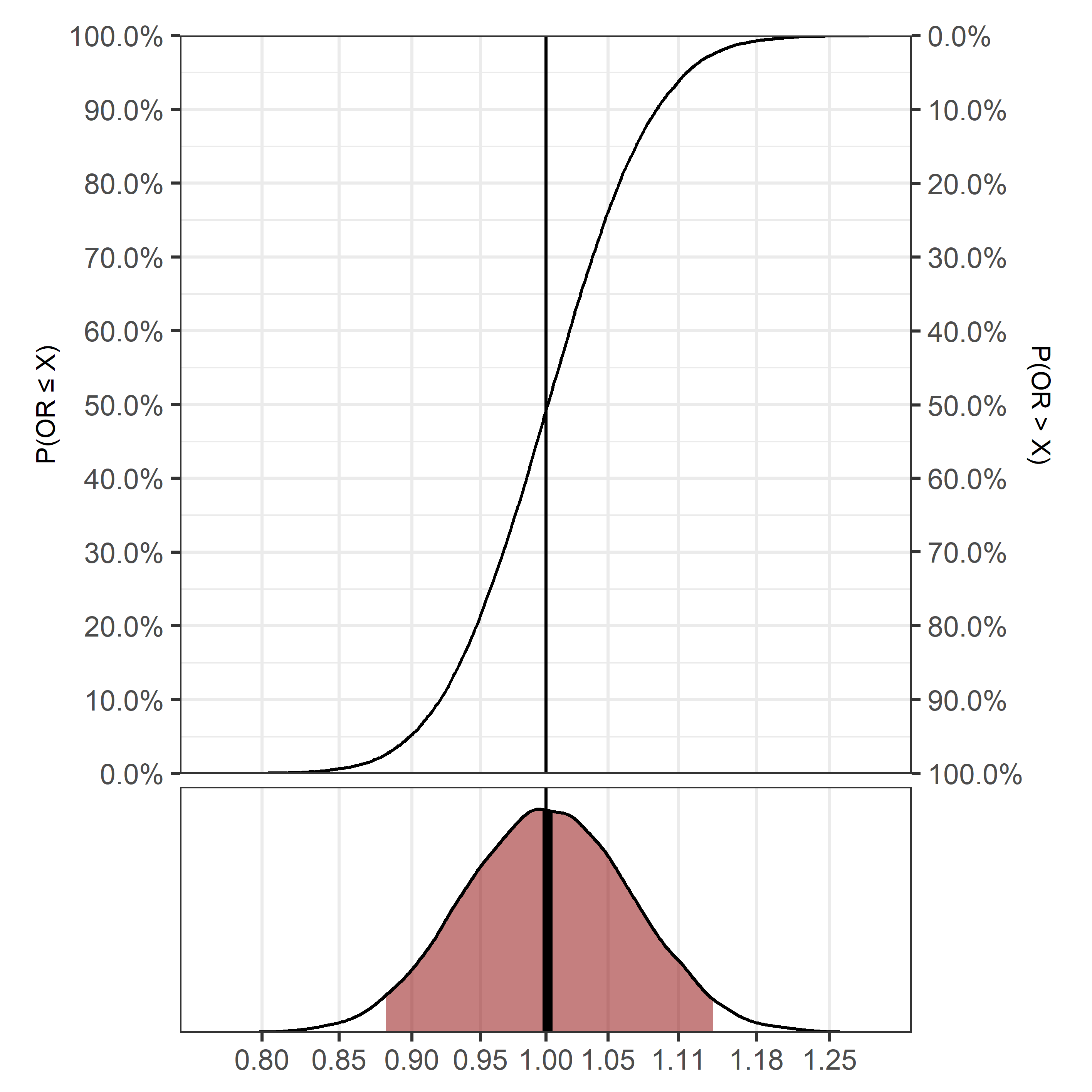


*Upper part*: cumulative posterior probability distribution for the adjusted odds ratio (OR). P(OR ≤ X) is the probability that the OR is smaller or equal to any given value specified on the X-axis, being “X”; P(OR > X) is the probability that the OR is larger than any given value specified on the X-axis, being “X”. An OR < 1 indicates benefit from the lower oxygenation target; an OR > 1 indicates benefit of the higher oxygenation target. *Lower part*: full posterior probability distribution; full vertical line = median value; coloured area = 95% credibility interval.

## Fig. S3a Posterior probability distribution of the adjusted relative risk for 90-day all-cause mortality in the sensitivity analysis using sceptic priors


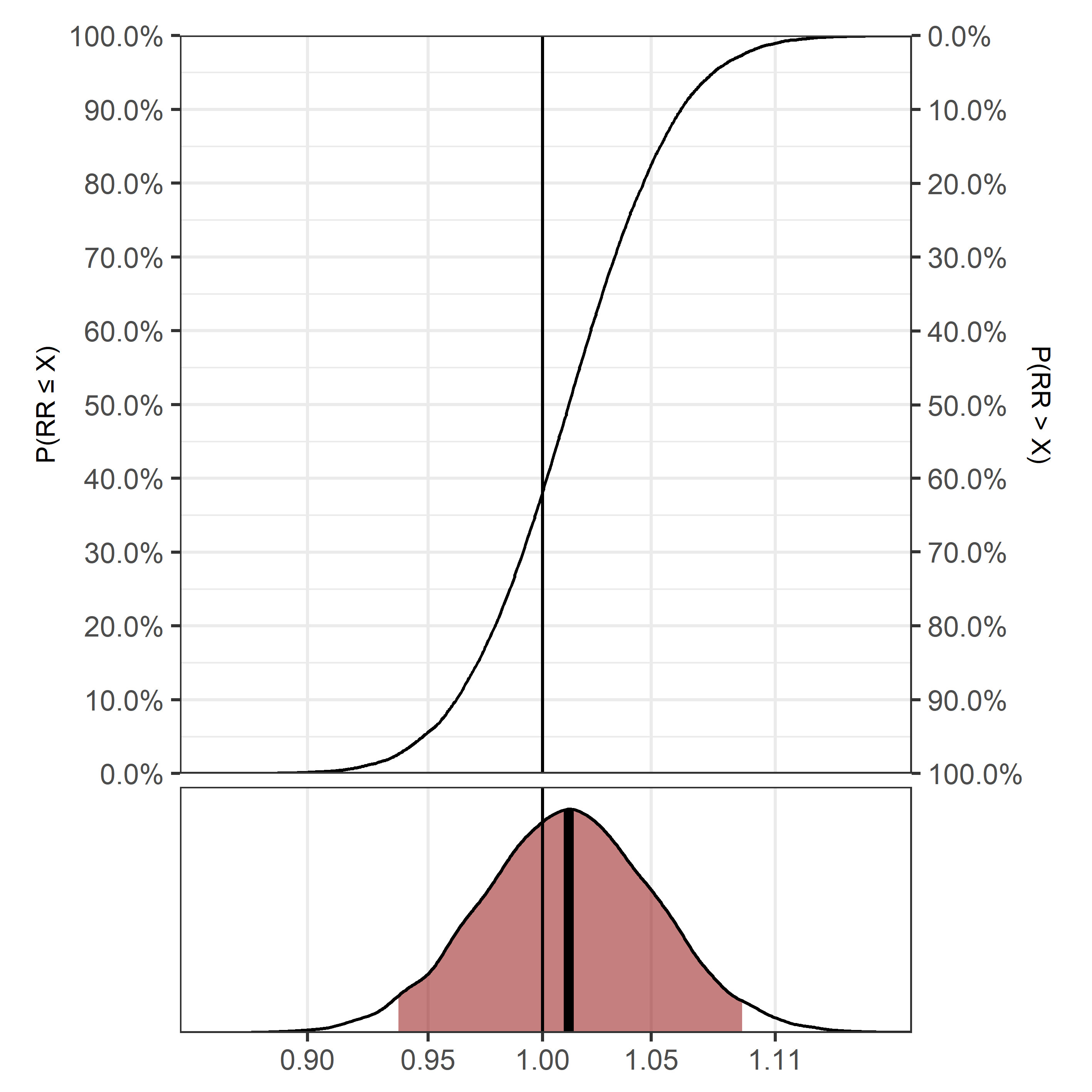


*Upper part*: cumulative posterior probability distribution for the adjusted relative risk (RR). P(RR ≤ X) is the probability that the RR is smaller or equal to any given value specified on the X-axis, being “X”; P(RR > X) is the probability that the RR is larger than any given value specified on the X-axis, being “X”. An RR < 1 indicates benefit from the lower oxygenation target; an RR > 1 indicates benefit of the higher oxygenation target. *Lower part*: full posterior probability distribution; full vertical line = median value; coloured area = 95% credibility interval.

## Fig. S3b Posterior probability distribution of the adjusted risk difference for 90-day all-cause mortality in the sensitivity analysis using sceptic priors


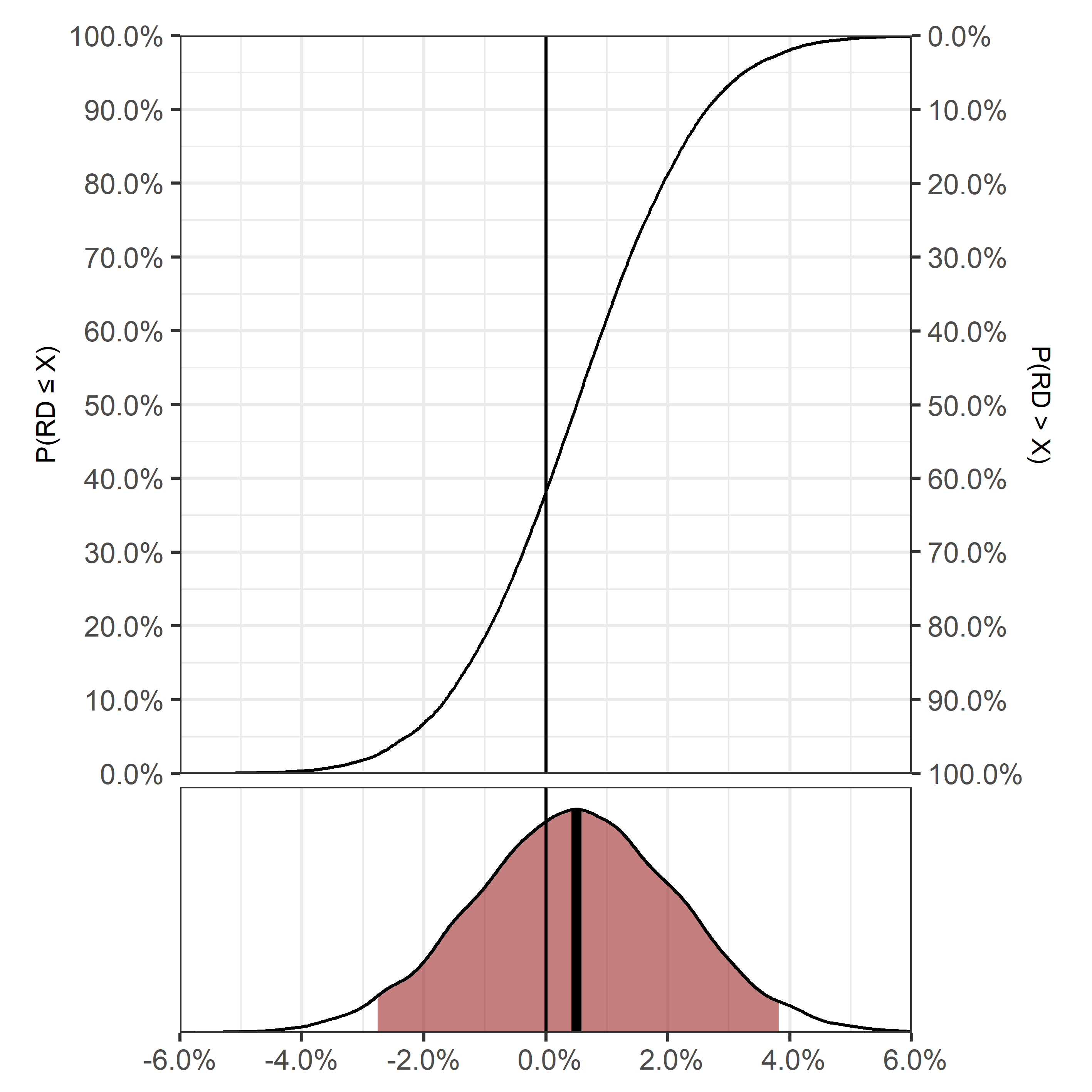


*Upper part*: cumulative posterior probability distribution for the adjusted risk difference (RD). P(RD ≤ X) is the probability that the RD is smaller or equal to any given value specified on the X-axis, being “X”; P(RD > X) is the probability that the RD is larger than any given value specified on the X-axis, being “X”. A negative RD indicates benefit from the lower oxygenation target; a positive RD indicates benefit of the higher oxygenation target. *Lower part*: full posterior probability distribution; full vertical line = median value; coloured area = 95% credibility interval.

## Fig. S3c Posterior probability distribution of the adjusted odds ratio for 90-day all-cause mortality in the sensitivity analysis using sceptic priors


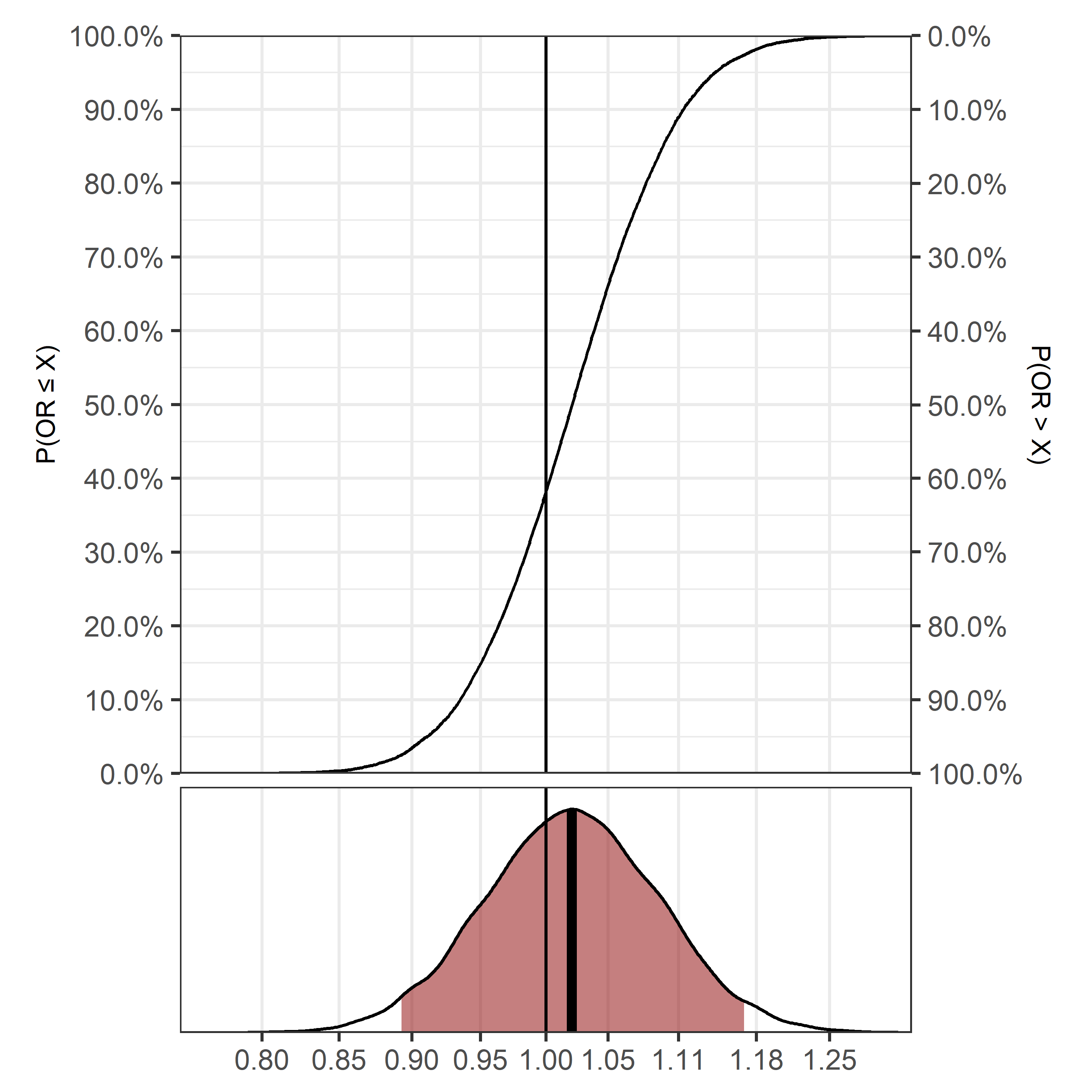


*Upper part*: cumulative posterior probability distribution for the adjusted odds ratio (OR). P(OR ≤ X) is the probability that the OR is smaller or equal to any given value specified on the X-axis, being “X”; P(OR > X) is the probability that the OR is larger than any given value specified on the X-axis, being “X”. An OR < 1 indicates benefit from the lower oxygenation target; an OR > 1 indicates benefit of the higher oxygenation target. *Lower part*: full posterior probability distribution; full vertical line = median value; coloured area = 95% credibility interval.

## Fig. S4a Posterior probability distributions of the adjusted risk differences of the treatment effect on 90-day all-cause mortality in the four sets of subgroups in the primary analysis using weakly informative priors


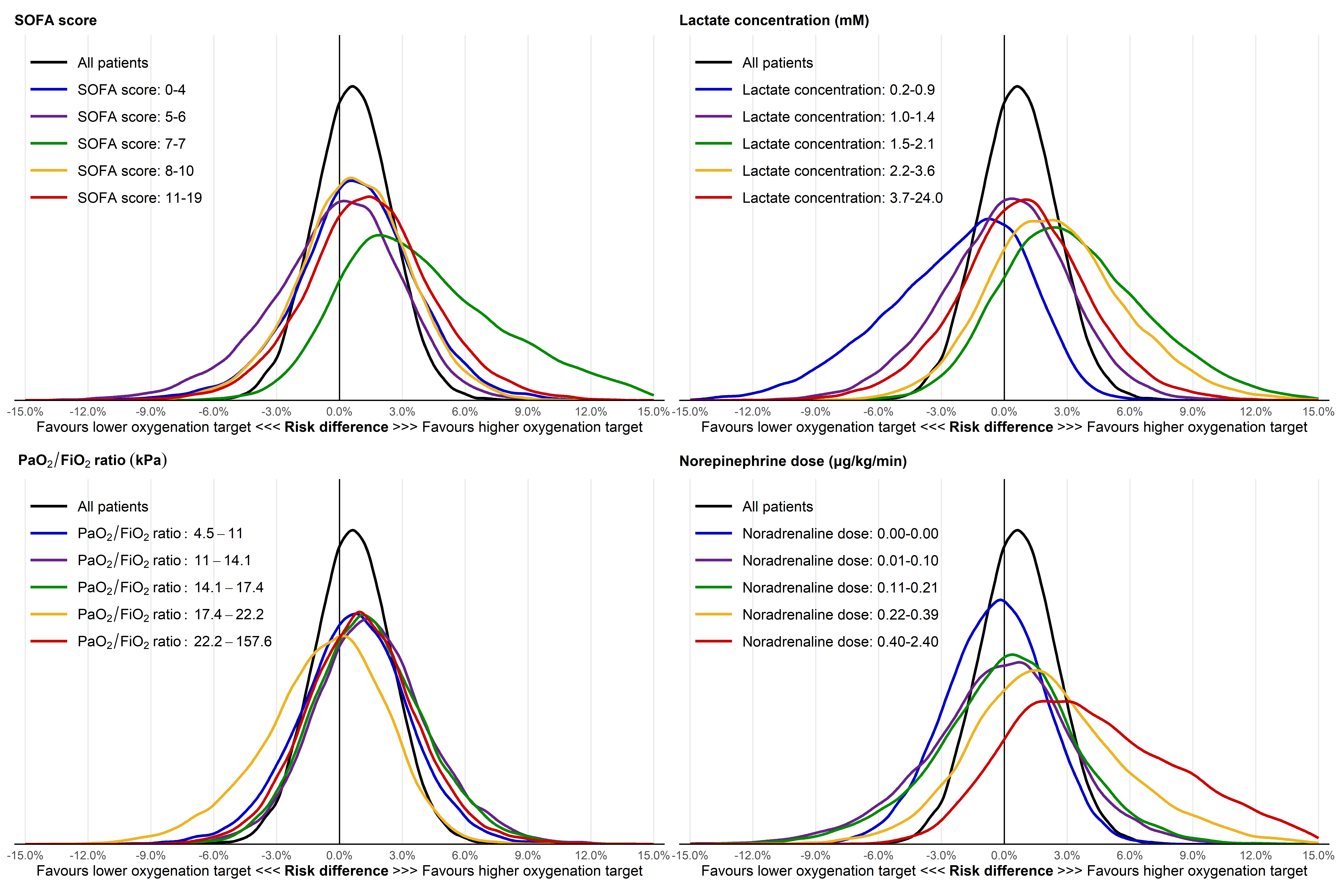


The posterior probability distributions of the adjusted risk differences (RD) in each subgroup from the subgroup-based models are displayed together with the posterior distribution from the analysis of all patients using weakly informative priors. A negative RD indicates benefit from the lower oxygenation target; a positive RD indicates benefit of the higher oxygenation target. SOFA score denotes Sequential Organ Failure Assessment score, PaO_2_ arterial partial pressure of oxygen, FiO_2_ fraction of inspired.

## Fig. S4b Posterior probability distributions of the adjusted odds ratios of the treatment effect on 90-day all-cause mortality in the four sets of subgroups in the primary analysis using weakly informative priors


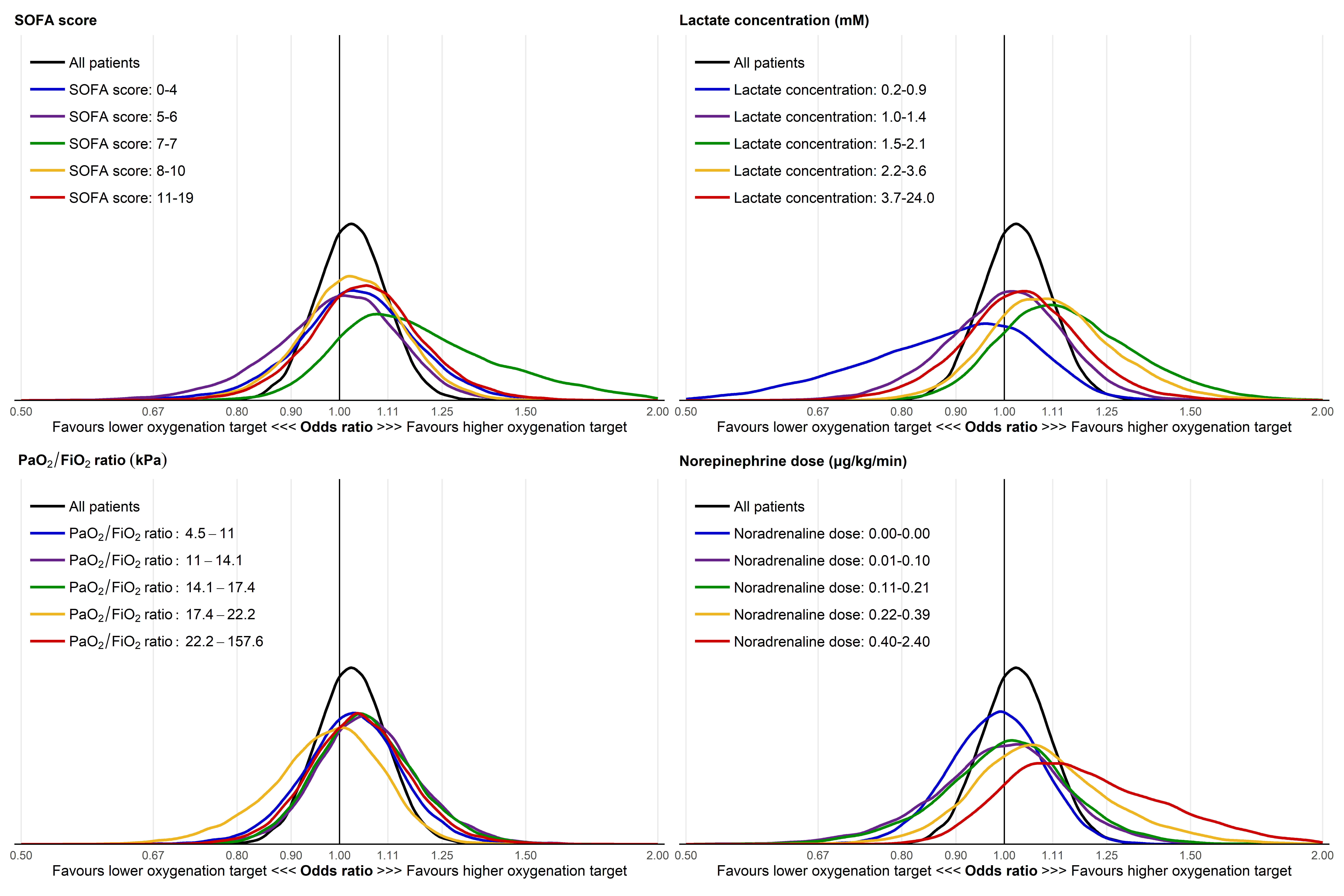


The posterior probability distributions of the adjusted odds ratios (OR) in each subgroup from the subgroup-based models are displayed together with the posterior distribution from the analysis of all patients using weakly informative priors. An OR < 1 indicates benefit from the lower oxygenation target; an OR > 1 indicates benefit of the higher oxygenation target. SOFA score denotes Sequential Organ Failure Assessment score, PaO_2_ arterial partial pressure of oxygen, FiO_2_ fraction of inspired oxygen.

## Fig. S5a Posterior probability distributions of the adjusted relative risks of the treatment effect on 90-day all-cause mortality in the four sets of subgroups in the sensitivity analysis using evidence-based priors


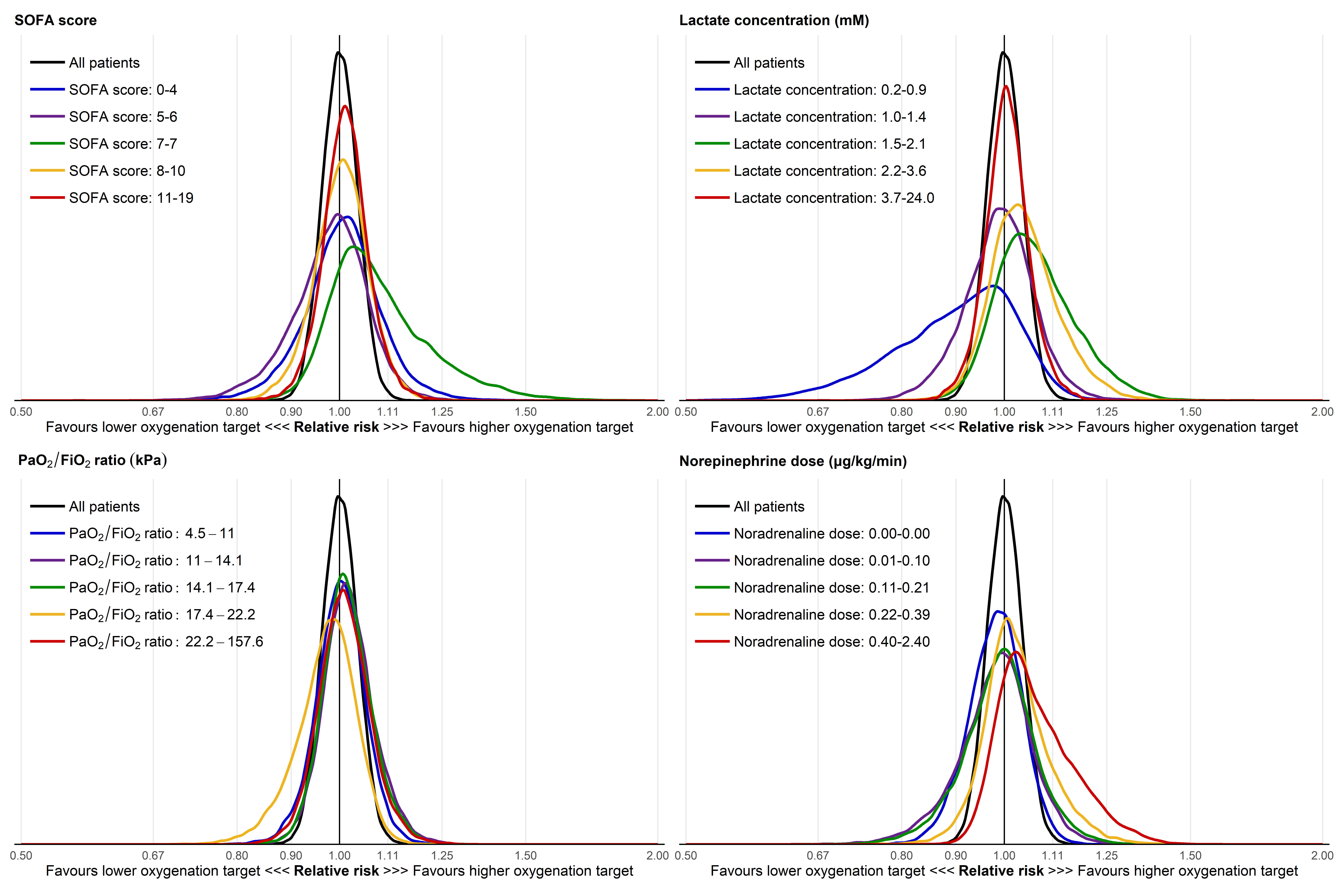


The posterior probability distributions of the adjusted relative risks (RR) in each subgroup from the subgroup-based models are displayed together with the posterior distribution from the analysis of all patients using evidence-based priors. An RR < 1 indicates benefit from the lower oxygenation target; an RR > 1 indicates benefit of the higher oxygenation target. SOFA score denotes Sequential Organ Failure Assessment score, PaO_2_ arterial partial pressure of oxygen, FiO_2_ fraction of inspired oxygen.

## Fig. S5b Posterior probability distributions of the adjusted risk differences of the treatment effect on 90-day all-cause mortality in the four sets of subgroups in the sensitivity analysis using evidence-based priors


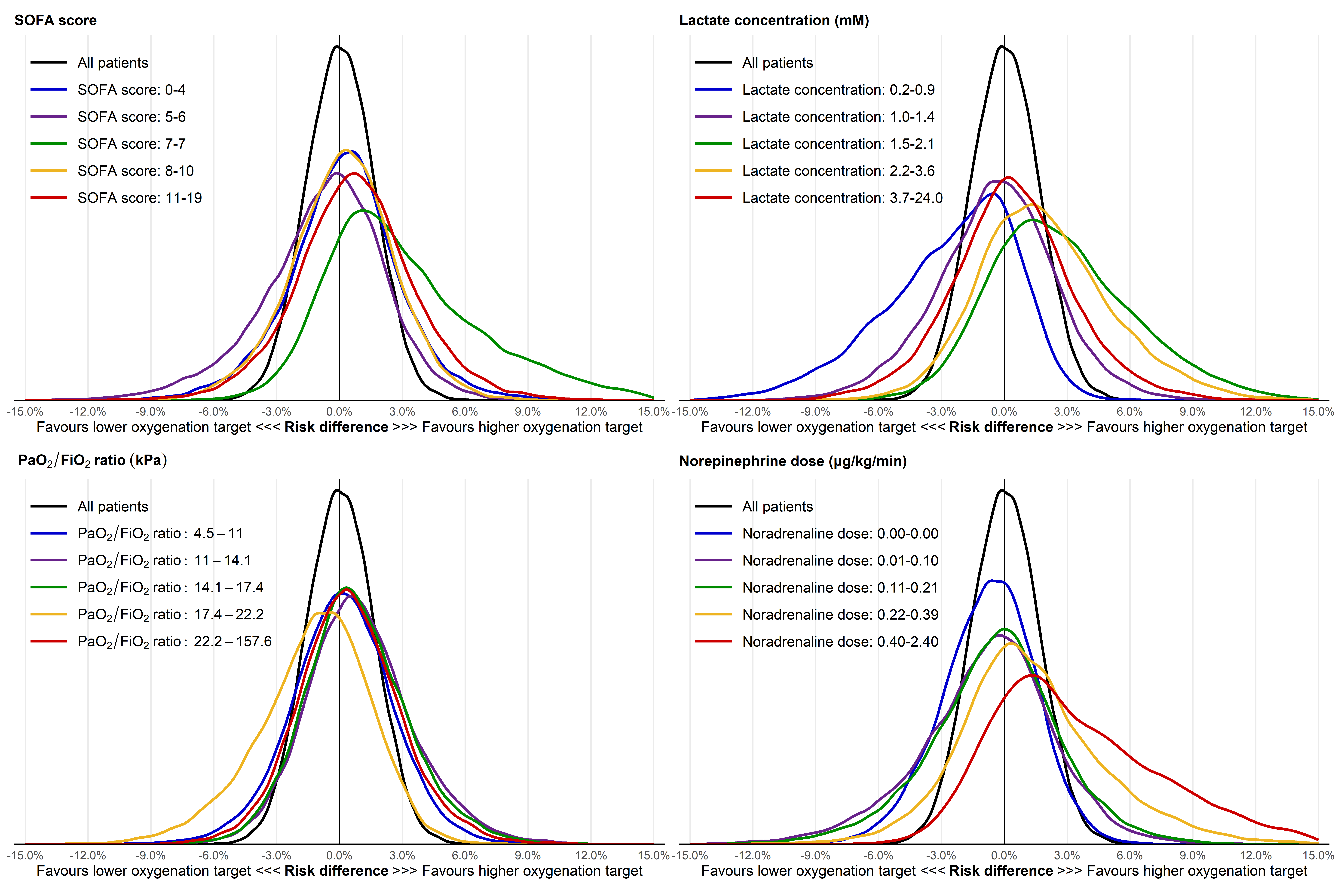


The posterior probability distributions of the adjusted risk differences (RD) in each subgroup from the subgroup-based models are displayed together with the posterior distribution from the analysis of all patients using evidence-based priors. A negative RR indicates benefit from the lower oxygenation target; a positive RR indicates benefit of the higher oxygenation target. SOFA score denotes Sequential Organ Failure Assessment score, , PaO_2_ arterial partial pressure of oxygen, FiO_2_ fraction of inspired oxygen.

## Fig. S5c Posterior probability distributions of the adjusted odds ratios of the treatment effect on 90-day all-cause mortality in the four sets of subgroup in the sensitivity analysis using evidence-based priors


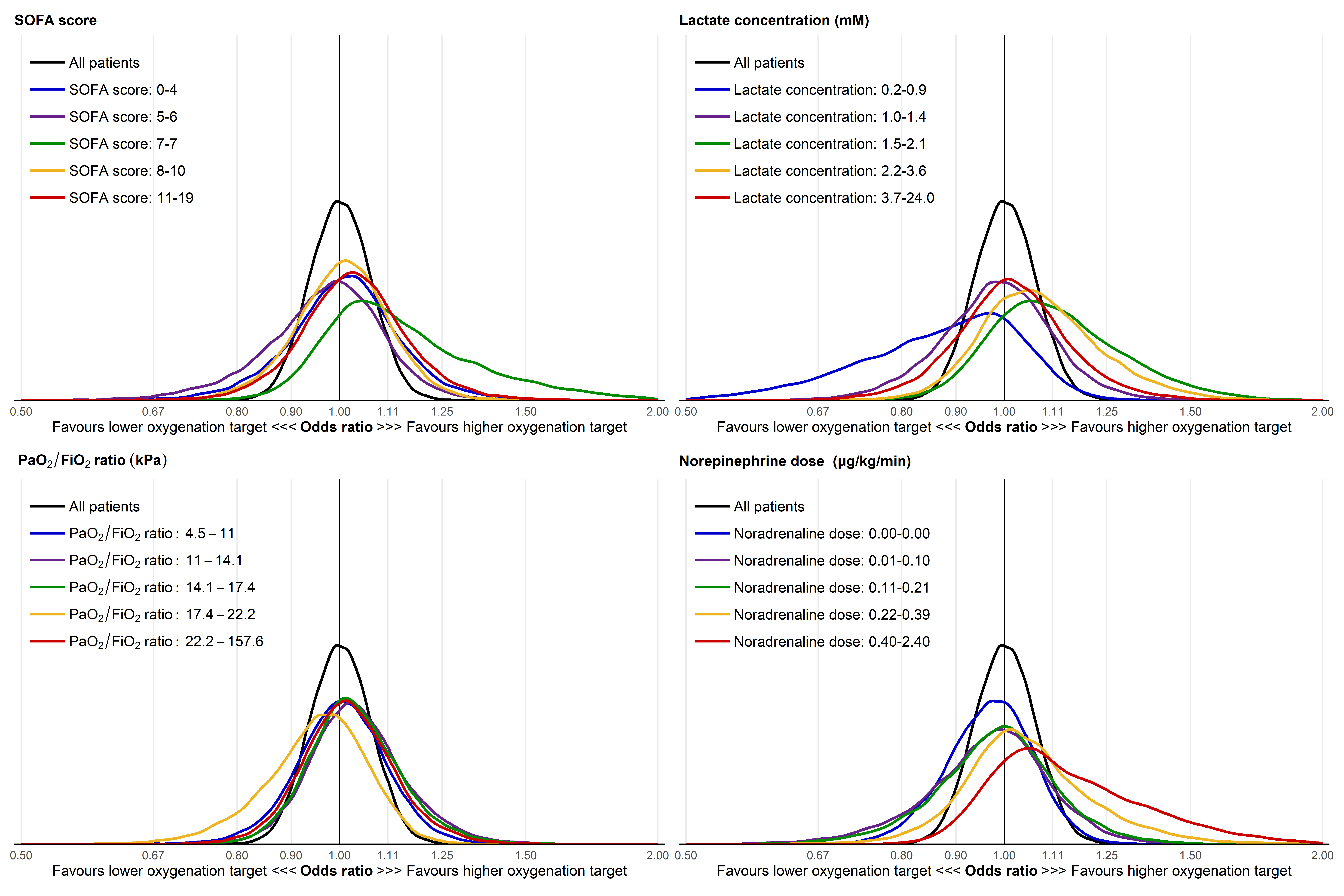


The posterior probability distributions of the adjusted odds ratios (OR) in each subgroup from the subgroup-based models are displayed together with the posterior distribution from the analysis of all patients using evidence-based priors. An OR < 1 indicated benefit from the lower oxygenation target; an OR > 1 indicates benefit of the higher oxygenation target. SOFA score denotes Sequential Organ Failure Assessment score, PaO_2_ arterial partial pressure of oxygen, FiO_2_ fraction of inspired oxygen.

## Fig. S6a Posterior probability distributions of the adjusted relative risks of the treatment effect on 90-day all-cause mortality in the four sets of subgroups in the sensitivity analysis using sceptic priors


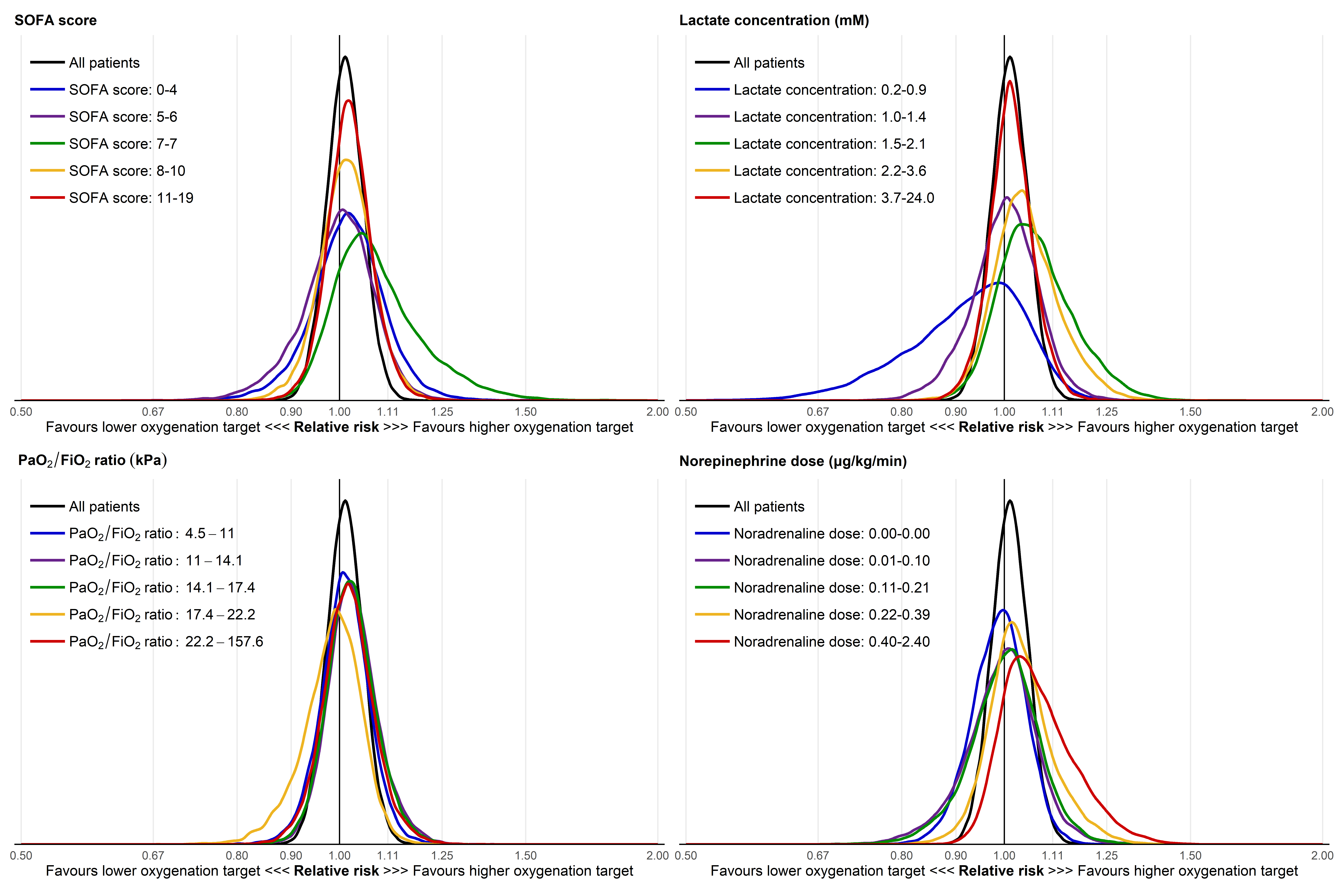


The posterior probability distributions of the adjusted relative risks (RR) in each subgroup from the subgroup-based models are displayed together with the posterior distribution from the analysis of all patients using sceptic priors. An RR < 1 indicated benefit from the lower oxygenation target; an RR > 1 indicates benefit of the higher oxygenation target. SOFA score denotes Sequential Organ Failure Assessment score, PaO_2_ arterial partial pressure of oxygen, FiO_2_ fraction of inspired oxygen.

## Fig. S6b Posterior probability distributions of the adjusted risk differences of the treatment effect on 90-day all-cause mortality in the four sets of subgroups in the sensitivity analysis using sceptic priors


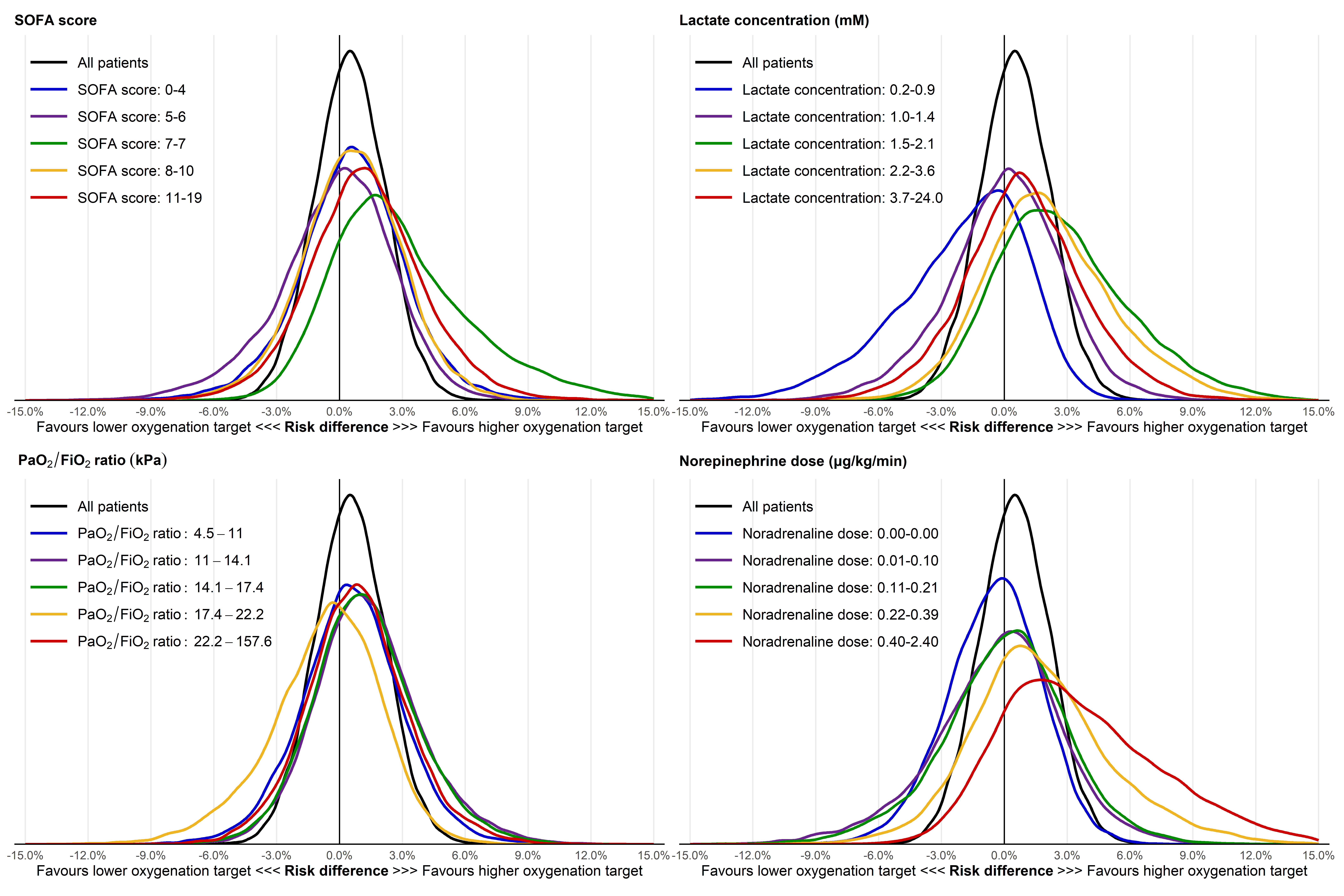


The posterior probability distributions of the adjusted risk differences (RD) in each subgroup from the subgroup-based models are displayed together with the posterior distribution from the analysis of all patients using sceptic priors. A negative RD indicated benefit from the lower oxygenation target; a positive RD indicates benefit of the higher oxygenation target. SOFA score denotes Sequential Organ Failure Assessment score, PaO_2_ arterial partial pressure of oxygen, FiO_2_ fraction of inspired oxygen.

## Fig. S6c Posterior probability distributions of the adjusted odds ratios of the treatment effect on 90-day all-cause mortality in the four sets of subgroups in the sensitivity analysis using sceptic priors


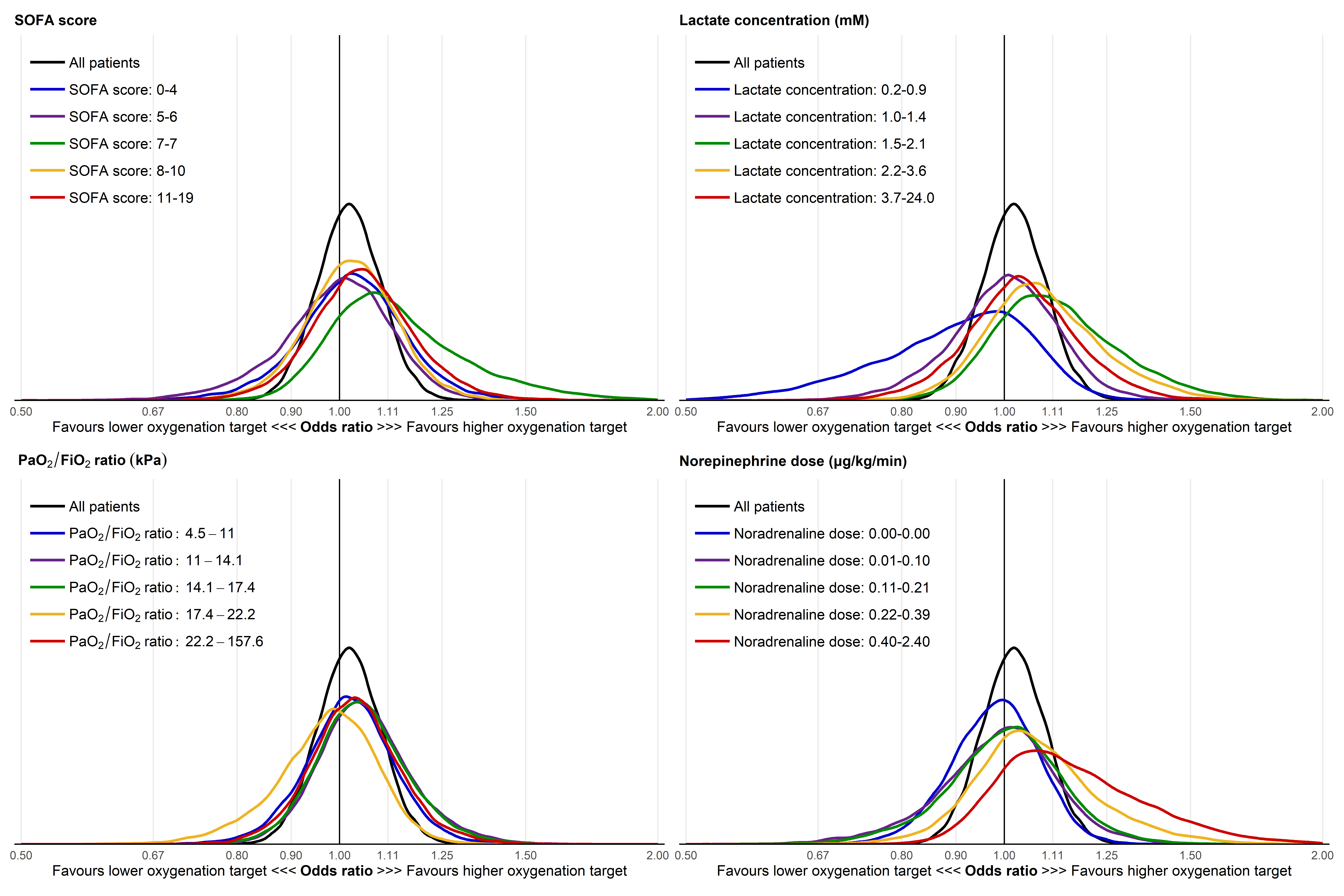


The posterior probability distributions of the adjusted odds ratios (OR) in each subgroup from the subgroup-based models are displayed together with the posterior distribution from the analysis of all patients using sceptic priors. A negative OR indicated benefit from the lower oxygenation target; a positive OR indicates benefit of the higher oxygenation target. SOFA score denotes Sequential Organ Failure Assessment score, PaO_2_ arterial partial pressure of oxygen, FiO_2_ fraction of inspired oxygen.

## Fig. S7a Conditional effects plot for 90-day all-cause mortality in the sensitivity analysis using evidence-based priors


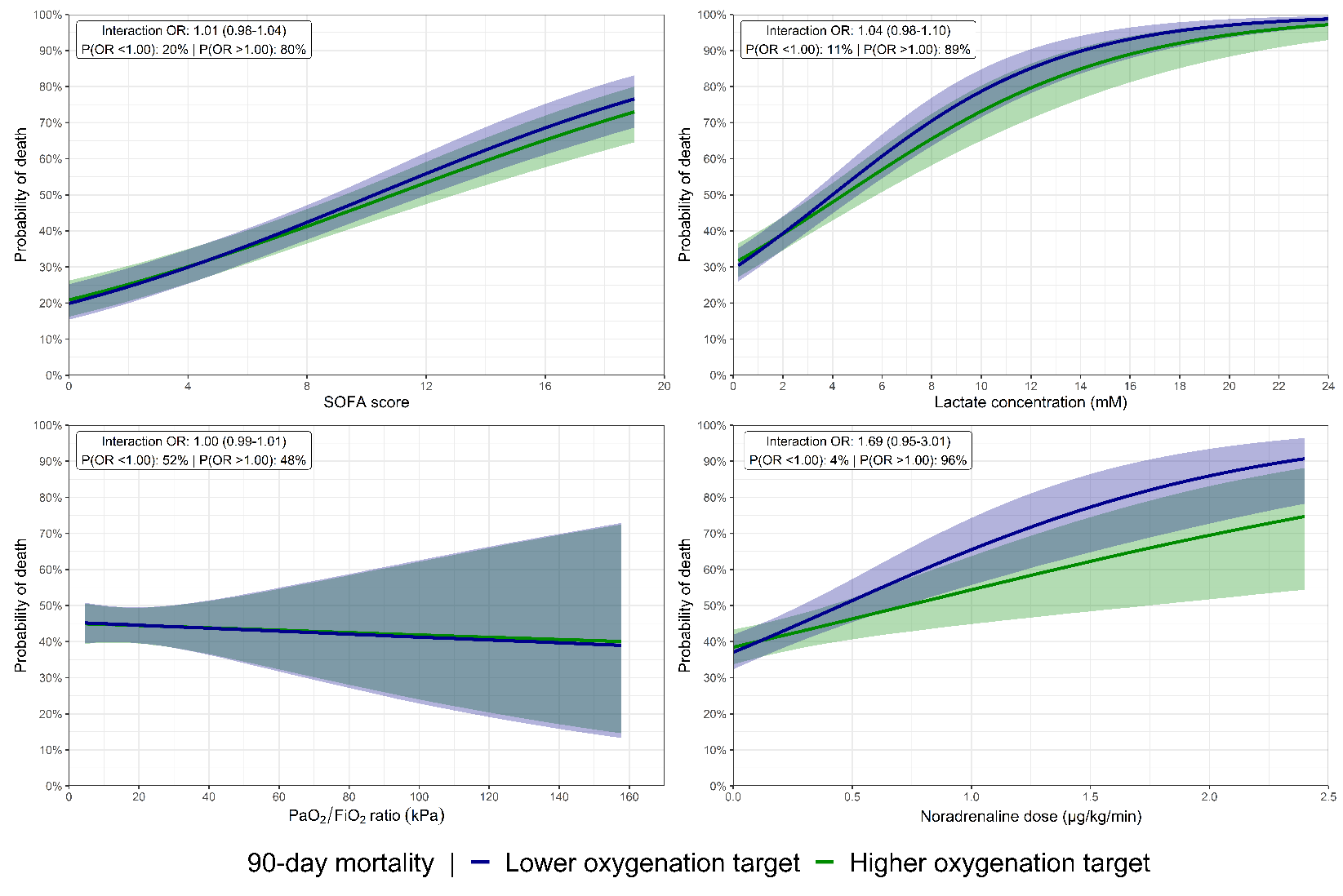


These plots illustrate the estimated interactions between treatment allocation and 90-day all-cause mortality on the continuous scale. The level of the individual variables of interest are plotted on the X-axes; the probabilities of mortality are plotted on the Y-axes. Within each subplot the OR with 95% credibility interval for the interaction effect between the lower oxygenation target and the baseline variable assessed is presented. The posterior probabilities that the interaction OR is < 1.00 (negative interaction) or > 1.00 (positive interaction) are also presented. SOFA score denotes Sequential Organ Failure Assessment score, PaO_2_/FiO_2_ ratio partial pressure of arterial oxygen to fraction of inspired oxygen ratio. In total, 95% of patients had a PaO_2_/FiO_2_ ratio < 35.5 kPa.

## Fig. S7b Conditional effects plot for 90-day all-cause mortality in the sensitivity analysis using sceptic priors


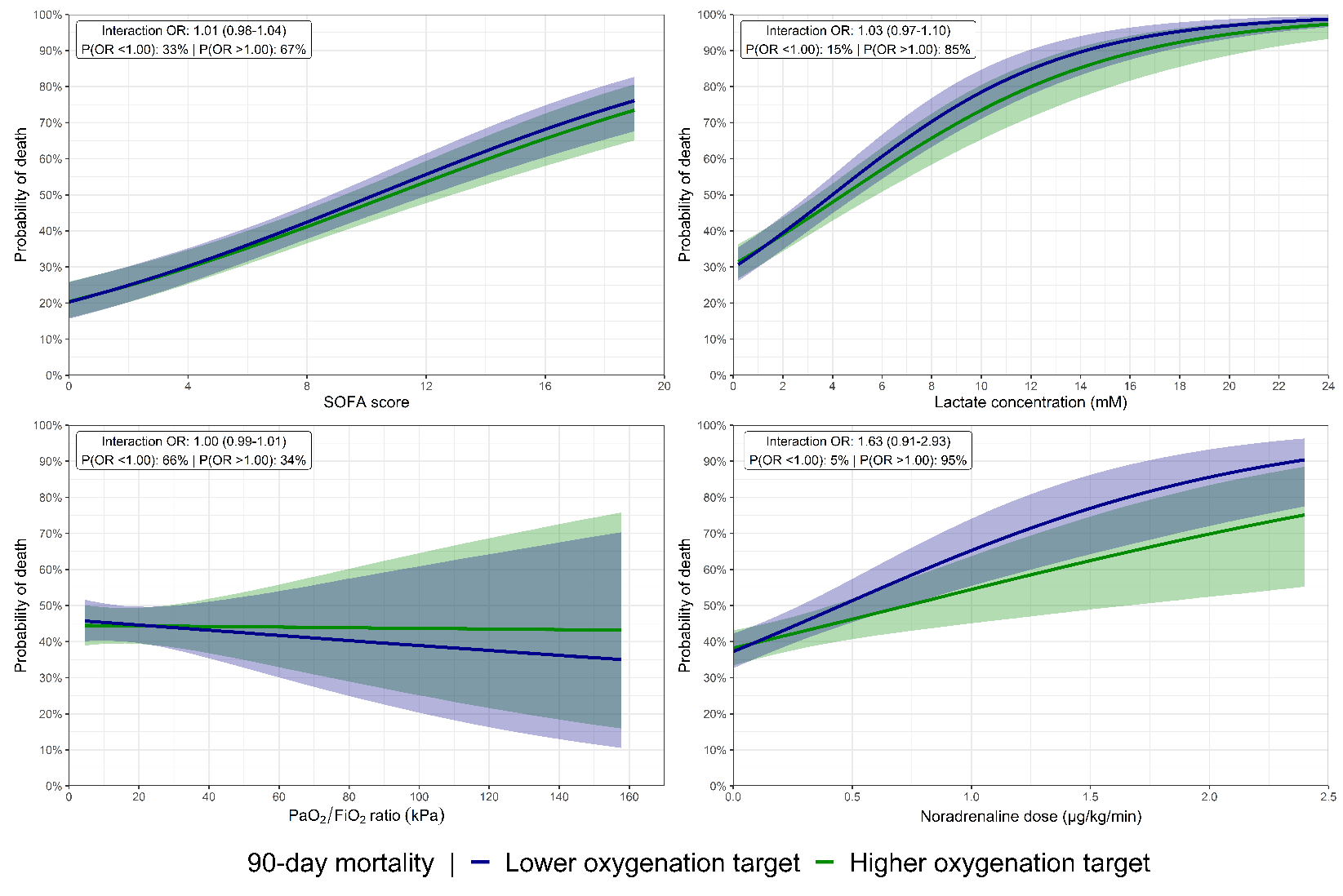


These plots illustrate the estimated interactions between treatment allocation and 90-day all-cause mortality on the continuous scale. The level of the individual variables of interest are plotted on the X-axes; the probabilities of mortality are plotted on the Y-axes. Within each subplot the OR with 95% credibility interval for the interaction effect between the lower oxygenation target and the baseline variable assessed is presented. The posterior probabilities that the interaction OR is < 1.00 (negative interaction) or > 1.00 (positive interaction) are also presented. SOFA score denotes Sequential Organ Failure Assessment score, PaO_2_/FiO_2_ ratio partial pressure of arterial oxygen to fraction of inspired oxygen ratio. In total, 95% of patients had a PaO_2_/FiO_2_ ratio < 35.5 kPa.

## STROBE checklist

Completed Strengthening the Reporting of Observational Studies in Epidemiology (STROBE) statement checklist ^15^ for this manuscript.

STROBE Statement—Checklist of items that should be included in reports of ***cohort studies***

|  | Item No | Recommendation |
| --- | --- | --- |
| **Title and abstract** | 1 | (*a*) Indicate the study’s design with a commonly used term in the title or the abstract – pages 1-2 |
|  |  | (*b*) Provide in the abstract an informative and balanced summary of what was done and what was found – page 2 |
| Introduction | | |
| Background/rationale | 2 | Explain the scientific background and rationale for the investigation being reported – pages 4-5 |
| Objectives | 3 | State specific objectives, including any prespecified hypotheses – pages 4-5 |
| Methods | | |
| Study design | 4 | Present key elements of study design early in the paper – pages 6-8 |
| Setting | 5 | Describe the setting, locations, and relevant dates, including periods of recruitment, exposure, follow-up, and data collection – page 6 |
| Participants | 6 | (*a*) Give the eligibility criteria, and the sources and methods of selection of participants. Describe methods of follow-up – page 6 + page 4 supplementary appendix |
|  |  | (*b*) For matched studies, give matching criteria and number of exposed and unexposed – N/A |
| Variables | 7 | Clearly define all outcomes, exposures, predictors, potential confounders, and effect modifiers. Give diagnostic criteria, if applicable – pages 6-8 + pages 5-6 supplementary appendix |
| Data sources/ measurement | 8* | For each variable of interest, give sources of data and details of methods of assessment (measurement). Describe comparability of assessment methods if there is more than one group – page 6 |
| Bias | 9 | Describe any efforts to address potential sources of bias – N/A |
| Study size | 10 | Explain how the study size was arrived at – page 9S |
| Quantitative variables | 11 | Explain how quantitative variables were handled in the analyses. If applicable, describe which groupings were chosen and why – pages 7-8 |
| Statistical methods | 12 | (*a*) Describe all statistical methods, including those used to control for confounding – pages 6-8 + pages 7-9 supplementary appendix |
|  |  | (*b*) Describe any methods used to examine subgroups and interactions – pages 7-8 + pages 7-9 supplementary appendix |
|  |  | (*c*) Explain how missing data were addressed – page 8 + page 10 supplementary appendix |
|  |  | (*d*) If applicable, explain how loss to follow-up was addressed – N/A |
|  |  | (*e*) Describe any sensitivity analyses – page 7 + page 7-9 supplementary appendix |
| Results | | |
| Participants | 13* | (a) Report numbers of individuals at each stage of study—e.g. numbers potentially eligible, examined for eligibility, confirmed eligible, included in the study, completing follow-up, and analysed – page 9, CONSORT diagram not applicable in secondary analysis |
|  |  | (b) Give reasons for non-participation at each stage – N/A |
|  |  | (c) Consider use of a flow diagram – N/A |
| Descriptive data | 14* | (a) Give characteristics of study participants (eg demographic, clinical, social) and information on exposures and potential confounders – pages 11-26 supplementary appendix |
|  |  | (b) Indicate number of participants with missing data for each variable of interest – page 10 + page 10 supplementary appendix |
|  |  | (c) Summarise follow-up time (eg, average and total amount) – page 6 |
| Outcome data | 15* | Report numbers of outcome events or summary measures over time – page 6 + page 5 supplementary appendix |
| Main results | 16 | (*a*) Give unadjusted estimates and, if applicable, confounder-adjusted estimates and their precision (eg, 95% confidence interval). Make clear which confounders were adjusted for and why they were included – pages 9-10 |
|  |  | (*b*) Report category boundaries when continuous variables were categorized – page 9 |
|  |  | (*c*) If relevant, consider translating estimates of relative risk into absolute risk for a meaningful time period – page 20 + page 27 supplementary appendix |
| Other analyses | 17 | Report other analyses done—eg analyses of subgroups and interactions, and sensitivity analyses – pages 9-10 + 17-22 + pages 27-53 supplementary appendix |
| Discussion | | |
| Key results | 18 | Summarise key results with reference to study objectives – pages 11-12 |
| Limitations | 19 | Discuss limitations of the study, taking into account sources of potential bias or imprecision. Discuss both direction and magnitude of any potential bias – pages 12-13 |
| Interpretation | 20 | Give a cautious overall interpretation of results considering objectives, limitations, multiplicity of analyses, results from similar studies, and other relevant evidence – pages 11-13 |
| Generalisability | 21 | Discuss the generalisability (external validity) of the study results – pages 11-13 |
| Other information | | |
| Funding | 22 | Give the source of funding and the role of the funders for the present study and, if applicable, for the original study on which the present article is based – page 6 + page 4 supplementary appendix |

*Give information separately for exposed and unexposed groups.

**Note:** An Explanation and Elaboration article discusses each checklist item and gives methodological background and published examples of transparent reporting. The STROBE checklist is best used in conjunction with this article (freely available on the Web sites of PLoS Medicine at http://www.plosmedicine.org/, Annals of Internal Medicine at http://www.annals.org/, and Epidemiology at http://www.epidem.com/). Information on the STROBE Initiative is available at http://www.strobe-statement.org.

## References

1. Schjørring OL, Perner A, Wetterslev J, et al. Handling Oxygenation Targets in the Intensive Care Unit (HOT‐ICU)—Protocol for a randomised clinical trial comparing a lower vs a higher oxygenation target in adults with acute hypoxaemic respiratory failure. *Acta Anaesthesiol Scand* 2019; 63: 956–65

2. Schjørring OL, Klitgaard TL, Perner A, et al. Lower or Higher Oxygenation Targets for Acute Hypoxemic Respiratory Failure. *N Engl J Med* 2021; 384: 1301–11

3. Ranieri VM, Rubenfeld GD, Thompson BT, et al. Acute respiratory distress syndrome: The Berlin definition. *JAMA - J Am Med Assoc* 2012; 307: 2526–33

4. Vincent J-L, Moreno R, Takala J, et al. The SOFA (Sepsis-related Organ Failure Assessment) score to describe organ dysfunction/failure. *Intensive Care Med* 1996; 22: 707–10

5. Carpenter B, Gelman A, Hoffman MD, et al. Stan: A probabilistic programming language. *J Stat Softw* 2017; 76

6. Bürkner PC. brms: An R package for Bayesian multilevel models using Stan. *J Stat Softw* 2017; 80

7. Bürkner PC. Advanced Bayesian multilevel modeling with the R package brms. *R J* 2018; 10: 395–411

8. Klitgaard TL, Schjørring OL, Lange T, et al. Bayesian and heterogeneity of treatment effect analyses of the HOT‐ICU trial – a secondary analysis protocol. *Acta Anaesthesiol Scand* 2020; 9: 1376–81

9. Schjørring OL, Klitgaard TL, Perner A, et al. The Handling Oxygenation Targets in the Intensive Care Unit (HOT-ICU) trial: Detailed statistical analysis plan. *Acta Anaesthesiol Scand* [Internet] 2020; 64: 847–56 Available from: http://www.ncbi.nlm.nih.gov/pubmed/32068884

10. Gelman A, Carlin JB, Stern HS, Dunson DB, Vehtari A, Rubin DB. Bayesian Data Analysis. 3rd ed. CRC Press; 2013.

11. Vehtari A, Gelman A, Simpson D, Carpenter B, Bürkner P-C. Rank-normalization, folding, and localization: An improved Rhat for assessing convergence of MCMC. *arXiv* [Internet] 2019; 1–26 Available from: http://arxiv.org/abs/1903.08008

12. Kruschke J. Doing Bayesian Data Analysis - A Tutorial with R, JAGS, and Stan. 2nd ed. Proc. Annu. Conv. Japanese Psychol. Assoc. Academic Press; 2014.

13. Vehtari A, Gelman A, Gabry J. Practical Bayesian model evaluation using leave-one-out cross-validation and WAIC. *Stat Comput* Springer US; 2017; 27: 1413–32

14. Lambert B. A Student’s Gudie to Bayesian Statistics. 1st ed. SAGE Publications Ltd.; 2018.

15. von Elm E, Altman DG, Egger M, Pocock SJ, Gøtzsche PC, Vandenbroucke JP. The Strengthening the Reporting of Observational Studies in Epidemiology (STROBE) statement: guidelines for reporting observational studies. *J Clin Epidemiol* Elsevier Ltd; 2008; 61: 344–9
